# A 38-plex PCR MALDI-TOF MS-based assay to detect SNPs common in elite athletes

**DOI:** 10.1371/journal.pone.0339384

**Published:** 2025-12-29

**Authors:** Miftahul Zannah, Ioannis Papadimitriou

**Affiliations:** 1 Graduate Program in Molecular Medicine, Faculty of Science, Mahidol University, Bangkok, Thailand; 2 Department of Physiology, Faculty of Science, Mahidol University, Bangkok, Thailand; GLA University, INDIA

## Abstract

There is great demand for a novel technique to facilitate the rapid identification of multiple single-nucleotide polymorphisms (SNPs) prevalent in elite athletes. Case-control and genome-wide association studies (GWAS) have been conducted to investigate an individual’s likelihood for success in various sports, revealing several putative loci associated with elite athletic status. However, it remains challenging to detect multiple such SNPs simultaneously with the aim of examining their influence on specific physical fitness characteristics, such as muscle power, strength, or endurance. The aim of the present study is to develop a 38-plex PCR amplification assay, integrated with matrix-assisted laser desorption/ionization time-of-flight mass spectrometry (MALDI-TOF MS), for the identification of 38 SNPs linked to elite athletic performance and assess its quantitative performance metrics and high-throughput capabilities within a single 38-plex reaction. The SNPs were chosen based on their high prevalence among elite power athletes, potential influence on muscle power production, and suitability for multiplex PCR amplification. The developed method simultaneously detected the targeted SNPs in a single tube, using a minimum DNA concentration of 10 ng/μL and achieving a total sample call rate of 93.13%. With further research, this new protocol—which integrates the specificity of multiplex PCR and the sensitivity of MALDI-TOF MS—may offer a unique opportunity to deepen our understanding of the genetic basis of physical fitness and may have prospective applications in research initiatives exploring genetic factors that influence athletic performance, e.g., the Speed Gene Study.

## Introduction

Health-related fitness—such as cardiovascular endurance, muscular strength, and endurance—is consistently associated with a reduced risk of chronic diseases, and improved overall health and well-being. Muscle power is classified as a skill-related fitness component, but is also crucial for human health, injury prevention, and longevity [1,2]. The age-related decline of muscle power occurs at over twice the rate of age-related strength loss [3]. Consistent power training can slow the loss of muscle power, prevent sarcopenia, and preserve muscle fibre properties; however, muscle power production is largely influenced by hereditary factors [4]. In twin studies, muscle power exhibits higher estimated heritability than muscle strength and cardiovascular endurance [5]; suggesting that it may be useful to investigate the influence of specific single-nucleotide polymorphisms (SNPs) on muscle power.

Numerous qualitative studies have been conducted to uncover SNPs associated with muscular power production, with a focus on elite power athletic status, by comparing SNP frequencies among cohorts of elite sprinters, endurance athletes, and controls. Investigations of this type have identified SNPs located in multiple genes, including *ACTN3* [6], *MSTN* [7], *IGF1* [8], *CNTFR* [9], *NOS3* [10], and *GALNTL6* [11]. Although quantitative studies offer greater value and less bias, they are less common in this research field [12,13], due to the complexity of data collection and the need for large sample sizes to detect subtle genetic effects of specific SNPs on physiological characteristics, such as muscle power output [14,15] or maximal oxygen consumption [16,17]. Furthermore, the majority of the quantitative studies in the field lack high-throughput genetic analysis for SNP identification [18], have focused on only one or two SNPs because of the limitations of conventional genetic methodologies, such as RFLPs and quantitative PCR (q-PCR), which are time-consuming, labor-intensive, and costly, thereby hindering the simultaneous detection of multiple SNP targets. Next-generation sequencing technology enables high-throughput identification; however, such methods necessitate a complex process of library preparation and result interpretation. Therefore, it is crucial to develop and introduce in the field a simple high-throughput identification method for rapid and simultaneous detection of multiple SNPs that influence specific physical fitness characteristics, such as muscle strength, power or endurance.

The present study, aims to develop a single-tube multiplex PCR-based amplification assay, integrated with MALDI-TOF MS, for the detection of the following thirty-eight SNPs: rs10186876, rs11091046, rs1137070, rs11549465, rs12778366, rs13135092, rs143384, rs17602729, rs1801131, rs1801282, rs1805065, rs1805086, rs1815739, rs2070744, rs2275998, rs2290463, rs2439823, rs2854464, rs2920503, rs303760, rs3213537, rs3758391, rs4074992, rs41274853, rs4253778, rs4734621, rs55743914, rs558129, rs56068671, rs671, rs680, rs6905419, rs699, rs699785, rs7247312, rs7832552, rs8111989, and rs9320823, and to evaluate its performance by analyzing quantitative metrics such as call rates and error rates within a single reaction. Each of these SNPs exhibited a high prevalence among elite power-oriented athletes [6–11]. Some of these SNPs may also impact the mechanical properties of muscles in the general population as shown in the Speed Gene Study, an interdisciplinary research initiative with the aim of quantitatively determining how much of an individual’s muscle power variability can be attributed to certain genetic polymorphisms [19]. The present study involved the development of a custom panel of SNPs that have been previously reported as common among elite power oriented athletes, and that may influence measurable performance traits, e.g., power and torque production, in the general population. These SNPs were selected based on their high frequency in studies of elite athletes, their potential influence on muscle strength and power production, and their primer suitability for multiplex PCR amplification within the maximum testing ability of MALDI-TOF MS. This technique facilitates the parallel amplification of 38 specific DNA sequences in a single reaction, resulting in the creation of amplicons. Subsequently, these amplicons serve as templates for a single-nucleotide extension reaction, and the end-result products with defined molecular masses are analyzed via MALDI-TOF MS [20]. This distinctive technology facilitates the detection and analysis of specific genes according to molecular weight, and may offer numerous benefits in sports-related applications compared to traditional genotyping methods, which will be highlighted and discussed in this study.

## Methods

All human research procedures followed were in accordance with the ethical standards of the committee responsible for human experimentation (institutional and national), and with the Helsinki Declaration of 1975, as revised in 2013. All methods used in this study were approved by the Human Research Ethics Committee at Mahidol University (Ethics Application ID: MU-CIRB 2024/417.2410) and carried out in accordance with relevant guidelines and laws which apply in Thailand and with the Helsinki Declaration of 1975, as revised in 1983. No animals were used in this research. The protocol described in this peer-reviewed article including DNA isolation and primer design is deposited on protocols.io (DOI: dx.doi.org/10.17504/protocols.io.eq2ly4b5elx9/v1) and is included for printing purposes as supporting information S1 File.

### Genomic DNA isolation

Forty-six healthy male Thais (for ≥3 generations) with a mean age of 25.34 years and a body mass index (BMI) of 23 kg/m², were included in this study to ensure both body composition and genetic homogeneity. The blood (200 μl) was collected duals using spring loaded lancets with glass capillary tubes and stored in EDTA tubes at 5 C^o^ for a maximum of 3 days prior to DNA extraction. All participants provided written informed consent before the blood collection. The recruitment of participants took place from March to September 2025. The DNA was isolated from white blood cells using bead-based DNA separation methods as suggested by [18]. Briefly, the process involved adding magnetic beads to the sample, where the DNA binds to the beads. The beads were then separated from the sample using a magnetic field, allowing for the removal of contaminants and the isolation of pure DNA. Using this method for the 46 DNA samples included in this study we generated yields up to 160 ng/μl. The sbeadex ^TM^ blood DNA Kit (Biosearch Technologies, Germany) was used for DNA extraction following the manufacturer’s protocol, yielding highly pure genomic DNA suitable for primer specificity testing and method development experiments. The quality of extracted DNA was evaluated using a NanoDrop spectrophotometer (Thermo Fisher Scientific Inc., Massachusetts, United States). Samples with an OD260/280 ratio ranging from 1.8 to 2.0 and an OD260/230 ratio within the range of 2.0 to 2.2 were chosen for subsequent experiments.

### Primer design

The primer design included obtaining a considerable amount of DNA sequences for every targeted locus from the NCBI genome database [21]. The DNA sequences were subsequently aligned using the CLUSTAL-W alignment software [22] to pinpoint highly conserved areas for primer design with the Assay Design 4.0 program (Agena Bioscience, Inc., California, United States). All targeted gene sequences were entered into the program, adhering to the manufacturer’s guidelines. Moreover, the primer design method followed rigorous criteria to reduce primer-dimer formation, hairpin loops, and non-specific priming, hence guaranteeing the synthesis of excellent quality primers. Furthermore, the specificity of the synthesized primers was assessed using the BLAST (Basic Local Alignment Search Tool) software to verify optimal primer specificity. Only primers demonstrating the maximum specificity were chosen for the multiplex PCR based protocol developed in this study. It is essential to note here that manual modifications, including base alterations and primer sequence changes, were carried out when required to attain uniformity in guanine-cytosine content (%GC) values and melting temperature (Tm) among multiplex primers, hence optimizing PCR amplification protocol. All primers were synthesized by Macrogen, Inc. in Seoul, Korea.

A total of 38 multiplex-PCR primer pairs were developed for the assay panel utilizing the Assay Design Suite v4.0. The lengths ranged from 142 to 88 bp, with melting temperatures (Tm) varying between 45.7 and 66.5°C, and GC content percentages fluctuating from 15 to 88.2%. Thirty-eight PCR products measuring between 88 and 142 bp were synthesized, to produce non-overlapping SBE products with molecular masses spanning from 4150.7 to 7738.1 Da, and a molecular mass range of 4462.9 to 8065.2 Da, all based upon the selected sequence. The melting temperatures (Tm, °C), guanine-cytosine content (%GC) of each targeted SNP, the molecular masses of the extension primers and SBE products are shown in the supplementary data provided in supporting information file S2 Table, while the nucleotide sequences of the primers are presented in Table 1 below.

**Table 1:** The PCR primers (forward, reverse and extension) sequence of 38 SNPs linked to athletic success in power-related sports.

*Targeted SNPs*	*Forward Primer Sequence*	*Reverse Primer Sequence*	*Extended Primer Sequence*
rs11091046	ACGTTGGATGGGATTATTCAGGCTTTAGGC	ACGTTGGATGCCTCCACTCAAGTGAAATGC	AGGAATATAATTTATAGC
rs1137070	ACGTTGGATGCTTAAATGGTCTCGGGAAGG	ACGTTGGATGCCAGAGTCACCAAACTTACC	tGGTGACCGAGAAAGA
rs11549465	ACGTTGGATGCTTCCAGTTAC GTTCCTTCG	ACGTTGGATGCTTTGAGGACTTGCGCTTTC	TCGATCAGTTGTCA
rs12778366	ACGTTGGATGGCTAAGGTCCTATCTACATC	ACGTTGGATGTAAGGCTTCTAGGACTGGAG	TCTTATTTCATCTGGTCACCACT
rs13135092	ACGTTGGATGCCGTCACATAAACAGAACC	ACGTTGGATGTTAGCTTGAAAGGGTGTTG	ggacGGGTGTTGAATTTTA
rs143384	ACGTTGGATGCGCTGAATGACACCAAAGAG	ACGTTGGATGCCTTTCATGGTTTTTCCTGC	ACCAGAGGCACCTT
rs17602729	ACGTTGGATGCTGACAAATGGCAGCAAAAG	ACGTTGGATGGCCACCATGATTACAGAAAG	gttaATACAGCTGAAGAGAAA
rs1801131	ACGTTGGATGCCGAGAGGTAAAGAACGAAG	ACGTTGGATGTCTACCTGAAGAGCAAGTCC	ggGACTTCAAAGACACTT
rs1801282	ACGTTGGATGGTTATGGGTGAAACTCTGGG	ACGTTGGATGGTTTGCAGACAGTGTATCAG	GGAGATTCTCCTATTGAC
rs1805065	ACGTTGGATGTCCAGGTGCCTTCTTGATCC	ACGTTGGATGCCAGCTCCATGTAGAACAG	CTTGATCCCGTACA
rs1805086	ACGTTGGATGGACGGGTCTCAAATATATCC	ACGTTGGATGGTGGATGGAAAACCCAAATG	agACAATACAATAAAGTAGTAA
rs1815739	ACGTTGGATGCGATCAGTTCAAGGCAACAC	ACGTTGGATGCAGATCTTCTGGATCTCACC	gACTGCCCGAGGCTGAC
rs2070744	ACGTTGGATGACTGTAGTTTCCCTAGTCCC	ACGTTGGATGAGGTCAGCAGAGAGACTAGG	CAAGCTCTTCCCTGGC
rs2275998	ACGTTGGATGCTACGTTATTACACCGACGC	ACGTTGGATGAAGCCTCATCTGCTAAGGTG	gcgtCCTAGCTCGTCCTAGGG
rs2290463	ACGTTGGATGAAGGTGGAGGTAAGGGCTG	ACGTTGGATGAGCATGAGGGCTCCCAACT	ccccTCCCCCCAGGTTGGA
rs2439823	ACGTTGGATGTAAGTGAGTGACAGGGAAGG	ACGTTGGATGAGACTATCTCACCTTTCAGC	ACCTTTCAGCTCTCTA
rs2854464	ACGTTGGATGGAATCCTGGTGGAAGTCTTG	ACGTTGGATGGATGCTGCTGAGGATGATG	GGCTGGCTCATTTCCCA
rs2920503	ACGTTGGATGCTCAGTGGGTTTTTCGAAC	ACGTTGGATGTTCTGGGACATTTTAATGG	cTTAATCTTGATTATATTCAA
rs303760	ACGTTGGATGTCTTCGATGAGGCCAACAAG	ACGTTGGATGGACAGGACAGGAGGGGACG	ggtaGCGCGGGACCGGCCTTGGC
rs3213537	ACGTTGGATGAAGAGGCCAGAGAGTATGAG	ACGTTGGATGGGGAGGAGAGAGCTTTTAAG	ATGAGGGTCATGGT
rs3758391	ACGTTGGATGGCCATAACAAACACTGGCTC	ACGTTGGATGGCACACTGTGACTCCATATC	CAAACACTGGCTCTAGATCTACCA
rs4074992	ACGTTGGATGTCATCGTCATGACCACCAG	ACGTTGGATGTTGGGCAGCCGAAGATGCAC	ttggGCGGTGCCAGTGCTC
rs41274853	ACGTTGGATGTCTTTGGTGGGTGGGTTAG	ACGTTGGATGGACACAATTTGTGGAGACCC	cGCCGCCCCCCAGCCCTG
rs4253778	ACGTTGGATGGGTGGAACACTTGAAGCTTG	ACGTTGGATGTTCTGGAGATCACAACCACC	ttttAAGCTTGATATCTAGTTT
rs4734621	ACGTTGGATGACCACCACACACAGCTAATC	ACGTTGGATGCAGCCTGGGCATTATAGTG	TGTTTATTTTTTTGTAGAAA
rs55743914	ACGTTGGATGACTTTCTTTCTTACTCTGC	ACGTTGGATGGTCAAGGCTCGTAAAGTCAG	gTTCTTACTCTGCATACAG
rs558129	ACGTTGGATGGATCTTAAAATGCAGAGGTC	ACGTTGGATGATTCCCCAGTGTGTCTTTTG	cttaCCTAAATTCAATCACAA
rs56068671	ACGTTGGATGCAGAAAGAGATATACTGTGG	ACGTTGGATGTTCTTGGTGAATTGACTCC	GGAAAATTAAGCTAAG
rs671	ACGTTGGATGAGGTCCCACACTCACAGTTT	ACGTTGGATGCCTTTGGTGGCTACAAGATG	CCCACACTCACAGTTTTCACTT
rs680	ACGTTGGATGAAATTCCCGTGAGAAGGGAG	ACGTTGGATGTCCCTGAACCAGCAAAGAG	AAGAGAAAAGAAGG
rs6905419	ACGTTGGATGTATCGCCCAGGCTGGAATG	ACGTTGGATGAGGCTGAAGCAGGAGAATG	ATCTCGGCTCACTG
rs699	ACGTTGGATGGTGGACGTAGGTGTTGAAAG	ACGTTGGATGCTGTGACAGGATGGAAGAC	gGGAAGACTGGCTGCTCCCTGA
rs699785	ACGTTGGATGGAGTGCAATGGTGCAATCTC	ACGTTGGATGTAATCCTGGCTACTTGGGAG	cttCTCATCGCAAACTCC
rs7247312	ACGTTGGATGAGATCACACCACTGCACTC	ACGTTGGATGCAAAGTGGGTCAACTGGAAC	ctccTTTTCTGTCTCTTTTT
rs7832552	ACGTTGGATGGCCTTGACCTCAAAGGAATG	ACGTTGGATGACAACAAGAGTCAAGCACCC	GATAGTGTGAGGTA
rs8111989	ACGTTGGATGAGCTTTCTAGGAGAAATGGG	ACGTTGGATGCTGACTTCATCCCTCTGTAG	aagTTCTCAAGAACCTGCC
rs9320823	ACGTTGGATGCTTGGGTGGCTTCAAACTAC	ACGTTGGATGAGAAATGAGGGAGATAAGGG	ttGCCTTTTTCTGTCATGAA

### Protocol optimization

The custom assay panel of the previously reported SNPs related to athletic success in power related sports was optimized and adjusted according to the manufacturer’s guidelines. Briefly, the 5 μL PCR reaction mix contained 0.50 μL PCR Buffer, 0.40 μL MgCL_2_, 0.10 μL dNTPs and 0.20 PCR Enzyme (Agena Bioscience, California, United States), 1 μL of primer mixture yielding a final concentration of 500 nM for each forward and reverse primer, 2.0 μL of DNA template, and 0.80 μL of DNase-free distilled water. The multiplex PCR amplification assay was performed with a MiniAmp™ Thermal Cycler (Thermo Fisher Scientific, Waltham, MA, USA) in our laboratory at Faculty of Science, Mahidol University. The initial PCR involved a denaturation at 95°C for 2 minutes, followed by 45 cycles of denaturation at 95°C for 30 seconds, annealing at 55°C for 30 seconds, extension at 72°C for 1 minute, and a final extension at 72°C for 5 minutes as shown in the supplementary data provided as supporting information S1 File. The reaction was finally terminated by reducing the temperature at 4°C to end the amplification process. After the initial PCR reaction, the unincorporated dNTPs were removed using shrimp alkaline phosphatase (SAP) (Agena Bioscience, California, United States). The SAP reaction mix contained 0.17 μL SAP enzyme, 0.30 μL SAP Buffer and 1.53 μL of DNase-free distilled water. The dephosphorylation reaction was performed with a MiniAmp™ Thermal Cycler (Thermo Fisher Scientific, Waltham, MA, USA) and involved an initial stage at 37°C for 40 minutes, followed by inactivation at 85°C for 5 minutes. Subsequent to the SAP reaction, the single-base extension (SBE) reaction was also performed using a MiniAmp™ Thermal Cycler (Thermo Fisher Scientific, Waltham, MA, USA) in our laboratory with a mixture of extension reaction mix synthesized in accordance with the iPLEX® Pro and Gold Reagents User Guide [23,24] containing 0.2 μL iPLEX® Pro Buffer, 0.2 μL iPLEX® Pro Termination Mix, 0.04 μL, iPLEX® Pro Enzyme, 0.94 μL Extension Primer and 0.62 of DNase-free distilled water. Briefly, the SBE reaction begun with an initial step at 95°C for 30 seconds, continued by 40 cycles including denaturation at 95°C for 5 seconds, and 5 cycles of an annealing phase at 52°C for 5 seconds and a denaturation phase at 80°C for 5 seconds. The extension was conducted at 72°C for 3 minutes, followed by a hold at 4°C overnight as shown in the supplementary data provided as supporting information S1 File.

The next day, the SBE products were dispensed onto the SpectroCHIP via a nano-dispenser and then loaded into the mass spectrometer (MS) for the determination of the molecular mass of the SBE products (Agena Bioscience, California, United States). The molecular mass and nucleotide related data for individual SBE products obtained from the MS were evaluated and interpreted using MassARRAY® Typer Viewer v4.0 software (Agena Bioscience, California, United States).

## Results

**Fig 1:**
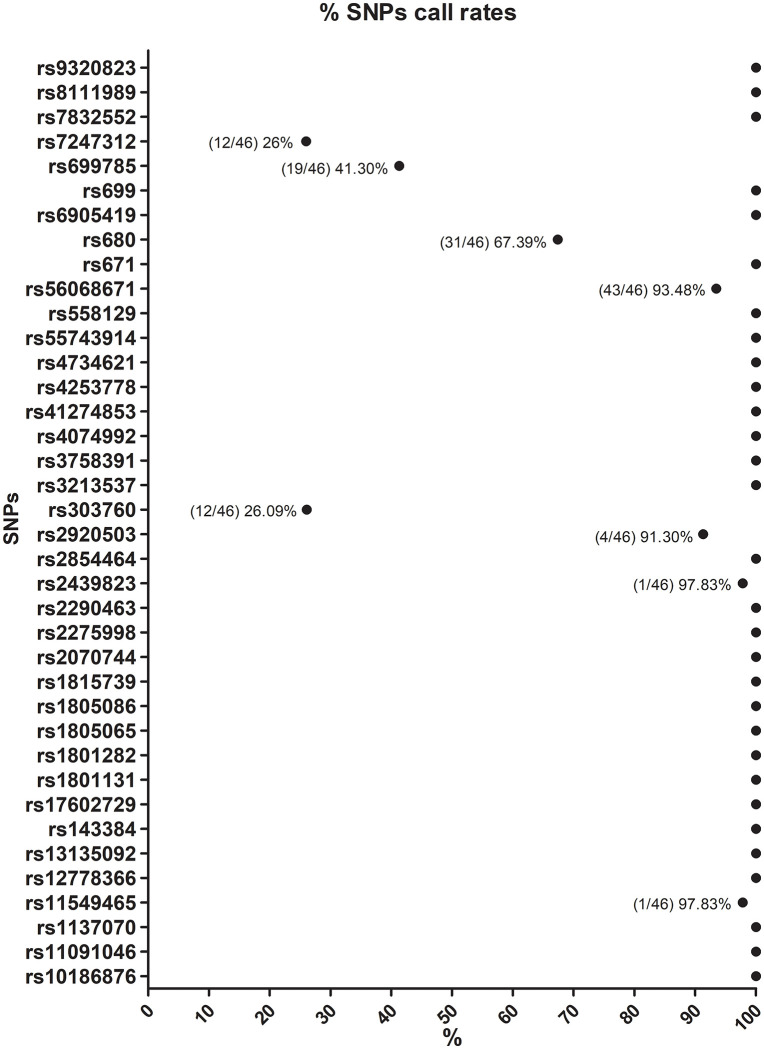
The % call rates of 38 targeted SNP in 46 Thai participants included in this study.

This study established a tailored assay panel incorporating SNPs previously linked to success in power-oriented sports, achieving a sample call rate of 93.13%. Thirty SNPs displayed a call rate of 100%, whereas only three SNPs (rs303760, rs699785, and rs7247312) displayed call rates below 50%. The qualitative metrics for each sample are included in the supplemental data file S3 Table. The chromatograms for each targeted SNP, are provided in the supplemental data file S4 Fig, while quantitative metrics, including call rates and error rates, are summarized in Fig 1 below.

**Fig 2:**
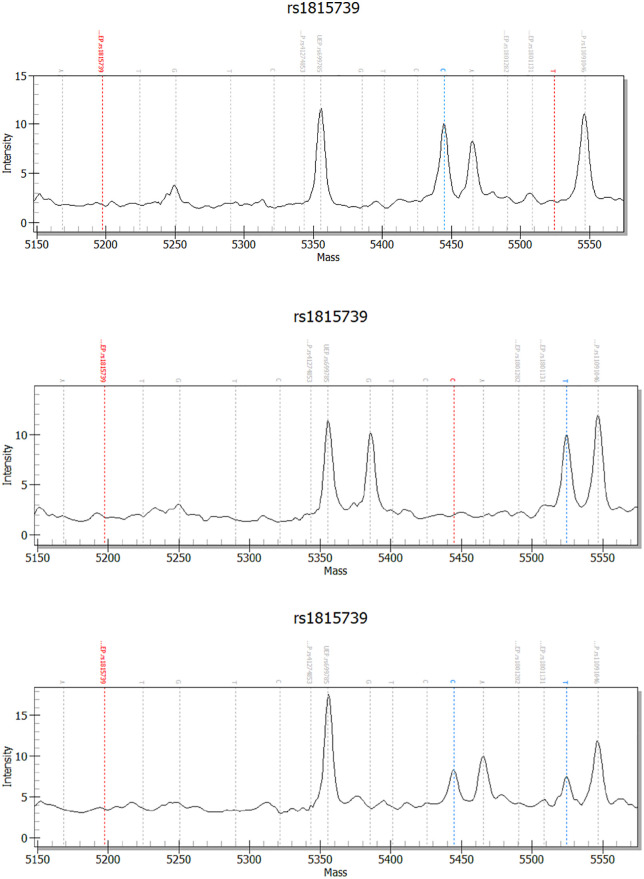
Chromatograms show the mass spectra of one of the commonly targeted SNPs in the field, the *ACTN3* rs1815739 arg577ter C/T polymorphism. a) The wild type allele homozygotes, RR, are indicated by a blue dotted line. b) The mutant allele for XX homozygotes is shown by a blue dotted line. c) The RX heterozygotes are indicated with two blue dotted lines in the graph. The x-axis represents molecular mass (Da), whereas the y-axis indicates peak intensity. The precise molecular mass for wild type allele is 5444.6 (Da) and for the mutant allele is 5524 (Da). All chromatograms for each targeted SNP are provided as supporting information S4 File.

**Fig 3:**
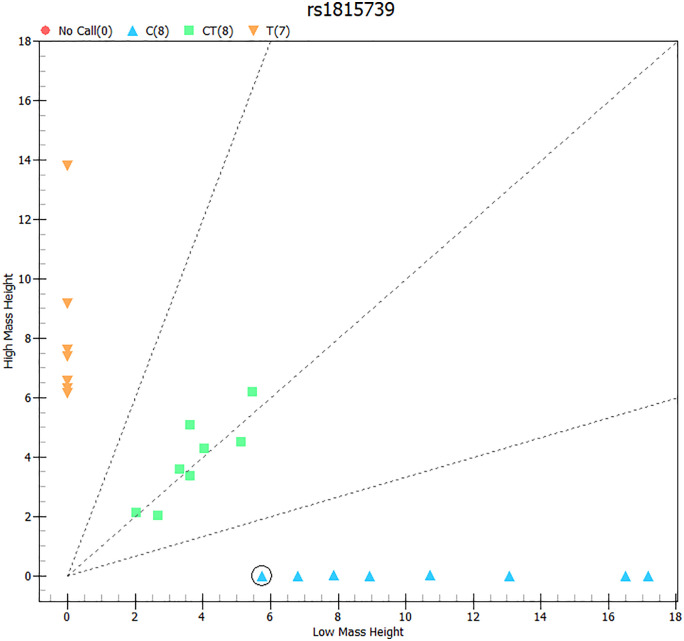
Classification of the three possible *ACTN3* arg577ter C/T genotypes based on their molecular mass. ACTN3 RR homozygotes are indicated with orange, *ACTN3* RX heterozygotes are shown in green, while *ACTN3* XX homozygotes are shown in blue.

The chromatograms shown in Fig 2 demonstrate successful identification of one of the most commonly targeted SNPs in the field, the *ACTN3* rs1815739 arg577ter C/T polymorphism with the three possible genotypes as detected with this methodology. Furthermore, Fig 3 demonstrates the classification of the three possible *ACTN3* arg577ter C/T genotypes based on their molecular mass in TypeAnalyzer 4.02 by Sequenom Software.

## Discussion

This study introduces to the field of Sport Genomics a single-tube 38-plex PCR assay integrated with MALDI-TOF MS for the detection of SNPs linked to athletic performance. The assay yielded results for numerous targets in a single 38-plex reaction, reaching an overall sample call rate of 93.13%. Thirty SNPs exhibited a call rate of 100%, however three SNPs (rs303760, rs699785, and rs7247312) exhibited call rates below 50%, indicating the extent to which each SNP target was identified in a 38-plex reaction. We are currently further evaluating the assay by genotyping the samples in triplicate while comparing the results with known genotypes. Meanwhile recent research from several other fields indicate that PCR integrated with MALDI-TOF MS exhibits high reliability [25–27], achieving an overall concordance rate of 97.9% with qPCR in the detection of multiple swine viruses from various matrix materials [28], with superior sensitivity and specificity compared to more traditional genotyping techniques [29].

Furthermore, the finding that we identified only one allele in 46 of the samples for the following SNPs: rs10186876, rs13135092, rs17602729, rs1805065, rs1805086, rs4253778, rs6905419, and rs699785 may indicate that these genetic loci demonstrate a low prevalence within the Thai population. This finding aligns with studies in other Asian populations where these variants are uncommon, warranting further investigation with larger sample sizes in Thai population.

Moreover, it is important to acknowledge that the methodology employed in this study has inherent limitations. The utilization of multiplex PCR-based amplification combined with MALDI-TOF MS may be constrained by inadequate sample quantity and quality during the reaction; however, our study indicates that the developed method produced interpretable results for most of 38 targeted SNPs using a DNA concentration of 10 ng/μL in certain samples. Since DNA will degrade over time, it is imperative to use freshly prepared samples, as was done in the present study for all testing protocols. Furthermore, it must be noted that the PCR-based amplification used in this study has various limitations related to multiplex PCR reactions—including that primer competition for specific sequences may result in diminished assay sensitivity, the generation of nonspecific products, or cross-reactivity [29]. This study also aimed to determine possible technical challenges affecting call rate, such primer dimers, cross-reactivity, and reagent limits, prior to implementing this approach on a larger scale. This work employed the Assay Design 4.0 tool, applying stringent criteria to mitigate issues associated with secondary structures, homodimers, and heterodimers. Moreover, precise calibration of the extension primer (EP) concentrations is crucial in the MALDI-TOF MS technique, since insufficient EP concentrations may produce atypical primer peaks during the experiment, potentially resulting in assay errors. This study conducted a calculation of EP reaction mix concentration based on the quantity of multiplex reactions, as outlined previous studies [23,24], in order to optimize the quantity of each EP for a 38-plex reaction. These adjustments were critical for smooth implementation of this genetic analysis using multiplex PCR integrated with MALDI-TOF MS [30].

With further research, the 38-plex PCR protocol combined with MALDI-TOF MS for SNP detection related to elite athletic performance presented in this study may enable more effective exploration of the influences of SNPs on muscle performance and biomechanics. Biomechanomics is an emerging research field that integrates genomics and biomechanics, with the goal of identifying genetic variations that influence mechanical properties of the muscle, including power production, acceleration, velocity, and torque [18]. While still in its nascent developmental stage, biomechanomics aims to leverage high-throughput genetic analysis to create a comprehensive genetic profile describing all of the mechanical properties of muscles [19,31]. However additional research is necessary to further evaluate the assays reliability before apply this methodology in Biomechanomics.

Furthermore, previous research indicate that specific targeted SNPs associated with wild or mutant alleles of these genes are prevalent among elite sprinters; conversely, alternative alleles are frequently found in elite endurance athletes, with certain variants also influencing muscle strength [32]. Consequently, while the assay developed in this study is explicitly intended for research on muscle power, it may also be applicable for evaluating endurance or strength-related fitness characteristics, depending on the researchers’ interpretation of the results.

Moreover, based on previous research, the 38 SNPs included in the custom assay panel developed in this study may enhance muscle power and other fitness related characteristics that are highly desirable in elite athletes [32]. Therefore, these SNPs are considered potential targets for gene doping, i.e. the non-therapeutic use of genes and genetic elements to enhance athletic performance, which is a significant concern related to the future of sports [33]. Continual advancements in gene therapy increase the potential for athletes to misuse these technologies to gain unfair advantage. Moreover, gene doping may pose significant health hazards. The long-term consequences of gene doping remain poorly understood, and concerns have been raised about potential cancer risks and immunological responses [33]. Detecting gene doping is difficult because of the resemblance between naturally produced proteins and those produced through gene doping [34]. Conventional urine and blood analyses may prove inadequate for detecting gene doping, rendering it an attractive option for potential offenders. The World Anti-Doping Agency (WADA) currently faces challenges in requiring all athletes to have their genomes sequenced to help regulators look for illegal modifications for detecting gene doping [35]. With further research this high-throughput methodology—which leverages the specificity of multiplex PCR and the sensitivity of MALDI-TOF MS—provides a rapid approach that may be more applicable than traditional PCR methods for gene doping detection purposes in the future. However further research is necessary to evaluate this assays capabilities including more quantitative performance metrics, while comparing its performance with conventional PCR methodologies before eventually apply this methodology for gene doping detection.

Effective SNP genotyping is crucial in sport genomics research, especially when SNPs lack a known quantitative phenotype, or when the phenotype has only been assessed through qualitative associations with athletic success in studies including elite athletes. Conventional techniques, such as RFLPs, require time-consuming, laborious, and costly procedures, and the evaluation of the results may be subjective [36]. Fluorescence-based quantitative PCR (qPCR) offers enhanced sensitivity and operational simplicity, and is thus extensively used in the field of sport genomics [16]. However, the limited number of accessible fluorescence channels hinders the concurrent detection of many SNP targets. Next-generation sequencing technology enables high-throughput identification, but requires a complex process for library preparation and result interpretation. MALDI-TOF MS offers distinct advantages that enable the simultaneous identification of numerous SNPs common in elite athletes amplified in a single reaction [37]. In recent years, various novel PCR-based techniques have been substantially expanded and become widely utilized in different fields of research. Notably, the combination of multiplex PCR with MALDI-TOF MS technology has arisen as an especially promising method [20]. Multiple studies confirm the adaptability and effectiveness of this technique for accurately detecting numerous SNPs across various research fields. Overall, the newly developed multiplex PCR-based amplification assay integrated with MALDI-TOF MS in this study exhibited promising results for its high-throughput capabilities, enabling simultaneous identification most of the 38 SNPs in a single tube. Additional research should be undertaken to further evaluate this assays capabilities including more quantitative performance metrics, before apply this methodology in sports-related applications.

## Supporting information

S1 File The 38-plex PCR MALDI-TOF MS-based assay. (PDF)

S2 Table The PCR primers, melting temperatures. (PDF)

S3 Table The qualitative data for the 38-plex assay. (XLSX)

S4 Fig All 80 chromatograms for each targeted SNP. (PDF)
