# Supplementary material for: A 38-plex PCR MALDI-TOF MS-based assay to detect SNPs common in elite athletes
Source: PLoS One. 2025 Dec 29;20(12):e0339384. doi: 10.1371/journal.pone.0339384 (PMC12747323; doi:10.1371/journal.pone.0339384)
Supplement: S1 File — (PDF) [file pone.0339384.s001.pdf]

A 38-plex PCR MALDI-TOF MS-based assay to detect SNPs common in elite athletes

RESERVED DOI:
10.17504/protocols.io.eq2ly4b5elx9/v1

Miftahul Zannah[1], Ioannis Papadimitriou[2]

[1]Graduate Program in Molecular Medicine, Faculty of Science, Mahidol University, Bangkok, Thailand;
[2]Department of Physiology, Faculty of Science, Mahidol University, Bangkok, Thailand

Ioannis Papadimitriou: Corresponding author;

ioannis.pap

Protocol Info: Miftahul Zannah, Ioannis Papadimitriou . A 38-plex PCR MALDI-TOF MS-based assay to detect SNPs common in elite athletes. **protocols.io** **https://protocols.io/view/a-38-plex-pcr-maldi-tof-ms-based-assay-to-detect-s-hesbb3eap**

Created: November 03, 2025

Last Modified: November 21, 2025

Protocol Integer ID: 231971

Keywords: DNA isolation, Amplification, Dephosphorylation

Funders Acknowledgements:
All chemical consumables for this study were covered by Faculty of Science Mahidol University and corresponding author's Early Career Research Grand A35/2562.
Grant ID: A35/2562

Abstract

There is great demand for a novel technique to facilitate the rapid identification of multiple single-nucleotide polymorphisms (SNPs) prevalent in elite athletes. Case-control and genome-wide association studies (GWAS) have been conducted to investigate an individual's likelihood for success in various sports, revealing several putative loci associated with elite athletic status. However, it remains challenging to simultaneously detect multiple such SNPs with the aim of examining their influence on specific physical fitness characteristics, such as muscle power, strength or endurance. Here we developed a 38-plex PCR amplification assay, integrated with matrix-assisted laser desorption/ionization time-of-flight mass spectrometry (MALDI-TOF MS), for the identification of 38 SNPs linked to elite athletic performance within a single 38-plex reaction. The SNPs were chosen based on their high prevalence among elite power athletes, potential influence on muscle power production, and suitability for multiplex PCR amplification. The developed protocol simultaneously detected the targeted SNPs in a single tube, using a minimum DNA concentration of 10 ng/μL and achieving a total sample call rate of 93.13%. With further research this new protocol—which integrates the specificity of multiplex PCR and the sensitivity of MALDI-TOF MS—may offer a unique opportunity to deepen our understanding of the genetic basis of physical fitness and may have prospective applications in research initiatives exploring genetic factors that influence athletic performance, e.g. the Speed Gene Study [1].

Guidelines

With further research this protocol can be used for generic profiling of elite athletes.

Troubleshooting

Step 1: The DNA isolation

37m

1 Collect the blood (200 µL) duals using spring loaded lancets with glass capillary tubes and store in EDTA tubes at 5 °C for a maximum of 3 days prior to DNA extraction.

2 Extract the DNA from white blood cells utilizing bead-based DNA separation methods with the Sbeadex™ blood DNA Kit (Biosearch Technologies, Germany) as described below.

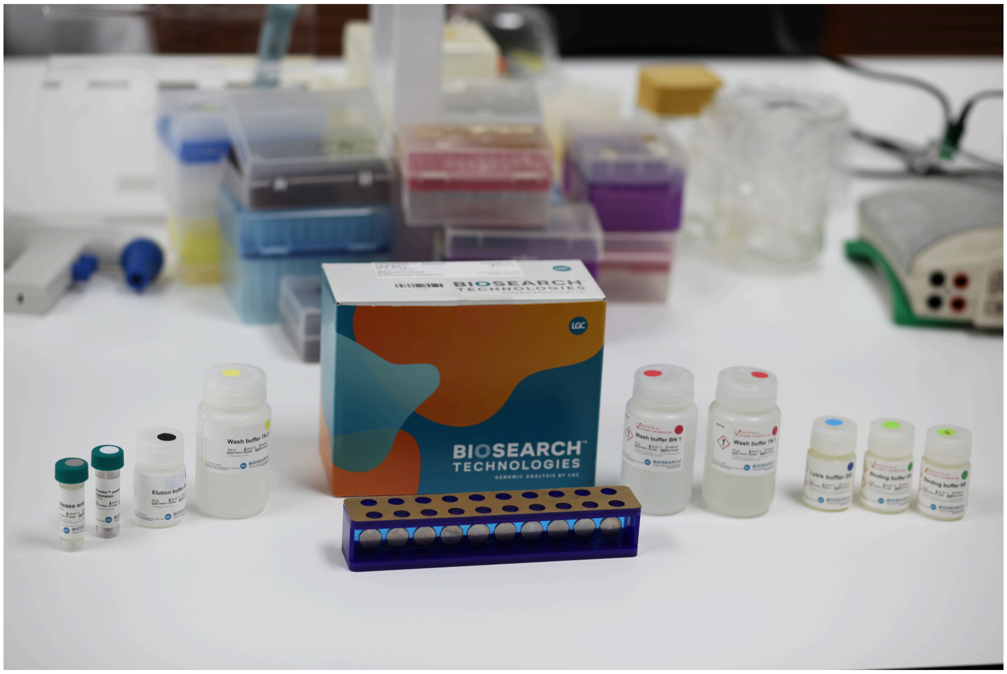

The magnetic rack and the kit were used for the DNA extractions.

3 Initially, add 1x volume of Lysis Buffer to 200 µL of fingertip blood, followed by the addition of 0.2x volumes of Protease K solution.

4 Thereafter incubate the samples at 55 °C for 00:20:00 with constant shaking. 20m

5 Allow the samples to cool briefly to Room temperature , after which add 1.8x the volume of Binding Buffer containing Sbeadex™ magnetic particles. Upon completion of 5m

this step, incubate the samples for 00:05:00 with continuous agitation and place into a magnetic rack, ensuring close proximity to the magnets.

6 Subsequent to this step, discard the supernatant, remove the Eppendorf tube from the magnetic rack, and add 4x volume of Wash Buffer 1 to the sample, followed by 00:05:00 - 00:10:00 incubation with constant shaking. 10m

7 After that, reposition the tube in the magnetic rack, ensuring close proximity to the magnets for approximately 00:02:00 . 2m

8 Then discard the supernatant, and remove the samples from the magnetic rack. This procedure was subsequently conducted once with the inclusion of Wash Buffer 1 & RNase solution, and once more with the addition of Wash Buffer 2.

9 Finally, add 0.25x-1x volume of Elution Buffer to the sample, which was then returned to the magnetic rack.

10 Meticulously transfer the elute to a new Eppendorf tube via pipetting, ensuring that all Sbeadex™ beads remained in the original tube.

11 Evaluate the quality of extracted DNA using a NanoDrop spectrophotometer (Thermo Fisher Scientific Inc., Massachusetts, United States). Choose the samples with an $OD_{260}/_{280}$ ratio ranging from 1.8 to 2.0 and an $OD_{260}/_{230}$ ratio within the range of 2.0 to 2.2 for the subsequent experiments.

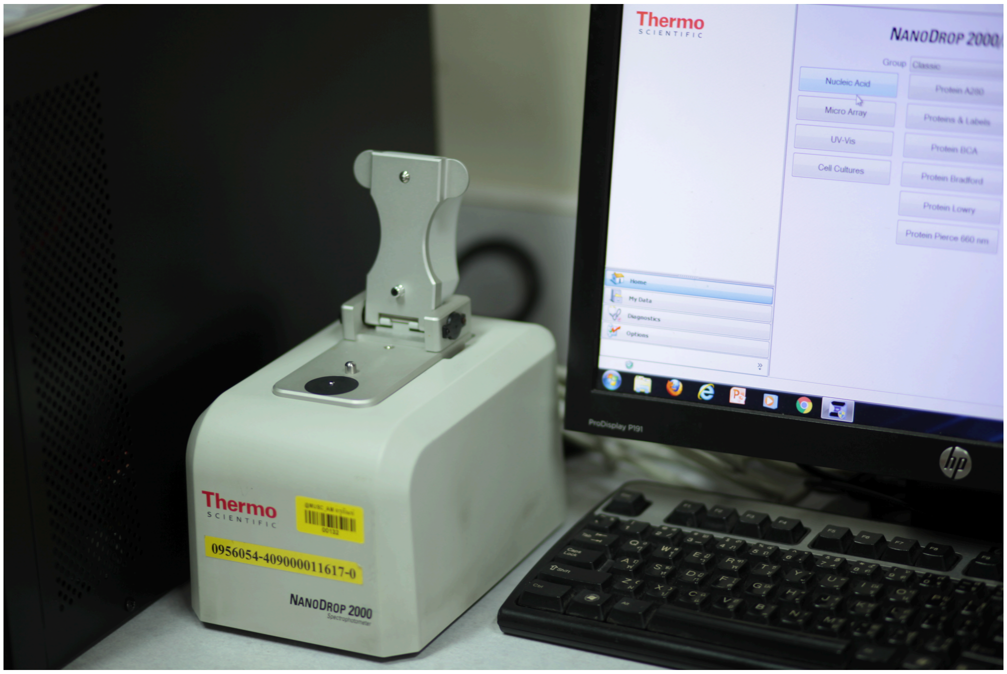

The Nanodrop Spectrophotometer used for the experiment.

Step 2: Primer design

12 The primer design included obtaining a considerable amount of DNA sequences for every targeted locus from the NCBI genome database.

13 Subsequently align the DNA sequences using the CLUSTAL-W alignment software **[2]** to pinpoint highly conserved areas for primer design with the Assay Design 4.0 program (Agena Bioscience, Inc., California, United States).

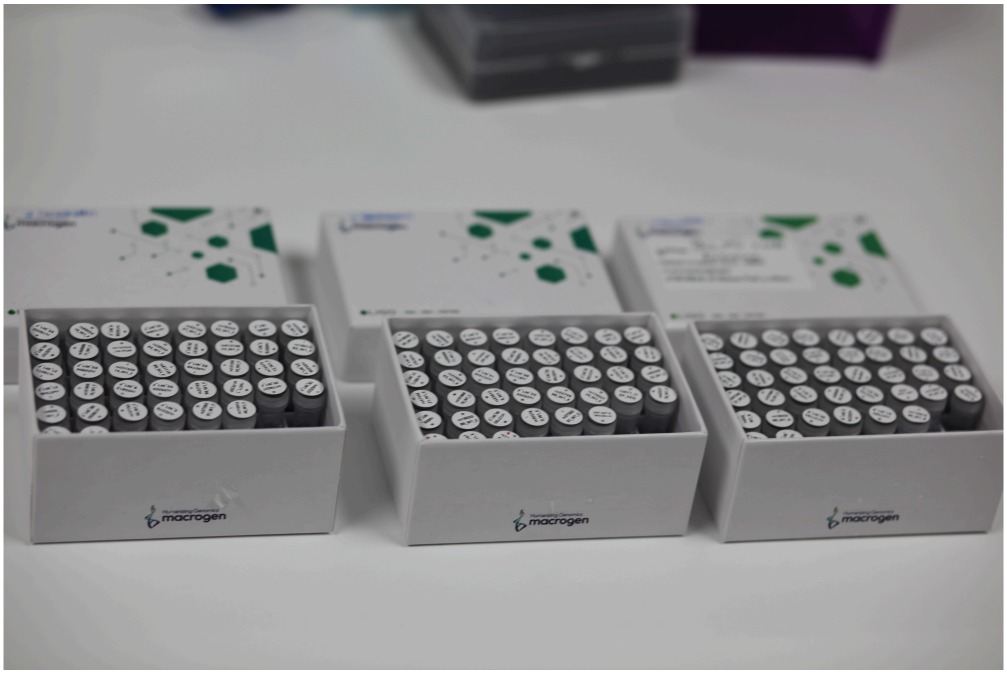

The 104 primers synthesized and used in the experiment.

14 Enter all targeted gene sequences into the program, adhering to the manufacturer's guidelines. Moreover, the primer design method followed rigorous criteria to reduce primer-dimer formation, hairpin loops, and non-specific priming, hence guaranteeing the synthesis of excellent quality primers.

15 Furthermore, assess the specificity of the synthesized primers using the BLAST (Basic Local Alignment Search Tool) software to verify optimal primer specificity. Choose only primers demonstrating the maximum specificity for the multiplex PCR based protocol developed in this study.

16 It is essential to note here that manual modifications, including base alterations and primer sequence changes, were carried out when required to attain uniformity in guanine-cytosine content (%GC) values and melting temperature (Tm) among multiplex primers, hence optimizing PCR amplification protocol.

17 Synthesize all primers by Macrogen, Inc. in Seoul, Korea and their sequences are shown in Table 1.

	A	B	C	D
	Targeted SNPs	*Forward Prim*	*Reverse Primer*	*Extended Prim*

	A	B	C	D
		er Sequence	*Sequence*	*er Sequence*
	rs10186876	ACGTTGGATGGATCTCCAGGAGAGAATGTG	ACGTTGGATGTTCCCCAGGAGCTTGTTTTC	aaacTGTGGAGGAAAGTAGA
	rs11091046	ACGTTGGATGGGATTATTCAGGCTTTAGGC	ACGTTGGATGCCTCCACTCAAGTGAAATGC	AGGAATATAATTTATAGC
	rs1137070	ACGTTGGATGCTTAAATGGTCTCGGGAAGG	ACGTTGGATGCCAGAGTCACCAAACTTACC	tGGTGACCGAGAAAGA
	rs11549465	ACGTTGGATGCTTCCAGTTAC GTTCCTTCG	ACGTTGGATGCTTTGAGGACTTGCGCTTTC	TCGATCAGTTGTCA
	rs12778366	ACGTTGGATGGCTAAGGTCCTATCTACATC	ACGTTGGATGTAAGGCTTCTAGGACTGGAG	TCTTATTTCATCTGGTCACCACT

	A	B	C	D
	rs13135092	ACGTTGGATGCCGTCACATAAACAGAACC	ACGTTGGATGTTAGCTTGAAAGGGTGTTG	ggacGGGTGTTGAATTTTA
	rs143384	ACGTTGGATGCGCTGAATGACACCAAAGAG	ACGTTGGATGCCTTTCATGGTTTTTCCTGC	ACCAGAGGCACCTT
	rs17602729	ACGTTGGATGCTGACAAATGGCAGCAAAAG	ACGTTGGATGGCCACCATGATTACAGAAAG	gttaATACAGCTGAAGAGAAA
	rs1801131	ACGTTGGATGCCGAGAGGTAAAGAACGAAG	ACGTTGGATGTCTACCTGAAGAGCAAGTCC	ggGACTTCAAAGACACTT
	rs1801282	ACGTTGGATGGTTATGGGTGAAACTCTGGG	ACGTTGGATGGTTTGCAGACAGTGTATCAG	GGAGATTCTCCTATTGAC

	A	B	C	D
	rs1805065	ACGTTGGATGTCCAGGTGCCTTCTTGATCC	ACGTTGGATGCCAGCTCCATGTAGAACAG	CTTGATCCCGTACA
	rs1805086	ACGTTGGATGGACGGGTCTCAAATATATCC	ACGTTGGATGGTGGATGGAAAACCCAAATG	agACAATACAATAAAGTAGTAA
	rs1815739	ACGTTGGATGCGATCAGTTCAAGGCAACAC	ACGTTGGATGCAGATCTTCTGGATCTCACC	gACTGCCCGAGGCTGAC
	rs2070744	ACGTTGGATGACTGTAGTTTCCCTAGTCCC	ACGTTGGATGAGGTCAGCAGAGAGACTAGG	CAAGCTCTTCCCTGGC
	rs2275998	ACGTTGGATGCTACGTTATTACACCGACGC	ACGTTGGATGAAGCCTCATCTGCTAAGGTG	gcgtCCTAGCTCGTCCTAGGG
	rs2290463	ACGTTGGATGAAGGTGGA	ACGTTGGATGAGCATGAG	ccccTCCCCCCAGGT

	A	B	C	D
		GGTAAGGGCTG	GGCTCCCAACT	TGGA
	rs2439823	ACGTTGGATGTAAGTGAGTGACAGGGAAGG	ACGTTGGATGAGACTATCTCACCTTTCAGC	ACCTTTCAGCTCTCTA
	rs2854464	ACGTTGGATGGAATCCTGGTGGAAGTCTTG	ACGTTGGATGGATGCTGCTGAGGATGATG	GGCTGGCTCATTTCCCA
	rs2920503	ACGTTGGATGCTCAGTGGGTTTTTCGAAC	ACGTTGGATGTTCTGGGACATTTTAATGG	cTTAATCTTGATTATATTCAA
	rs303760	ACGTTGGATGTCTTCGATGAGGCCAACAAG	ACGTTGGATGGACAGGACAGGAGGGGACG	ggtaGCGCGGGACCGGCCTTGGC
	rs3213537	ACGTTGGATGAAGAGGCCAGAGAGTATGAG	ACGTTGGATGGGGAGGAGAGAGCTTTTAAG	ATGAGGGTCATGGT
	rs3758391	ACGTTG	ACGTTG	CAAACA

	A	B	C	D
		GAT GG CCA TAA CAA ACA CTG GCT C	GAT GG CAC ACT GTG ACT CCA TAT C	CTG GCT CTA GAT CTA CCA
	rs4074992	ACG TTG GAT GTC ATC GTC ATG ACC ACC AG	ACG TTG GAT GTT GG GCA GCC GAA GAT GCA C	ttgg GC GGT GCC AGT GCT C
	rs41274853	ACG TTG GAT GTC TTT GGT GG GTG GGT TAG	ACG TTG GAT GGA CAC AAT TTG TGG AGA CCC	cGC CGC CCC CCA GCC CTG
	rs4253778	ACG TTG GAT GG GTG GAA CAC TTG AAG CTT G	ACG TTG GAT GTT CTG GAG ATC ACA ACC ACC	ttttA AGC TTG ATA TCT AGT TT
	rs4734621	ACG TTG GAT GAC CAC CAC ACA CAG CTA ATC	ACG TTG GAT GCA GCC TGG GCA TTA TAG TG	TGT TTA TTT TTT TGT AGA AA
	rs55743914	ACG TTG GAT GAC TTT CTT TCT	ACG TTG GAT GGT CAA GG CTC	gTT CTT ACT CTG CAT ACA G

	A	B	C	D
		TAC TCT GC	GTA AAG TCA G	
	rs558129	ACG TTG GAT GGA TCT TAA AAT GCA GAG GTC	ACG TTG GAT GAT TCC CCA GTG TGT CTT TTG	ctta CCT AAA TTC AAT CAC AA
	rs56068671	ACG TTG GAT GCA GAA AGA GAT ATA CTG TGG	ACG TTG GAT GTT CTT GGT GAA TTG ACT CC	GGA AAA TTA AGC TAA G
	rs671	ACG TTG GAT GAG GTC CCA CAC TCA CAG TTT	ACG TTG GAT GCC TTT GGT GG CTA CAA GAT G	CCC ACA CTC ACA GTT TTC ACT T
	rs680	ACG TTG GAT GAA ATT CCC GTG AGA AGG GAG	ACG TTG GAT GTC CCT GAA CCA GCA AAG AG	AAG AGA AAA GAA GG
	rs6905419	ACG TTG GAT GTA TCG CCC AGG CTG GAA TG	ACG TTG GAT GAG GCT GAA GCA GGA GAA TG	ATC TCG GCT CAC TG
	rs699	ACG TTG	ACG TTG	gGG AAG

	A	B	C	D
		GATGGTGGACGTAGGTGTTGAAAG	GATGCTGTGACAGGATGGAAGAC	ACTGGCTGCTCCCTGA
	rs699785	ACGTTGGATGGAGTGCAATGGTGCAATCTC	ACGTTGGATGTAATCCTGGCTACTTGGGAG	cttCTCATCGCAAACTCC
	rs7247312	ACGTTGGATGAGATCACACCACTGCACTC	ACGTTGGATGCAAAGTGGGTCAACTGGAAC	ctccTTTTCTGTCTCTTTTT
	rs7832552	ACGTTGGATGGCCTTGACCTCAAAGGAATG	ACGTTGGATGACAACAAGAGTCAAGCACCC	GATAGTGTGAGGTA
	rs8111989	ACGTTGGATGAGCTTTCTAGGAGAAATGGG	ACGTTGGATGCTGACTTCATCCCTCTGTAG	aagTTCTCAAGAACCTGCC
	rs9320823	ACGTTGGATGCTTGGGTGGCTTCA	ACGTTGGATGAGAAATGAGGGAG	ttGCCTTTTTCTGTCATGAA

	A	B	C	D
		AAC TAC	ATA AGG G	

Table 1. The PCR primers (forward, reverse and extension) sequence of 38 SNPs linked to athletic success in power-related sports.

Step 3: The initial PCR

9m

18 The initial 5 µL PCR reaction mix contained 0.50 µL PCR Buffer, 0.40 µL $MgCl_2$, 0.10 µL dNTPs and 0.20 PCR Enzyme (Agena Bioscience, California, United States), 1 µL of primer mixture yielding a final concentration of [M] 500 nanomolar (nM) for each forward and reverse primer, 2.0 µL of DNA template, and 0.80 µL of DNase-free distilled water.

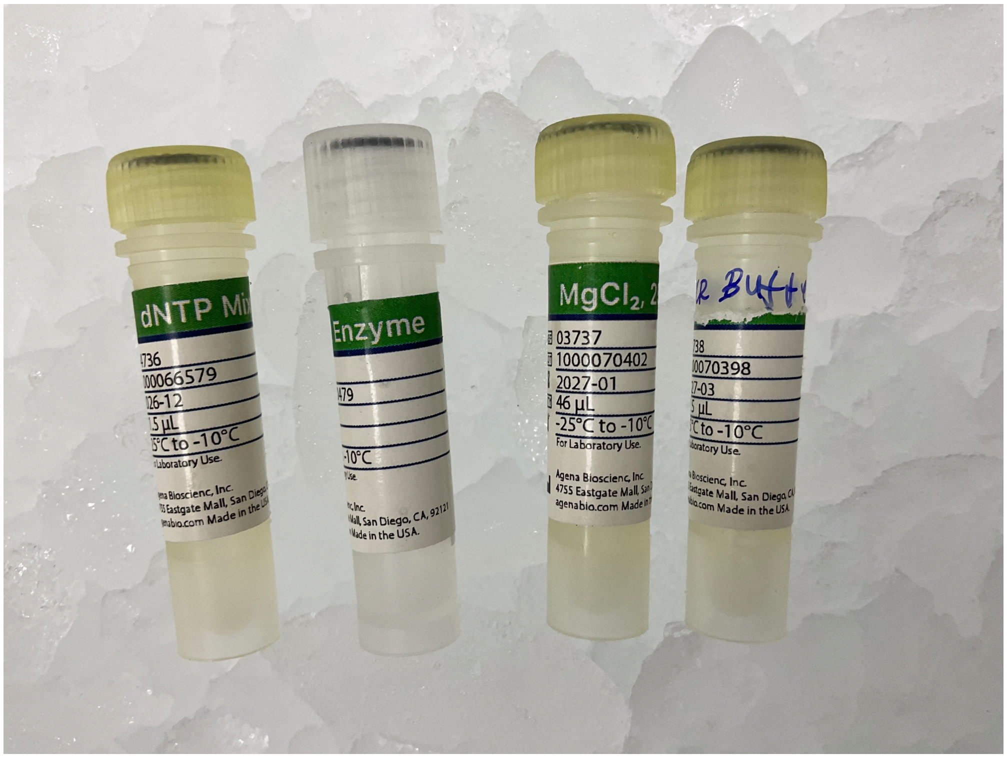

The dNTPs, polymerase and other reagents used for the first reaction.

19 Perform the multiplex PCR amplification assay with a MiniAmp™ Thermal Cycler (Thermo Fisher Scientific, Waltham, MA, USA) in our laboratory at Faculty of Science, Mahidol University.

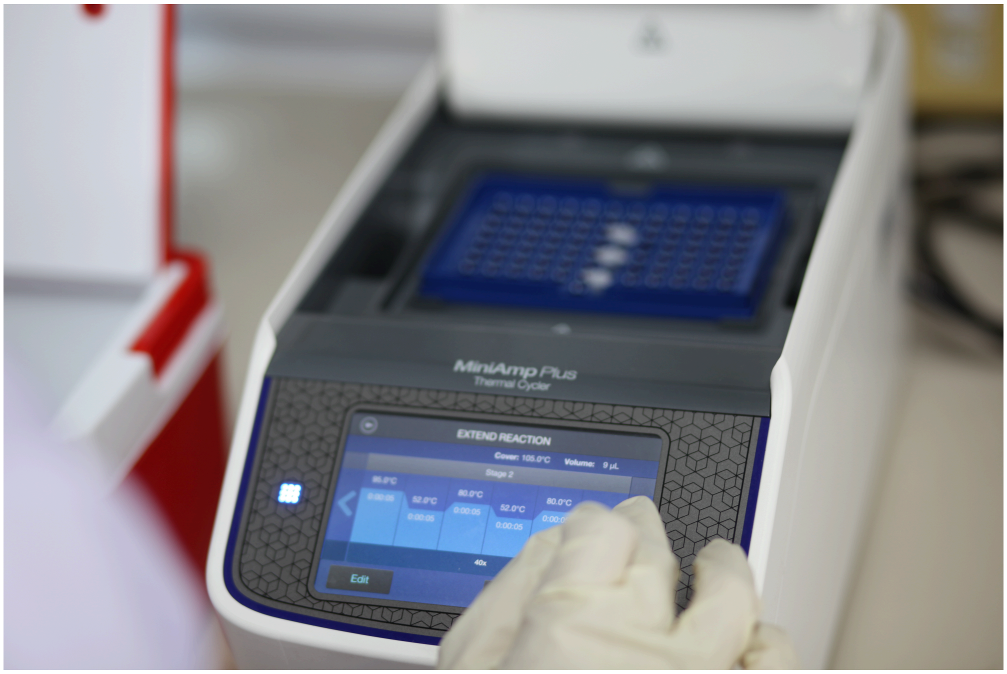

The MiniAmp™ Thermal Cycler (Thermo Fisher Scientific, Waltham, MA, USA) used for the experiment.

20 The PCR involved a denaturation at 95 °C for 00:02:00 , followed by 45 cycles of denaturation at 95 °C for 00:00:30 , annealing at 55 °C for 00:00:30 , extension at 72 °C for 00:01:00 , and a final extension at 72 °C for 00:05:00 as shown in Table 2.

9m

	A	B	C	D	E
	**PCR Reaction**	**Volume reaction**	**Temperature**	**Time**	**Cycles**
	Stage 1	5 µl	95 °C	2 minutes	1
	Stage 2		95 °C	30 seconds	45
			56 °C	30 seconds	
			72 °C	1 minutes	
	Stage 3		72 °C	5 minutes	1

A	B	C	D	E
		4 °C	Hold	

Table 2. The initial multiplex PCR conditions and reaction volume.

21 Finally terminate the initial PCR reaction by reducing the temperature at 4 °C to end the amplification process.

Step 4: The SAP reaction

45m

22 After the initial PCR reaction, remove the unincorporated dNTPs using shrimp alkaline phosphatase (SAP) (Agena Bioscience, California, United States).

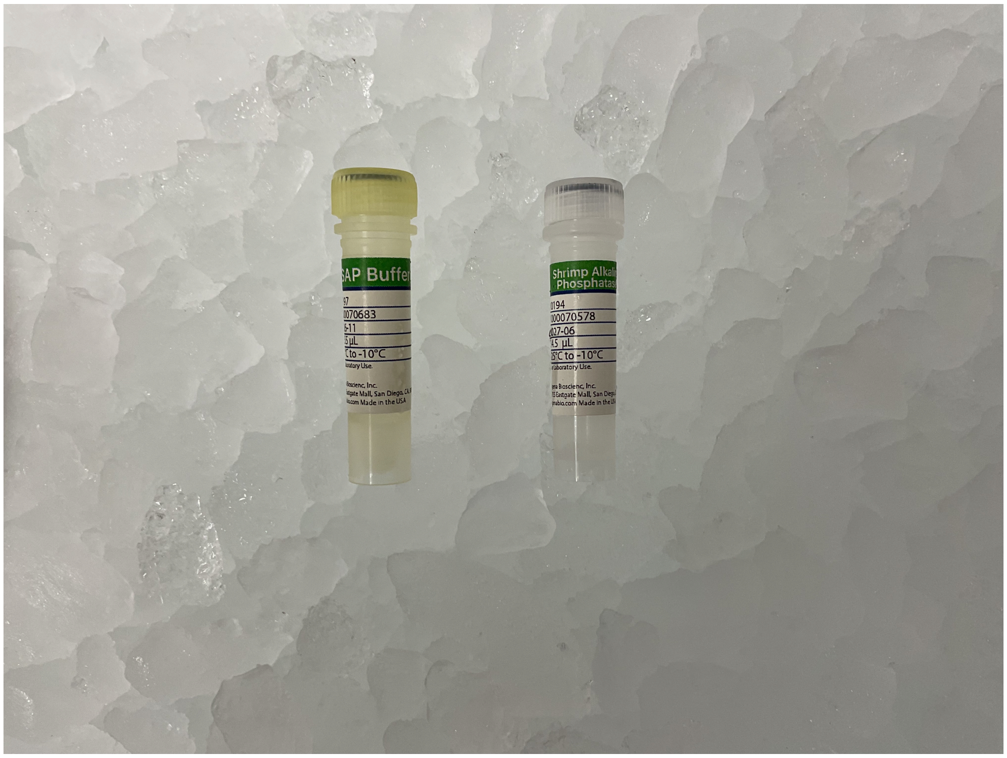

The reagents used for the SAP reaction.

23 The SAP reaction mix contained 0.17 µL SAP enzyme, 0.30 µL SAP Buffer and 1.53 µL of DNase-free distilled water.

24 Perform the dephosphorylation reaction with a MiniAmp™ Thermal Cycler (Thermo Fisher Scientific, Waltham, MA, USA) in our laboratory at Faculty of Science, Mahidol University 45m

and involved an initial stage at 37 °C for 00:40:00 , followed by inactivation at 85 °C for 00:05:00 as shown in Table 3.

	A	B	C	D	E
	**SAP Reaction**	**Volume reaction**	**Temperature**	**Time**	**Cycles**
	Stage 1	7 µl	37 °C	40 minutes	1
	Stage 2		85 °C	5 minutes	
	Stage 3		4 °C	Hold	

Table 3. The SAP reaction conditions and volume.

Step 5: The SBE reaction

3m 45s

25 Subsequent to the SAP reaction, perform the single-base extension (SBE) reaction using a MiniAmp™ Thermal Cycler (Thermo Fisher Scientific, Waltham, MA, USA) in our laboratory at Faculty of Science, Mahidol University. with a mixture of extension reaction mix synthesized in accordance with the iPLEX® Pro and Gold Reagents User Guide **[3, 4]**.

26 The extension PCR reaction contained 2 µL PCR reaction mix, 0.2 µL iPLEX® Pro Buffer, 0.2 µL iPLEX® Pro Termination Mix, 0.04 µL iPLEX® Pro Enzyme, 0.94 µL Extension Primer and 0.62 of DNase-free distilled water.

27 The next day, dispense the SBE products onto the SpectroCHIP via a nano-dispenser and then load into the mass spectrometer (MS) for the determination of the molecular mass of the SBE products (Agena Bioscience, California, United States).

28 Briefly, the SBE reaction begun with an initial step at 95 °C for 00:00:30 , continued by 40 cycles including denaturation at 95 °C for 00:00:05 , and 5 cycles of an annealing phase at 52 °C for 00:00:05 and a denaturation phase at 80 °C for 00:00:05 . Conduct the final extension reaction at 72 °C for 00:03:00 , followed by a hold at 4 °C Overnight as shown in Table 4. 3m 45s

	A	B	C	D	E
	**Extend Reaction**	**Volume reaction**	**Temperatur e**	**Time**	**Cycles**
	Stage 1	9 µl	95 °C	30 seconds	1
	Stage 2		95 °C	5 seconds	40 cycles
			52 °C	5 seconds	
			80 °C	5 seconds	
			52 °C	5 seconds	
			80 °C	5 seconds	
			52 °C	5 seconds	
			80 °C	5 seconds	
			52 °C	5 seconds	
			80 °C	5 seconds	
			52 °C	5 seconds	
			80 °C	5 seconds	
	Stage 3		72 °C	3 minutes	1
			4 °C	Hold	

Table 4. The SBE reaction conditions and volume.

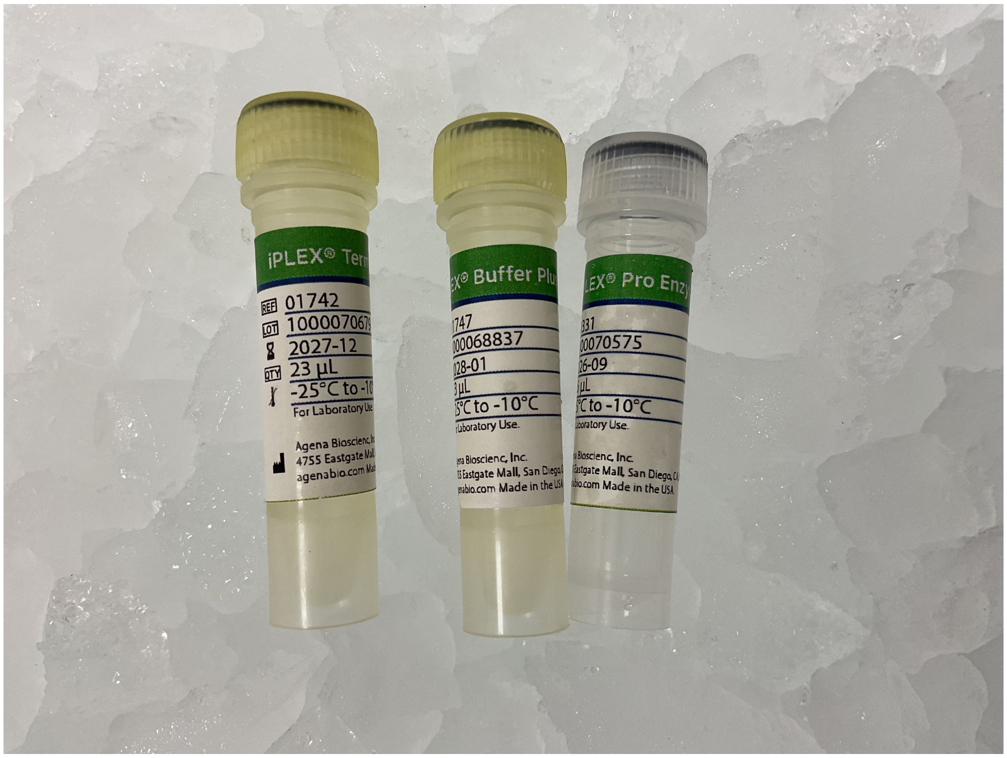

The reagents used for the iPLEX reaction.

29 Finally terminate this reaction by reducing the temperature at 4 °C to end the amplification process.

Step 6: The MS

30 The next day, dispense the SBE products onto the SpectroCHIP via a nanodispenser and load them into the mass spectrometer (MS) to determine their molecular mass.

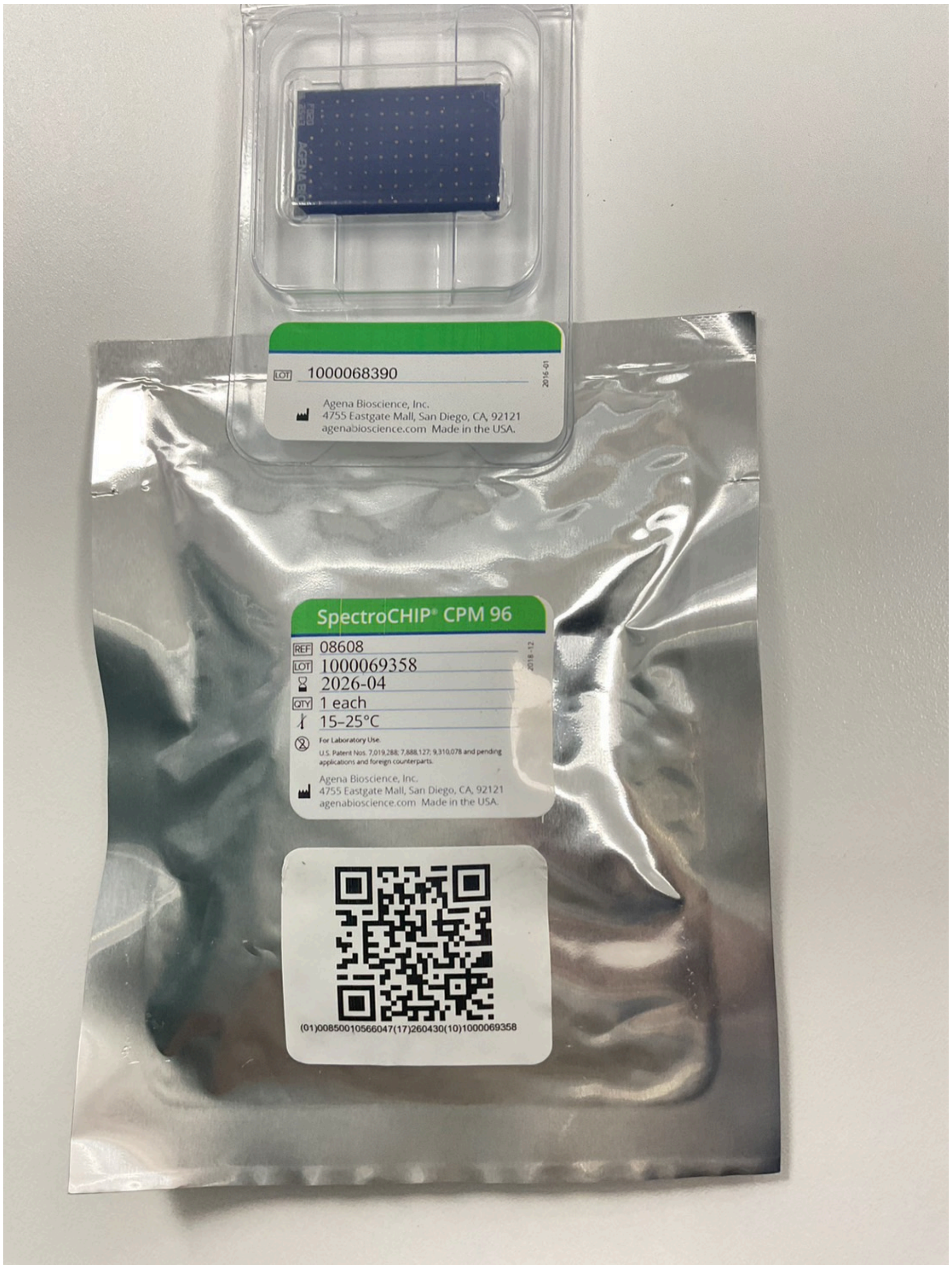

The SPECTROchip used for the experiment.

31 Evaluate and interpret the molecular mass and nucleotide-related data for individual SBE products obtained from the MS using MassARRAY® Typer Viewer v4.0 software (Agena Bioscience, California, United States).

Protocol references

1. Htet S, Zannah M, Moe TH, Wongveerakul P, Charoenpanich N, Saengsirisuwan V, et al. The speed-gene study: methods, study design and preliminary results. BMC Res Notes. 2023;16: 345. doi:10.1186/s13104-023-06617-3

2. Thompson JD, Higgins DG, Gibson TJ. CLUSTAL W: improving the sensitivity of progressive multiple sequence alignment through sequence weighting, position-specific gap penalties and weight matrix choice. Nucleic Acids Res. 1994;22: 4673–4680. doi:10.1093/nar/22.22.4673

3. Millis MP. Medium-throughput SNP genotyping using mass spectrometry: multiplex SNP genotyping using the iPLEX® Gold assay. Methods Mol Biol. 2011;700: 61–76. doi:10.1007/978-1-61737-954-3_5

4. Bradić M, Costa J, Chelo IM. Genotyping with Sequenom. Methods Mol Biol. 2011;772: 193–210. doi:10.1007/978-1-61779-228-1_11
