# Supplementary material for: A 38-plex PCR MALDI-TOF MS-based assay to detect SNPs common in elite athletes
Source: PLoS One. 2025 Dec 29;20(12):e0339384. doi: 10.1371/journal.pone.0339384 (PMC12747323; doi:10.1371/journal.pone.0339384)
Supplement: S2 Table — (PDF) [file pone.0339384.s002.pdf]

Title: The PCR primers (forward and reverse), melting temperatures Tm (C^o) and guanine-cytosine content %GC of each targeted SNPs as well as the molecular masses of the extension primers and SBE products of the assay.

Targeted SNPs	*PCR Primers (forward, reverse)*		*PCR amplicon size*	*Extension Primers*	Mass of SBE products (Da)			
	Tm (C^o)	*%GC*	*Base pairs (bp)*	*Mass (Da)*	*Base*	*Mass (Da)*	*Base*	*Mass (Da)*
rs10186876	54.9	43.8	111	6264.1	A	6535.3	G	6551.3
rs11091046	59.7	28	99	7738.1	C	8025.3	A	8065.2
rs1137070	56.3	53.3	100	4980.3	C	5227.4	T	5307.4
rs11549465	51.3	42.9	98	4254.8	C	4502	T	4581.9
rs12778366	64	39.1	111	6916.5	C	7163.7	T	7187.7
rs13135092	48.6	33.3	115	5914.9	G	6162	A	6242
rs143384	57.8	57.1	93	4233.8	G	4481	A	4560.9
rs17602729	54	35.3	128	6512.3	G	6759.5	A	6839.4
rs1801131	52.3	37.5	125	5508.6	G	5795.8	T	5835.7
rs1801282	55.6	44.4	95	5490.6	C	5737.8	G	5777.8
rs1805065	52	50	94	4199.8	C	4446.9	T	4526.8
rs1805086	51.3	20	119	6793.5	T	7064.7	C	7080.7
rs1815739	66.5	68.8	104	5197.4	C	5444.6	T	5524.5
rs2070744	61.9	62.5	115	4794.1	C	5041.3	T	5121.2
rs2275998	62.2	64.7	102	6415.2	T	6686.4	C	6702.4
rs2290463	63.8	66.7	107	5686.7	G	5933.9	C	5973.9
rs2439823	55	43.8	120	4768.1	G	5015.3	A	5095.2
rs2854464	64.6	58.8	99	5138.3	G	5385.5	A	5425.6

rs2920503	49.1	15	111	6370.2	T	6641.4	C	6657.4
rs303760	79	84.2	148	7123.6	T	7394.8	C	7410.8
rs3213537	53.7	50	95	4359.8	C	4607	T	4686.9
rs3758391	66.4	45.8	100	7266.8	C	7513.9	T	7593.8
rs4074992	65.8	73.3	120	5852.8	C	6100	T	6124
rs41274853	76.4	88.2	142	5343.5	G	5590.6	A	5670.6
rs4253778	52.1	27.8	108	6721.4	C	6968.6	G	7008.6
rs4734621	51.4	15	88	6143	C	6390.2	T	6470.1
rs55743914	55.5	38.9	128	5754.8	C	6001.9	T	6081.9
rs558129	50.8	29.4	116	6318.2	A	6589.4	G	6605.4
rs56068671	47.6	31.2	136	4963.3	G	5250.5	T	5290.4
rs671	65.8	45.5	115	6566.3	G	6813.5	A	6893.4
rs680	45.7	35.7	109	4403.9	G	4651.1	A	4731
rs6905419	55.6	57.1	107	4215.8	C	4462.9	T	4542.8
rs699	71	61.9	100	6777.4	G	7024.6	A	7104.5
rs699785	55.9	53.3	111	5355.5	A	5626.7	G	5642.7
rs7247312	48.8	25	101	5957.9	G	6205.1	A	6285
rs7832552	46.9	42.9	105	4383.9	C	4631.1	T	4711
rs8111989	57.8	50	102	5757.8	T	6029	C	6045
rs9320823	57.8	38.9	103	6065	T	6336.2	C	6352.2
