# Supplementary material for: A 38-plex PCR MALDI-TOF MS-based assay to detect SNPs common in elite athletes
Source: PLoS One. 2025 Dec 29;20(12):e0339384. doi: 10.1371/journal.pone.0339384 (PMC12747323; doi:10.1371/journal.pone.0339384)
Supplement: S4 Fig — (PDF) [file pone.0339384.s004.pdf]

Title: All 80 chromatograms for each targeted SNP

Chromatogram 1 (rs10186876 A allele)

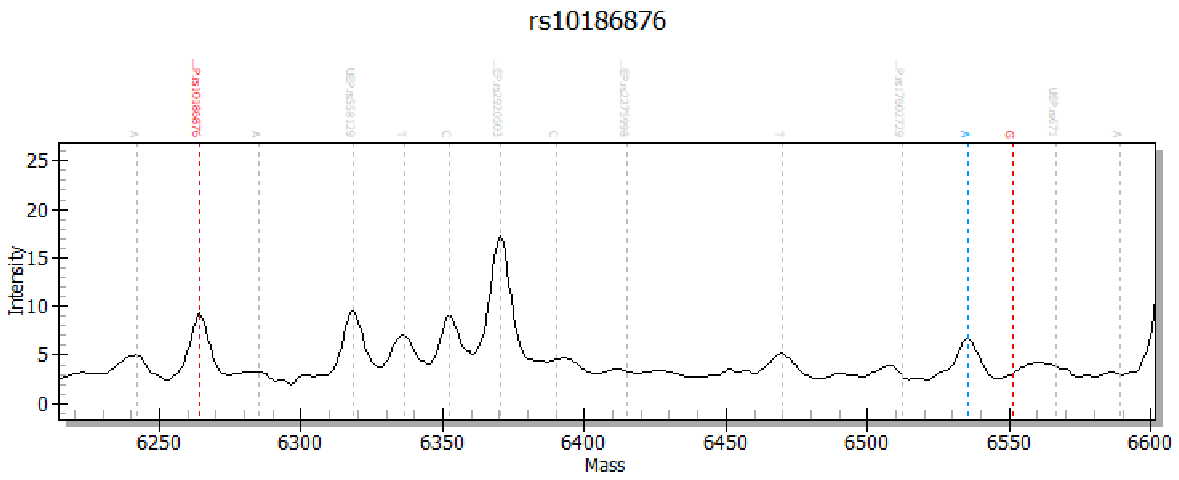

This chromatogram depicts the molecular mass of SBE products associated with the A allele of rs10186876 SNP.

Chromatogram 2 (rs11091046 A allele)

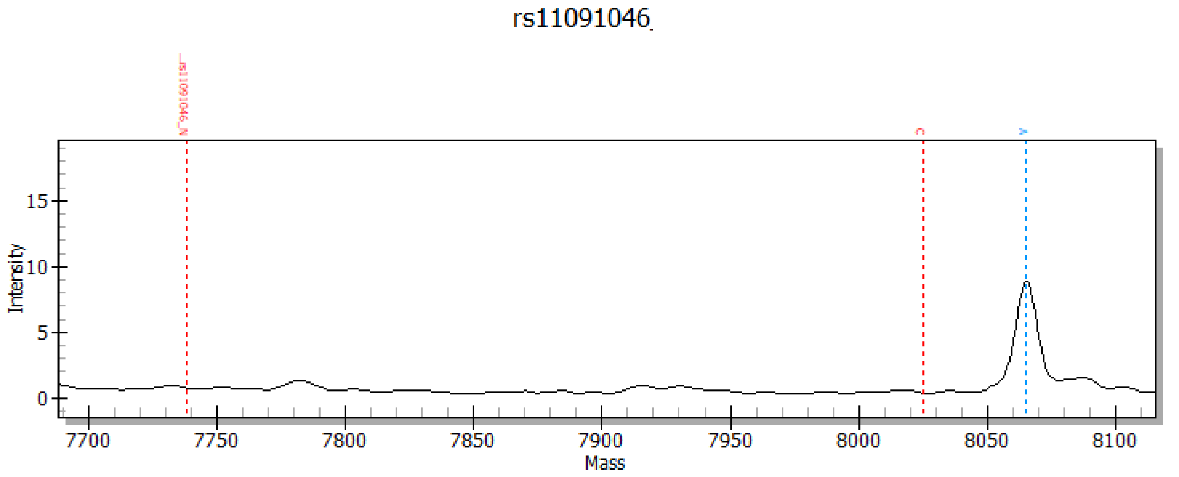

This chromatogram depicts the molecular mass SBE products associated with the detection of A allele in rs11091046 SNP.

Chromatogram 3 (rs11091046 C allele)

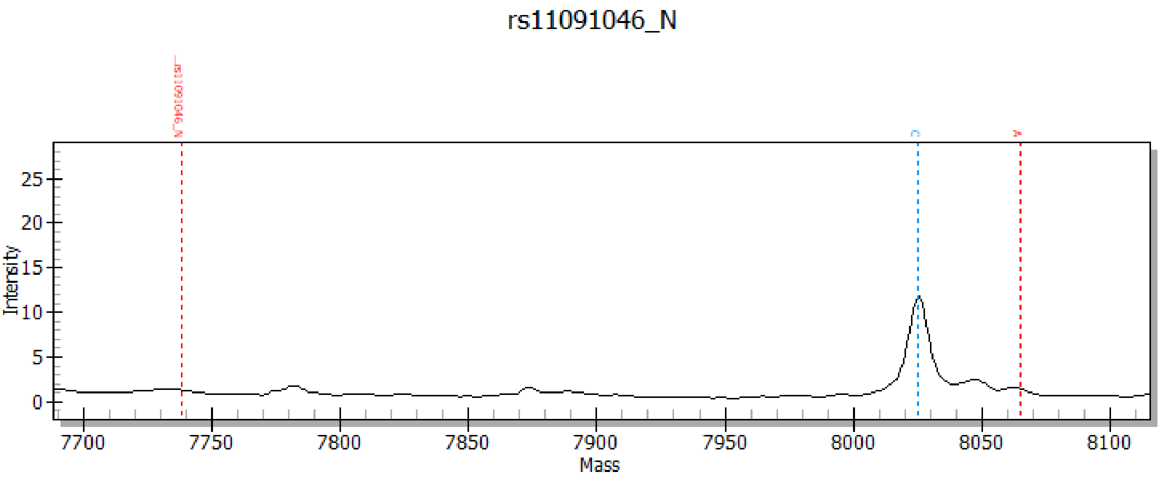

This chromatogram depicts the molecular mass SBE products associated with the detection of C allele in rs11091046 SNP.

Chromatogram 4 (rs1137070 T allele)

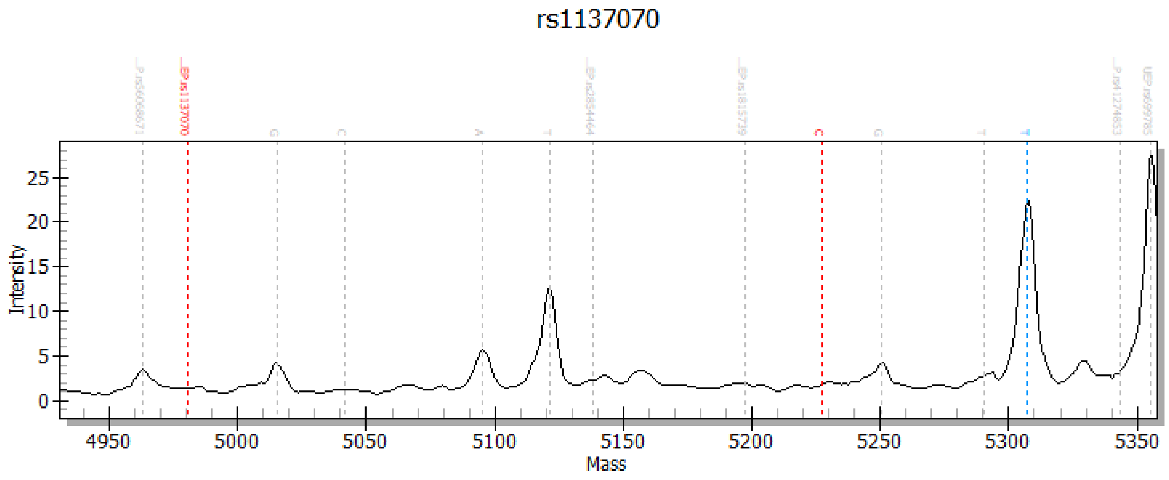

This chromatogram depicts the molecular mass SBE products associated with the detection of T allele in rs1137070 SNP.

Chromatogram 5 (rs1137070 C allele)

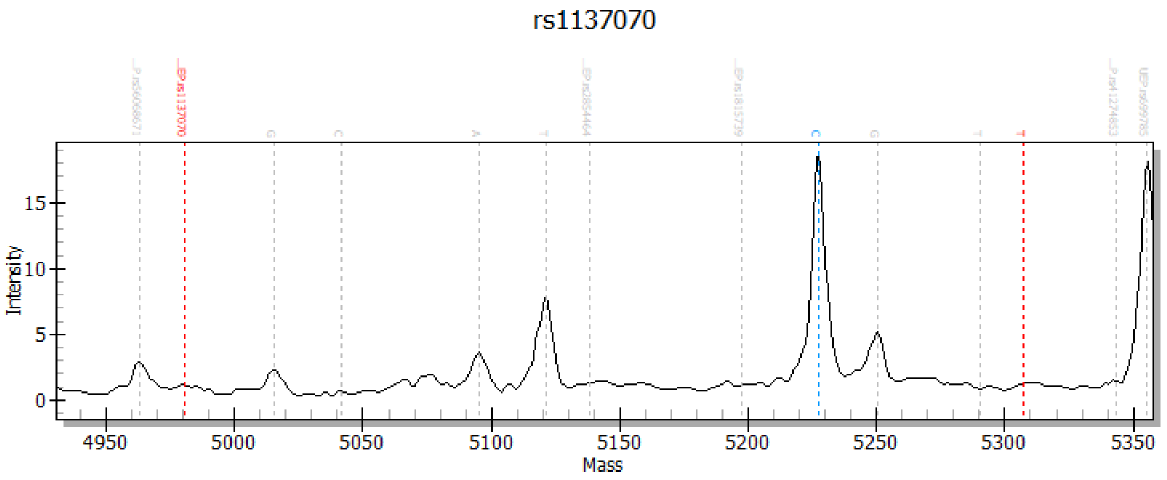

This chromatogram depicts the molecular mass SBE products associated with the detection of C allele in rs1137070 SNP.

Chromatogram 6 (rs11549465 C allele)

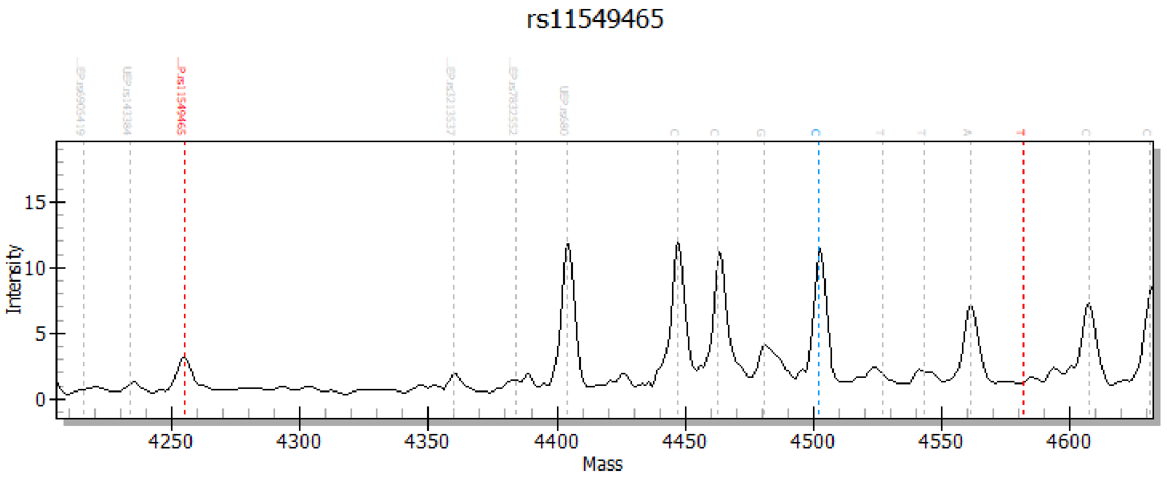

This chromatogram depicts the molecular mass SBE products associated with the detection of C allele in rs11549465 SNP.

Chromatogram 7 (rs11549465 C/T alleles)

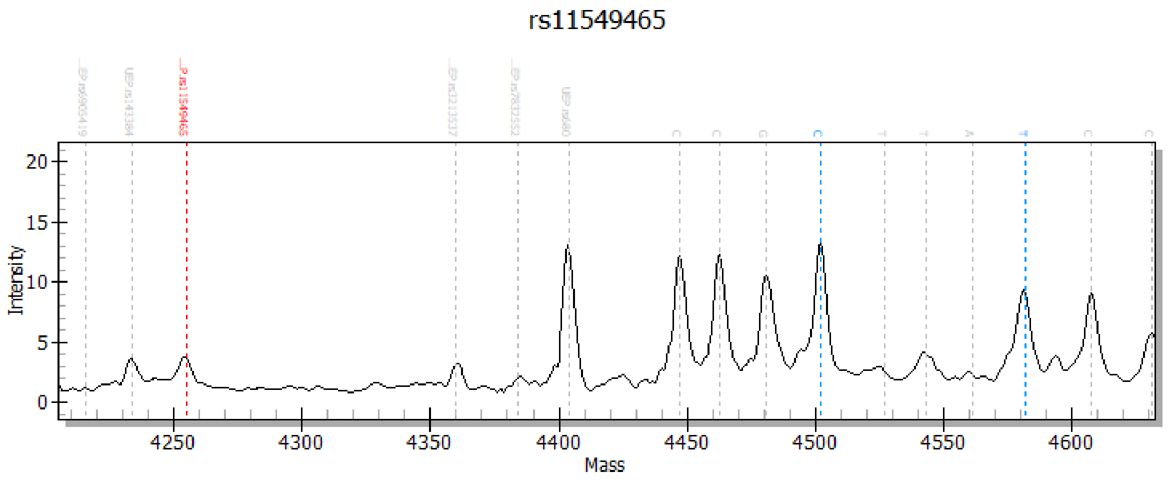

This chromatogram depicts the molecular mass SBE products associated with the detection of heterozygotes C/T alleles in rs11549465 SNP.

Chromatogram 8 (rs12778366 T allele)

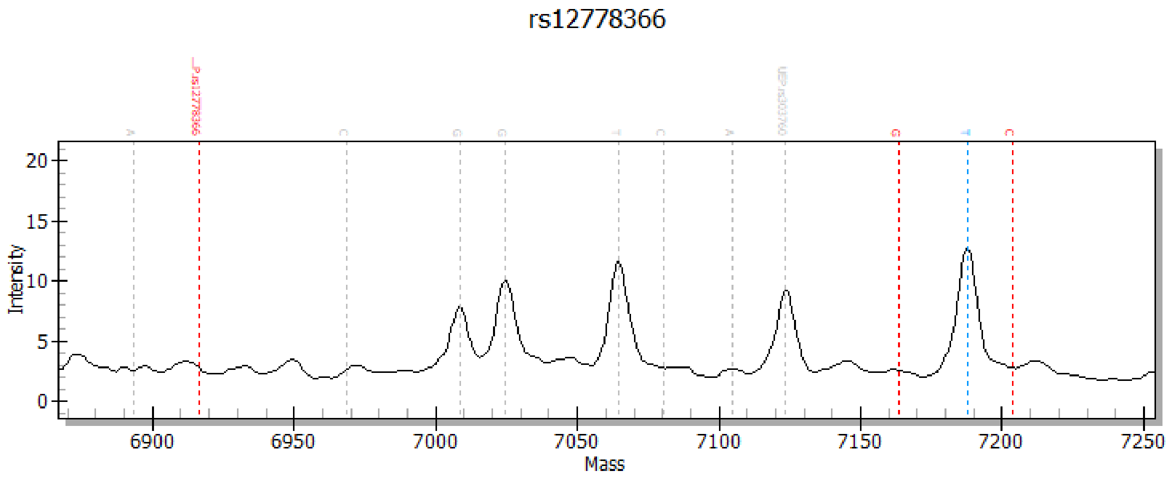

This chromatogram depicts the molecular mass SBE products associated with the detection of T allele in rs12778366 SNP.

Chromatogram 9 (rs12778366 C/T alleles)

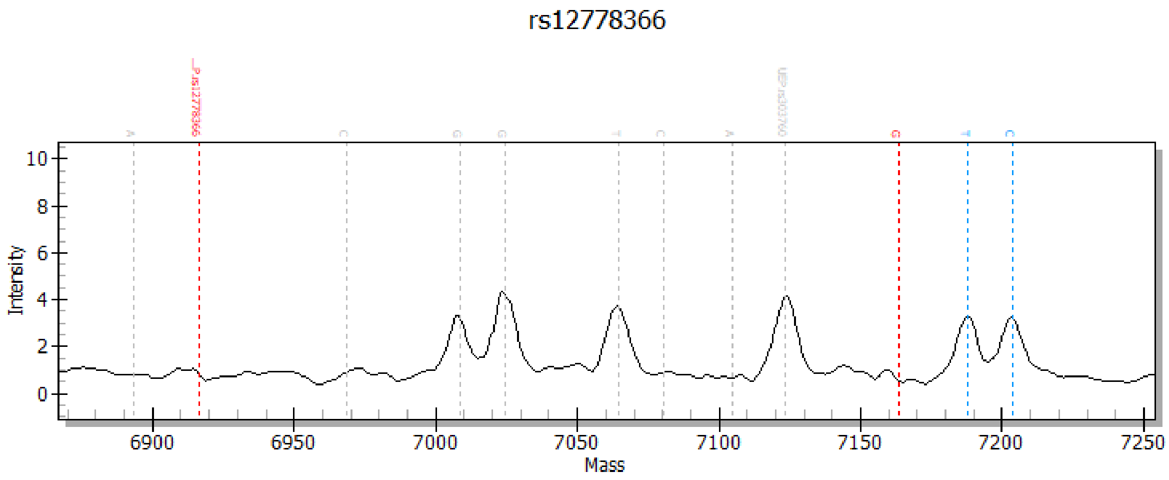

This chromatogram depicts the molecular mass SBE products associated with the detection of heterozygotes C/T alleles in rs12778366 SNP.

Chromatogram 10 (rs13135092 A allele)

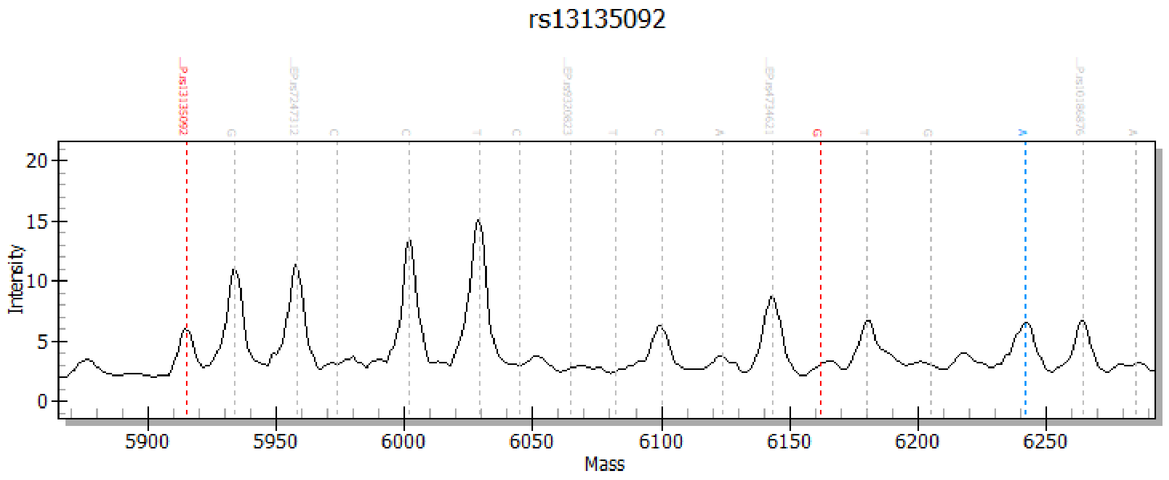

This chromatogram depicts the molecular mass SBE products associated with the detection of A alleles in rs13135092 SNP.

Chromatogram 11 (rs143384 G allele)

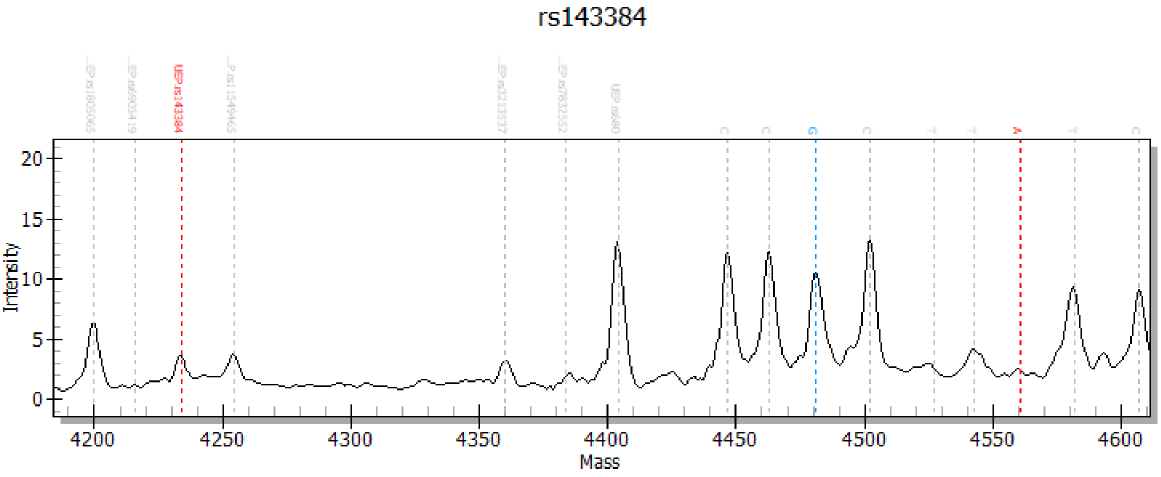

This chromatogram depicts the molecular mass SBE products associated with the detection of G alleles in rs143384 SNP.

Chromatogram 12 (rs143384 G/A alleles)

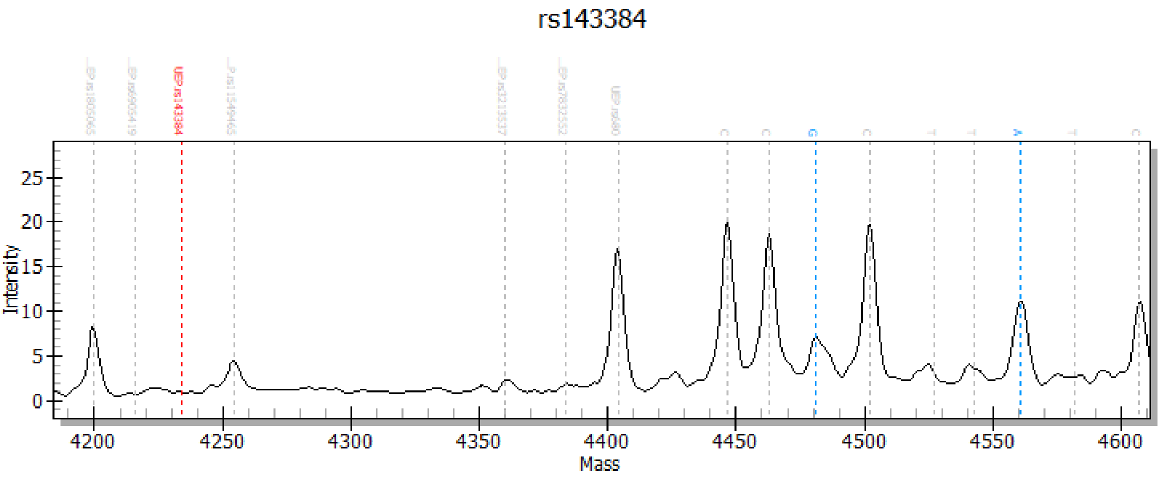

This chromatogram depicts the molecular mass SBE products associated with the detection of heterozygotes G/A alleles in rs143384 SNP.

Chromatogram 13 (rs143384 A allele)

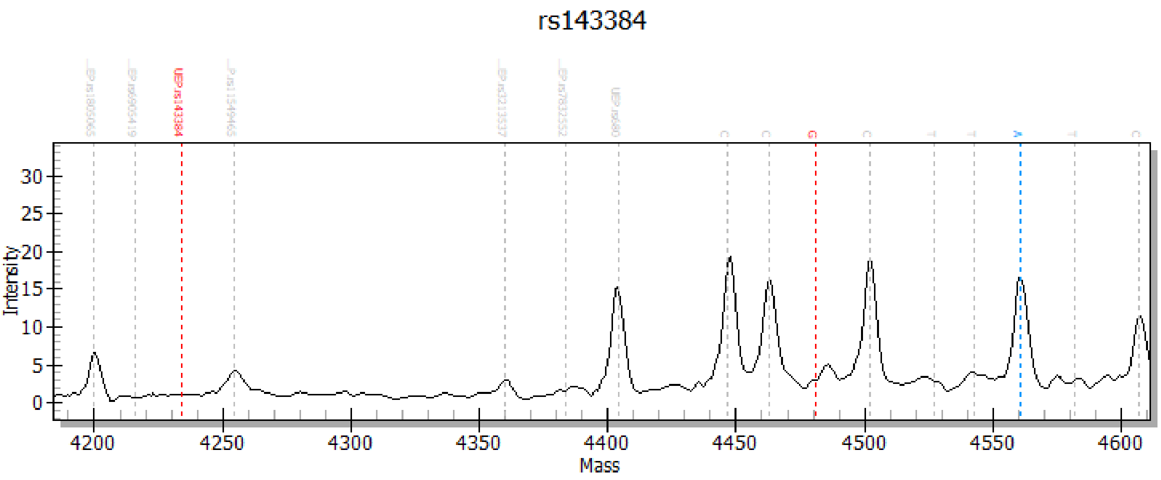

This chromatogram depicts the molecular mass SBE products associated with the detection of A allele in rs143384 SNP.

Chromatogram 14 (rs17602729 G allele)

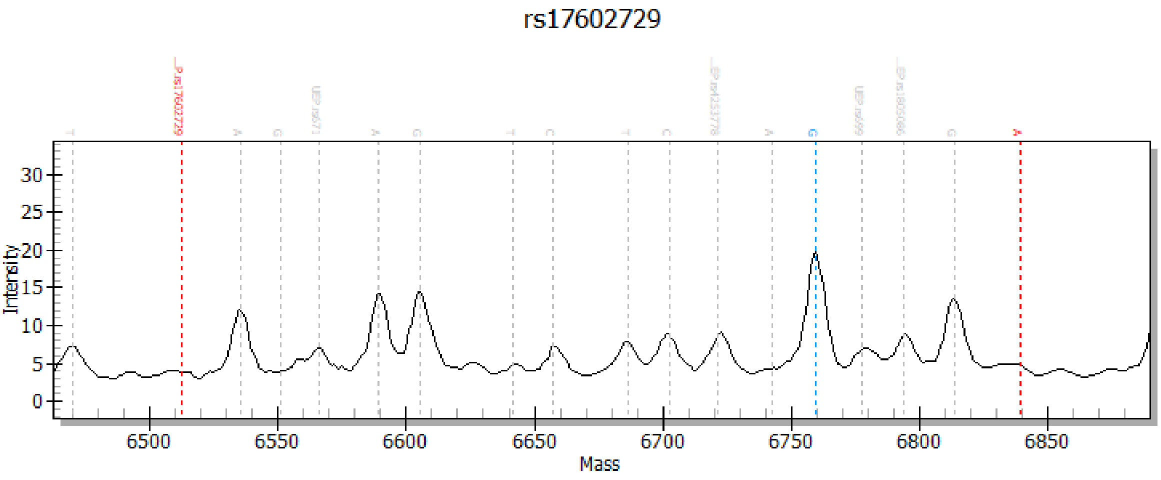

This chromatogram depicts the molecular mass SBE products associated with the detection of G allele in rs17602729 SNP.

Chromatogram 15 (rs1801131 G/T alleles)

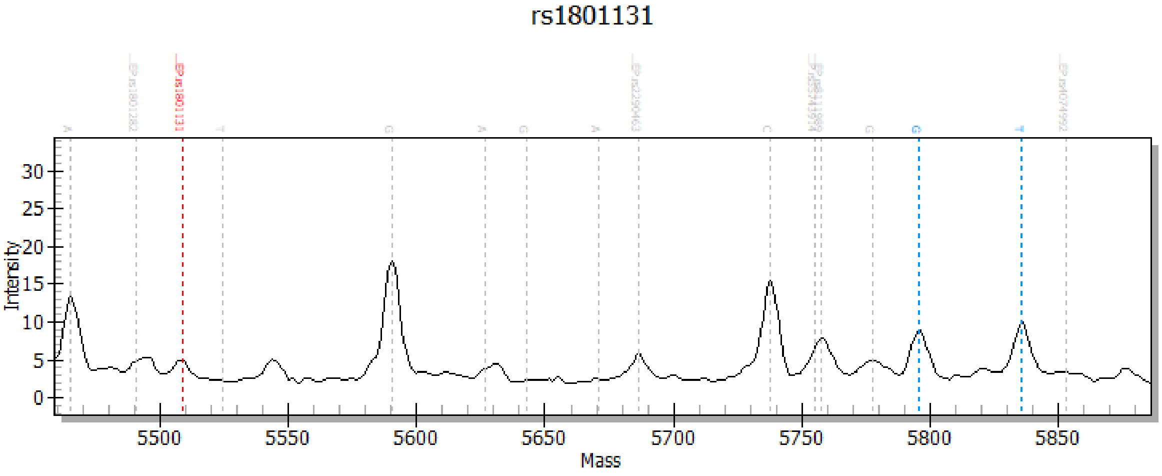

This chromatogram depicts the molecular mass SBE products associated with the detection of heterozygotes G/T alleles in rs1801131 SNP.

Chromatogram 16 (rs1801131 T allele)

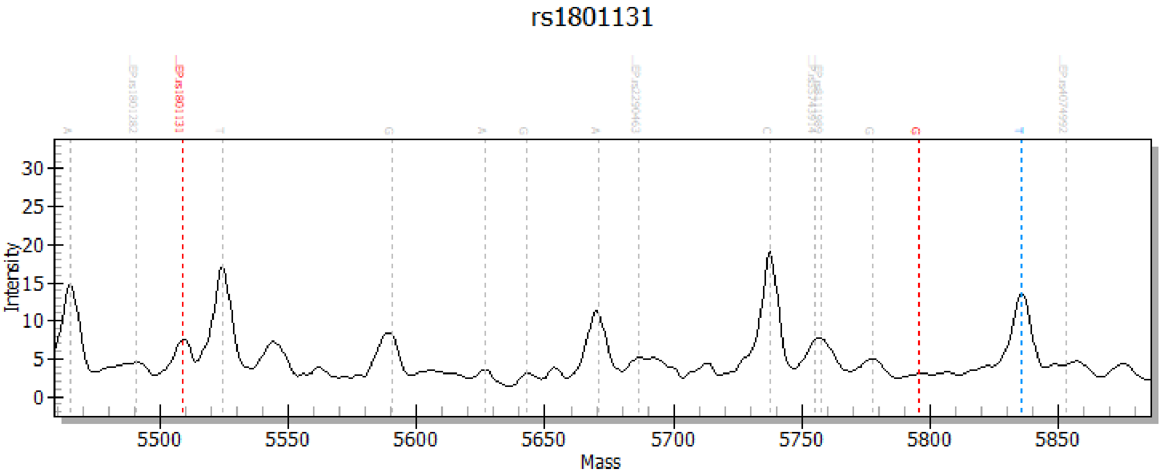

This chromatogram depicts the molecular mass SBE products associated with the detection of T allele in rs1801131 SNP.

Chromatogram 17 (rs1801282 C allele)

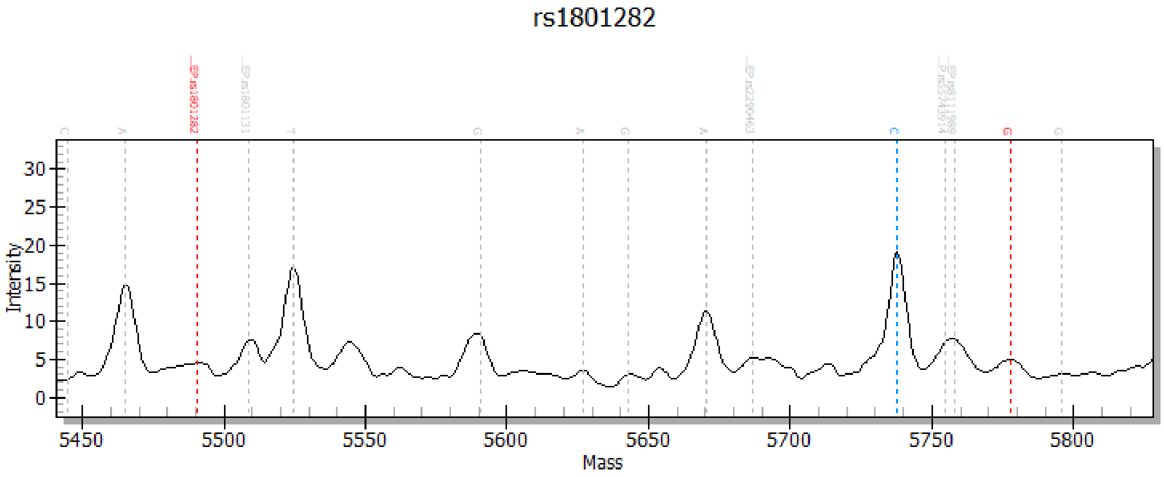

This chromatogram depicts the molecular mass SBE products associated with the detection of C alleles in rs1801282 SNP.

Chromatogram 18 (rs1801282 C/G alleles)

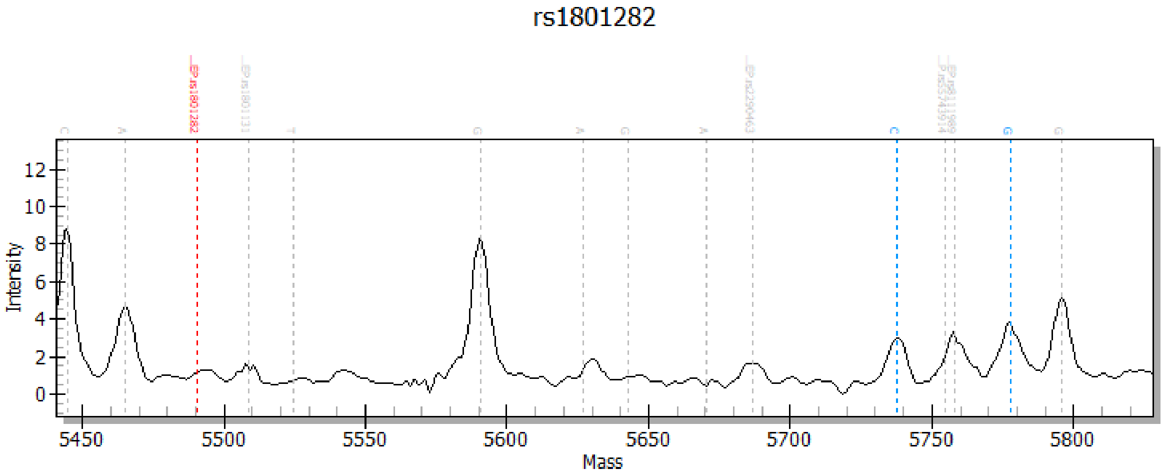

This chromatogram depicts the molecular mass SBE products associated with the detection of heterozygotes C/G alleles in rs1801282 SNP.

Chromatogram 19 (rs1805065 C allele)

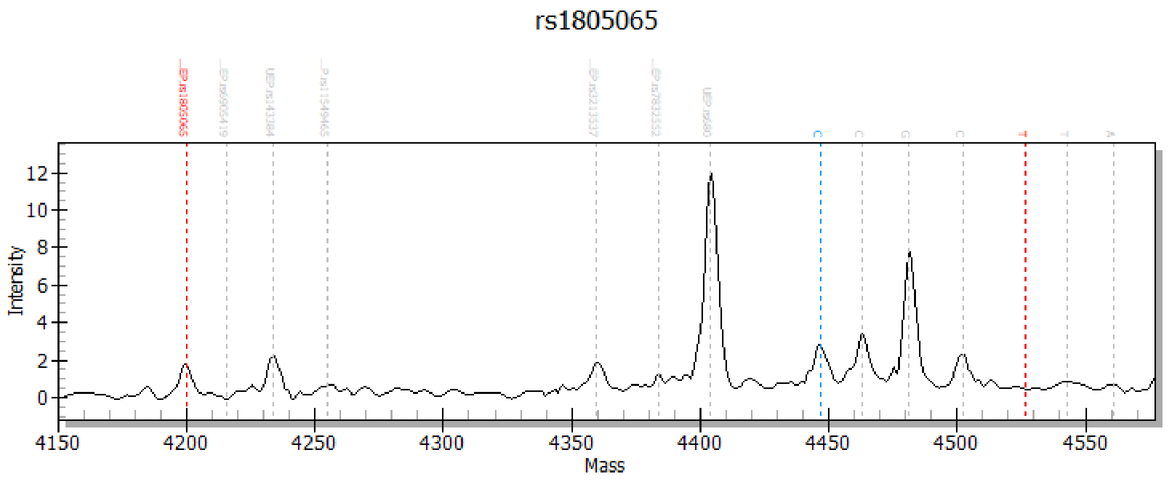

This chromatogram depicts the molecular mass SBE products associated with the detection of C allele in rs1805065 SNP.

Chromatogram 20 (rs1805086 T allele)

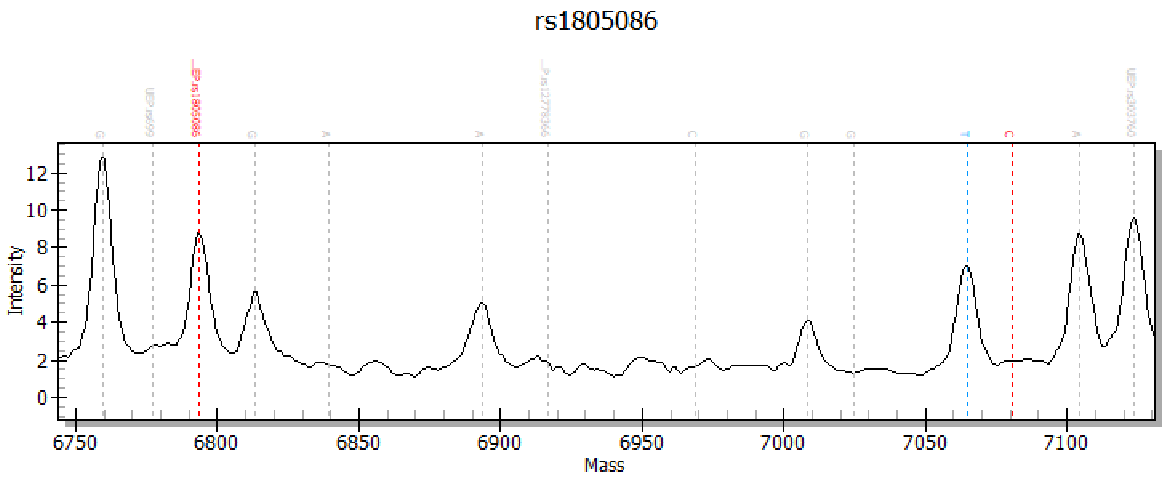

This chromatogram depicts the molecular mass SBE products associated with the detection of T allele in rs1805086 SNP.

Chromatogram 21 (rs1815739 C allele)

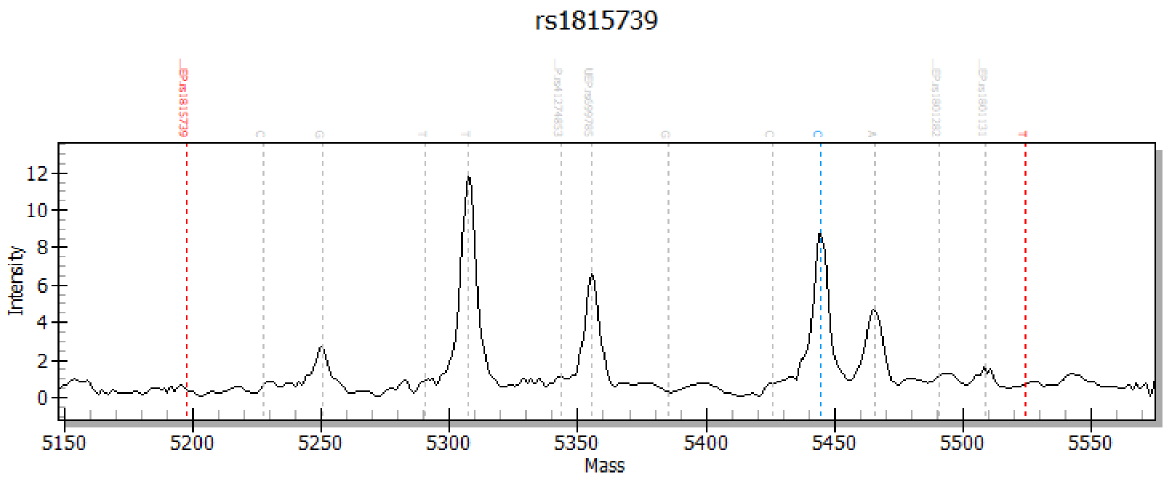

This chromatogram depicts the molecular mass SBE products associated with the detection of C allele in rs1815739 SNP.

Chromatogram 22 (rs1815739 C/T alleles)

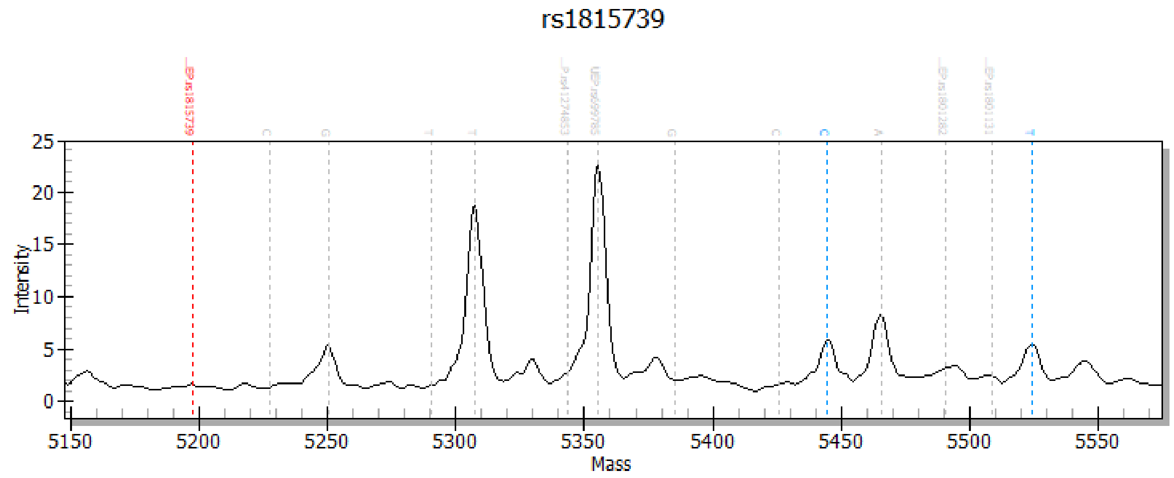

This chromatogram depicts the molecular mass SBE products associated with the detection of heterozygotes C/T alleles in rs1815739 SNP.

Chromatogram 23 (rs1815739 T allele)

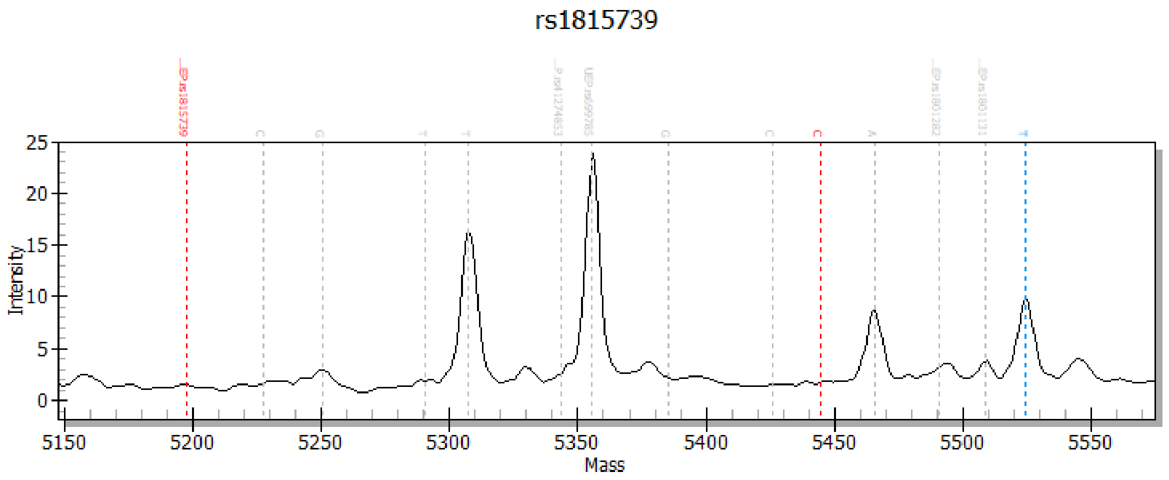

This chromatogram depicts the molecular mass SBE products associated with the detection of T alleles in rs1815739 SNP.

Chromatogram 24 (rs2070744 C allele)

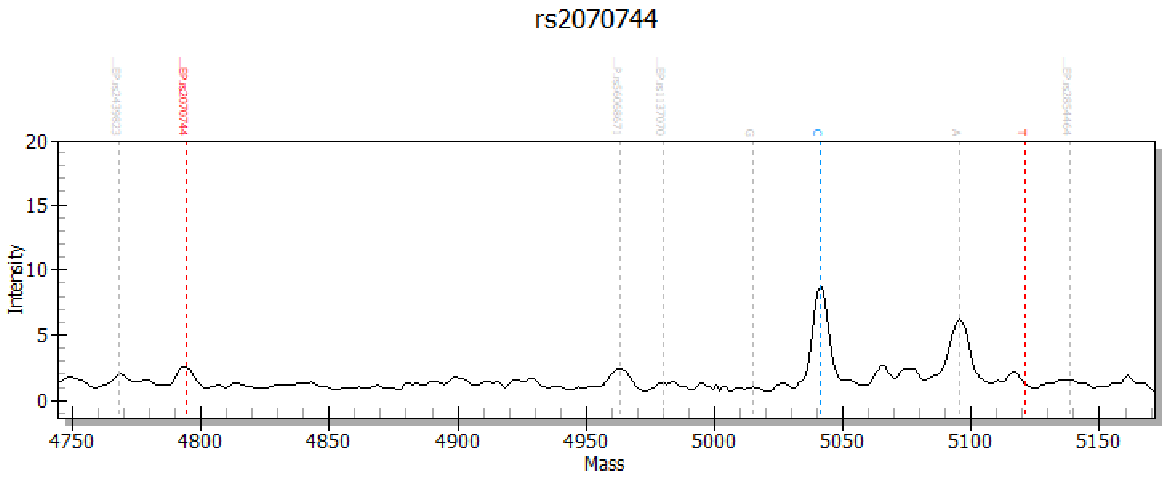

This chromatogram depicts the molecular mass SBE products associated with the detection of C alleles in rs2070744 SNP.

Chromatogram 25 (rs2070744 C/T alleles)

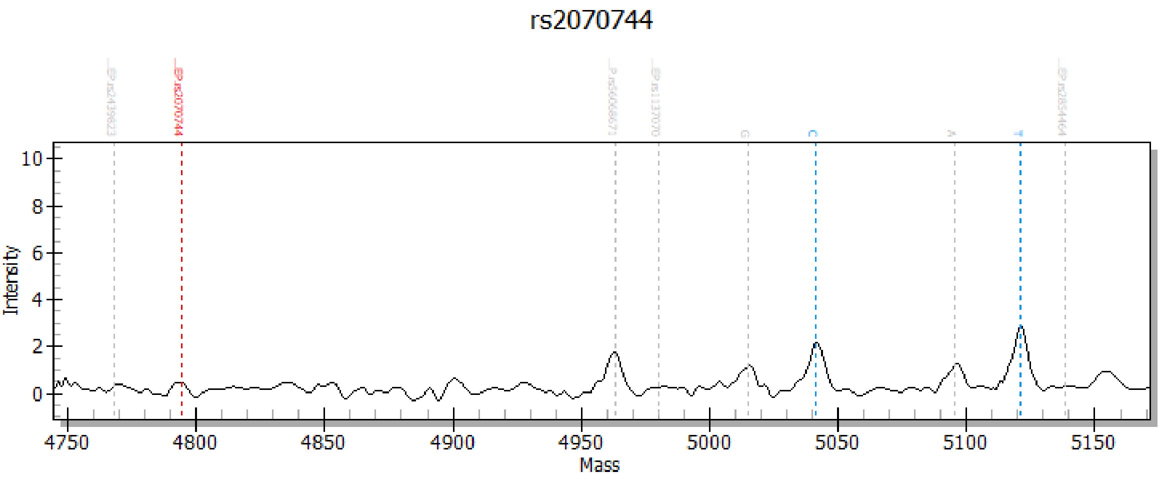

This chromatogram depicts the molecular mass SBE products associated with the detection of heterozygotes C/T alleles in rs2070744 SNP.

Chromatogram 26 (rs2070744 T allele)

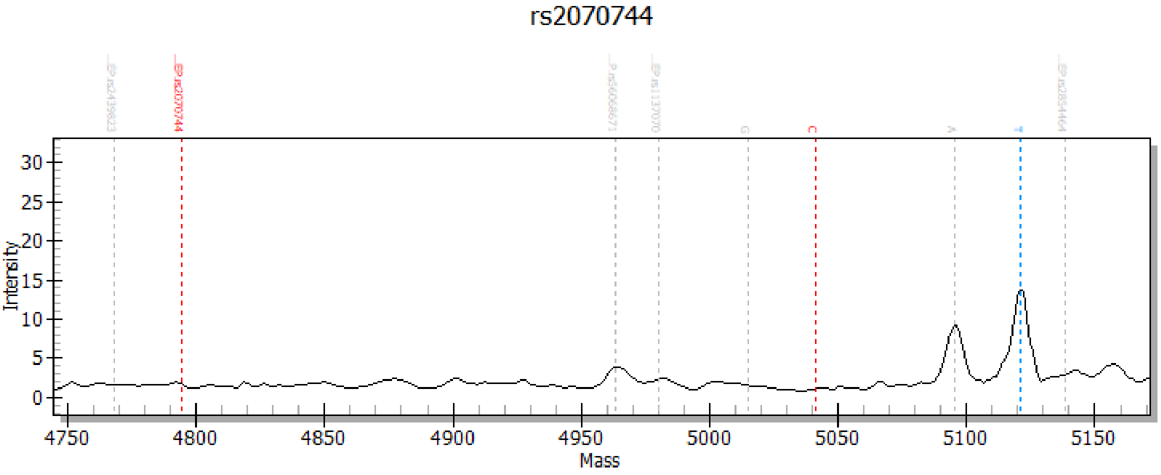

This chromatogram depicts the molecular mass SBE products associated with the detection of T alleles in rs2070744 SNP.

Chromatogram 27 (rs2275998 T allele)

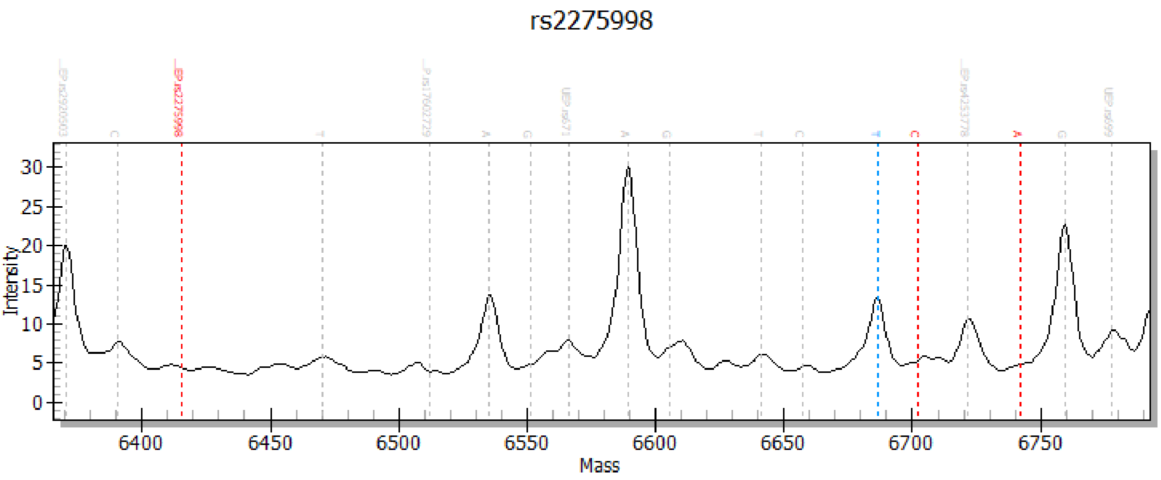

This chromatogram depicts the molecular mass SBE products associated with the detection of T alleles in rs2275998 SNP.

Chromatogram 28 (rs2275998 T/C alleles)

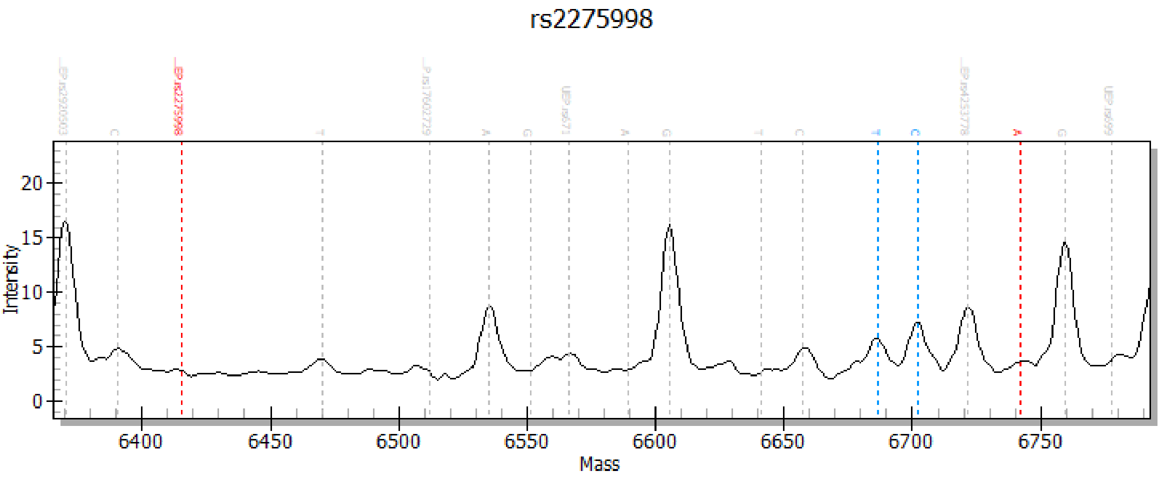

This chromatogram depicts the molecular mass SBE products associated with the detection of heterozygotes T/C alleles in rs2275998 SNP.

Chromatogram 29 (rs2290463 G allele)

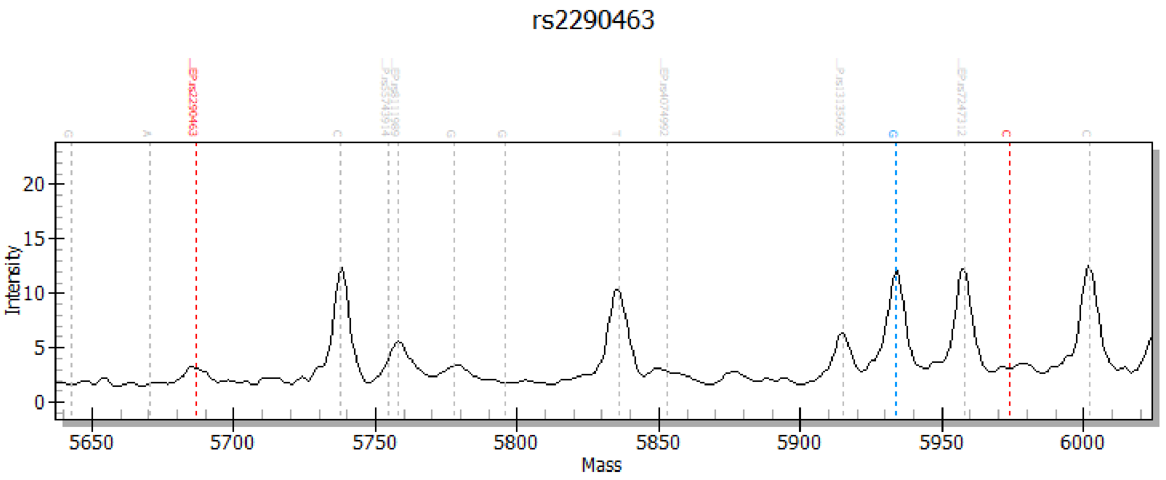

This chromatogram depicts the molecular mass SBE products associated with the detection of G alleles in rs2290463 SNP.

Chromatogram 30 (rs2290463 G/C alleles)

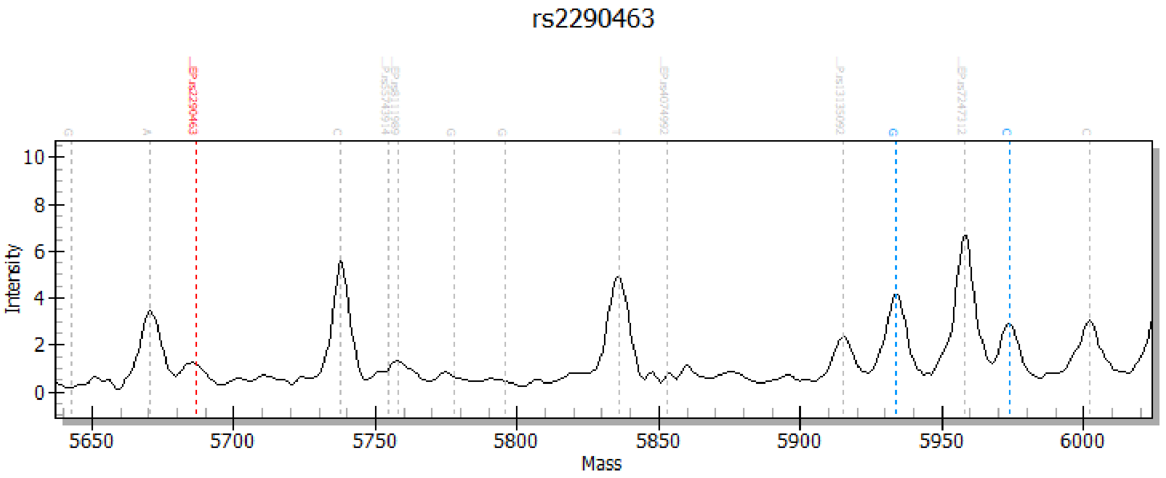

This chromatogram depicts the molecular mass SBE products associated with the detection of heterozygotes G/C alleles in rs2290463 SNP.

Chromatogram 31 (rs2439823 G allele)

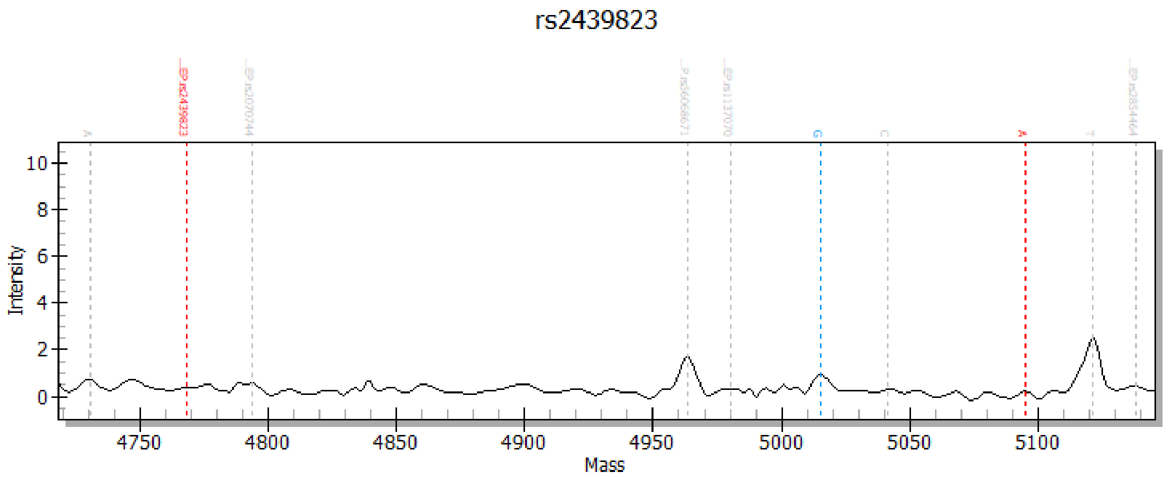

This chromatogram depicts the molecular mass SBE products associated with the detection of G alleles in rs2439823 SNP.

Chromatogram 32 (rs2439823 G/A alleles)

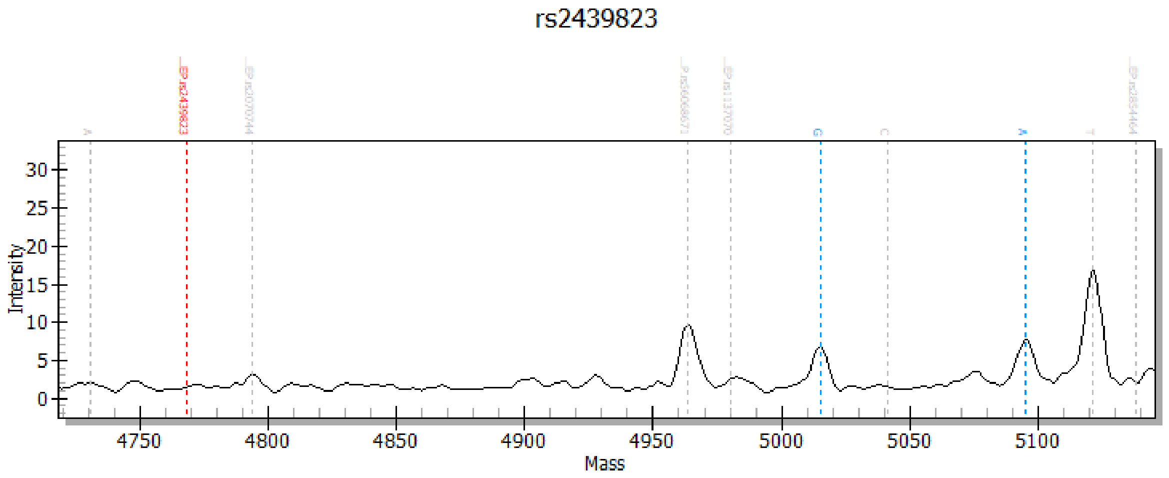

This chromatogram depicts the molecular mass SBE products associated with the detection of heterozygotes G/A alleles in rs2439823 SNP.

Chromatogram 33 (rs2439823 A allele)

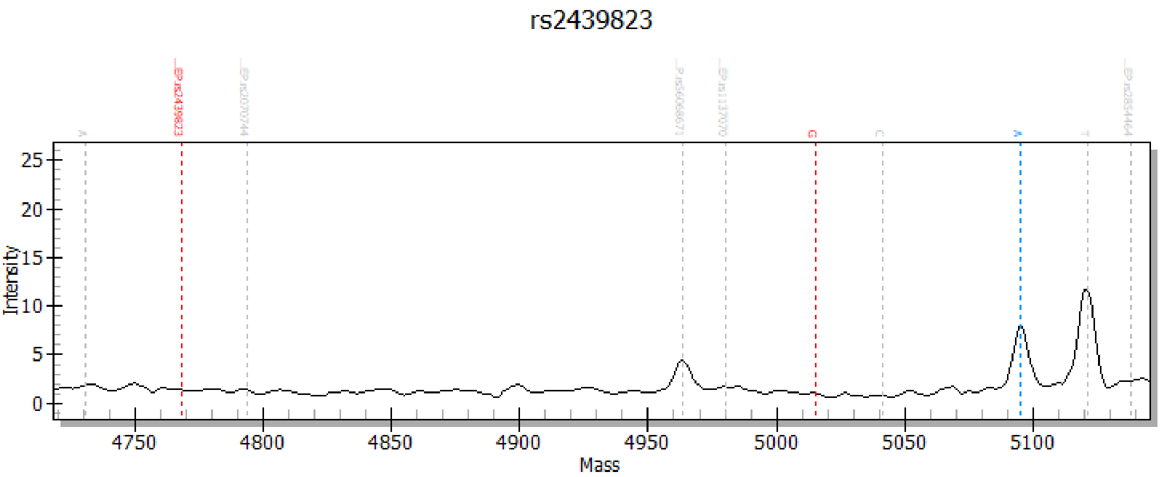

This chromatogram depicts the molecular mass SBE products associated with the detection of A allele in rs2290463 SNP.

Chromatogram 34 (rs2854464 G/A alleles)

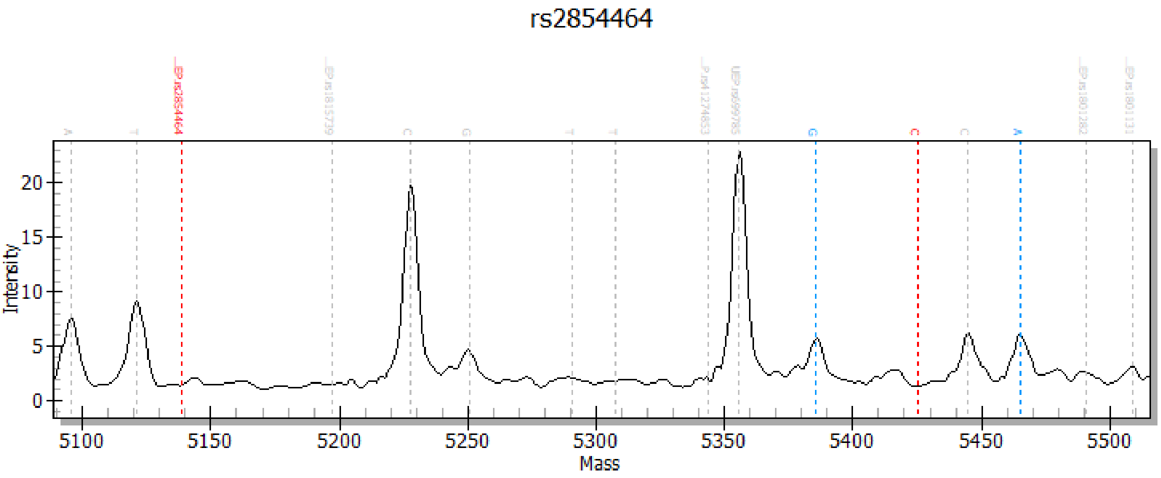

This chromatogram depicts the molecular mass SBE products associated with the detection of heterozygotes G/A alleles in rs2854464 SNP.

Chromatogram 35 (rs2854464 A allele)

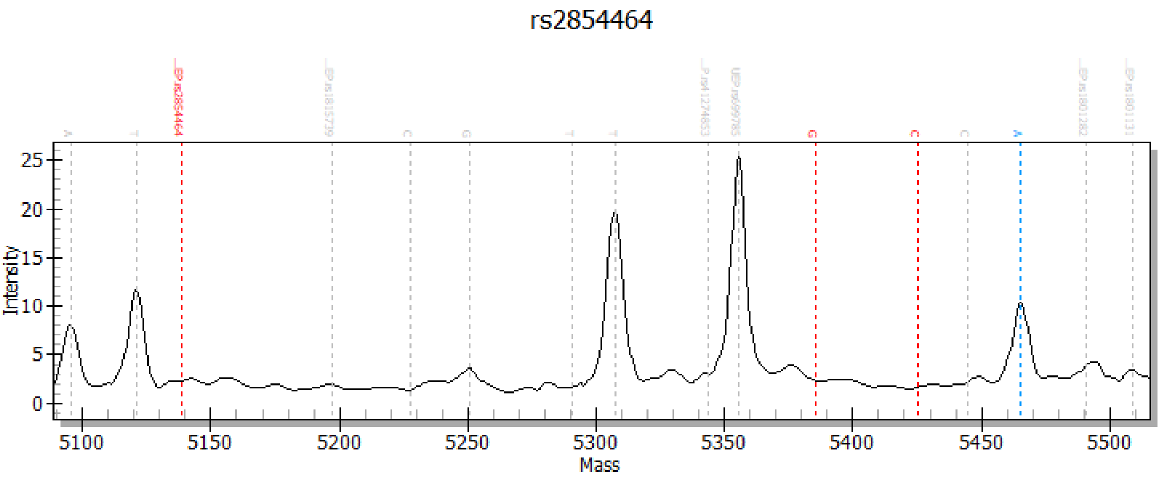

This chromatogram depicts the molecular mass SBE products associated with the detection of A allele in rs2854464 SNP.

Chromatogram 36 (rs2920503 T allele)

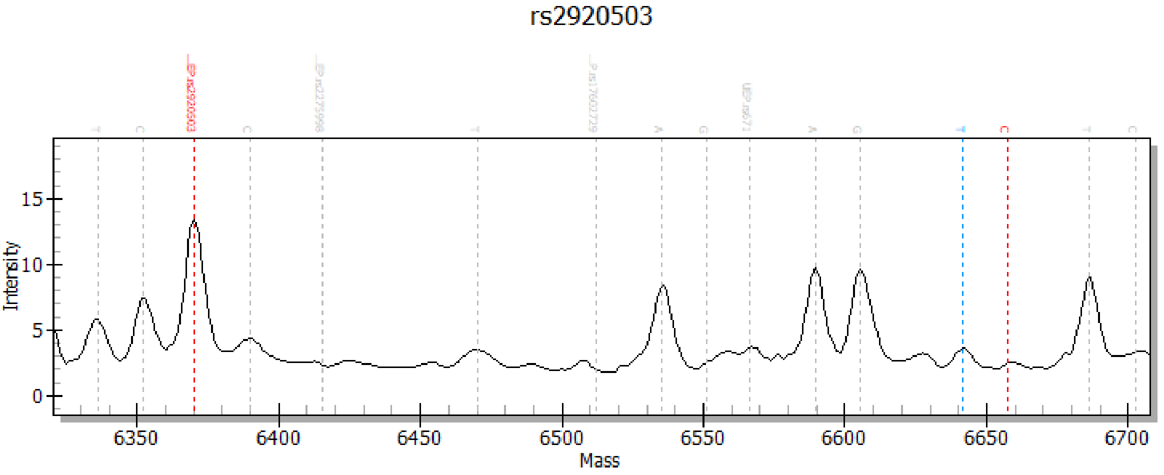

This chromatogram depicts the molecular mass SBE products associated with the detection of T allele in rs2920503 SNP.

Chromatogram 37 (rs2920503 C allele)

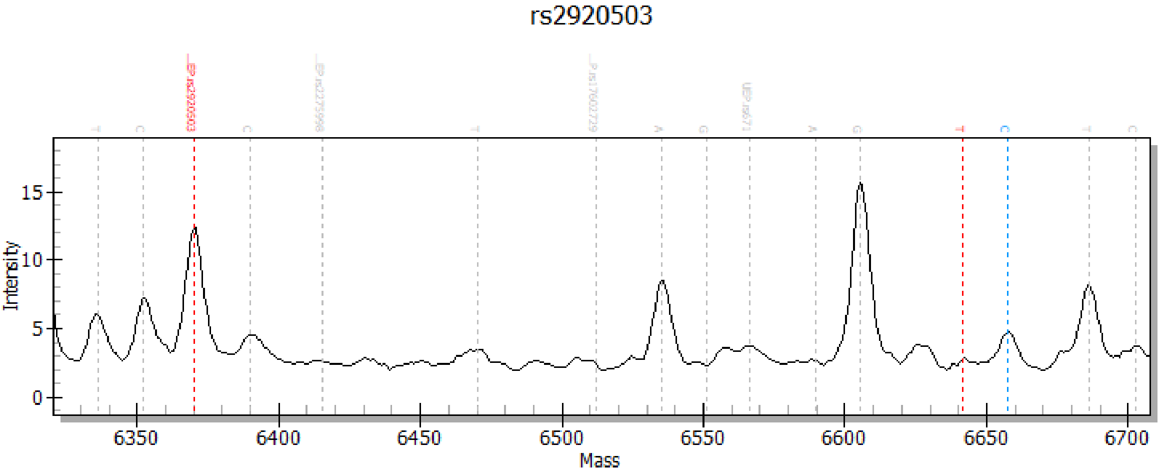

This chromatogram depicts the molecular mass SBE products associated with the detection of C allele in rs2920503 SNP.

Chromatogram 38 (rs303760 T allele)

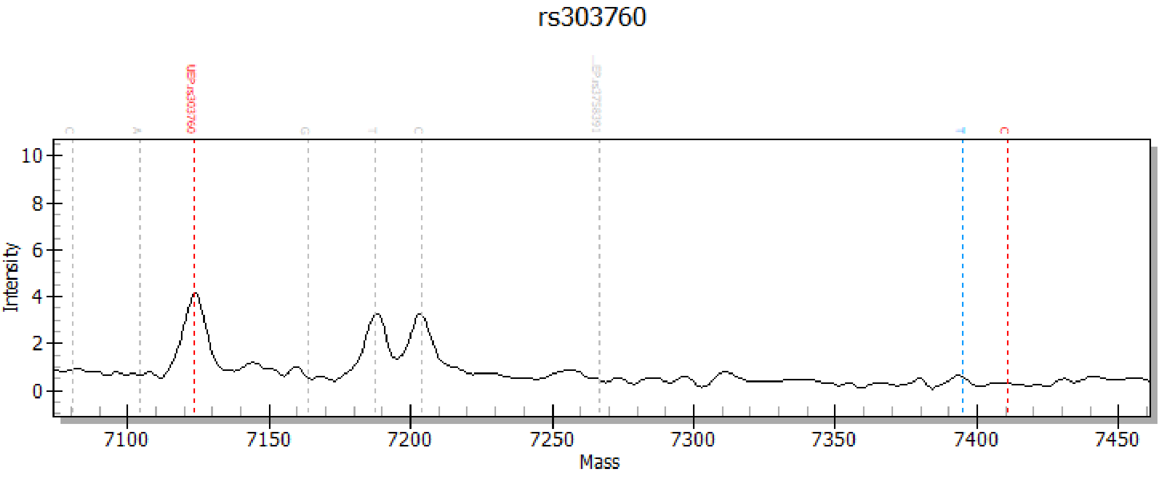

This chromatogram depicts the molecular mass SBE products associated with the detection of T allele in rs303760 SNP.

Chromatogram 39 (rs3213537 C allele)

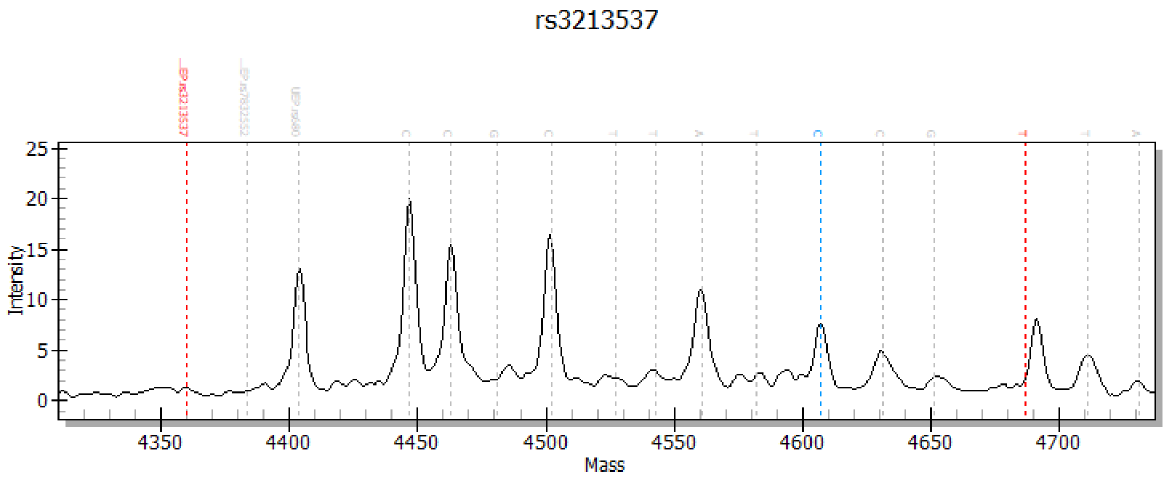

This chromatogram depicts the molecular mass SBE products associated with the detection of C allele in rs3213537 SNP.

Chromatogram 40 (rs3213537 C/T alleles)

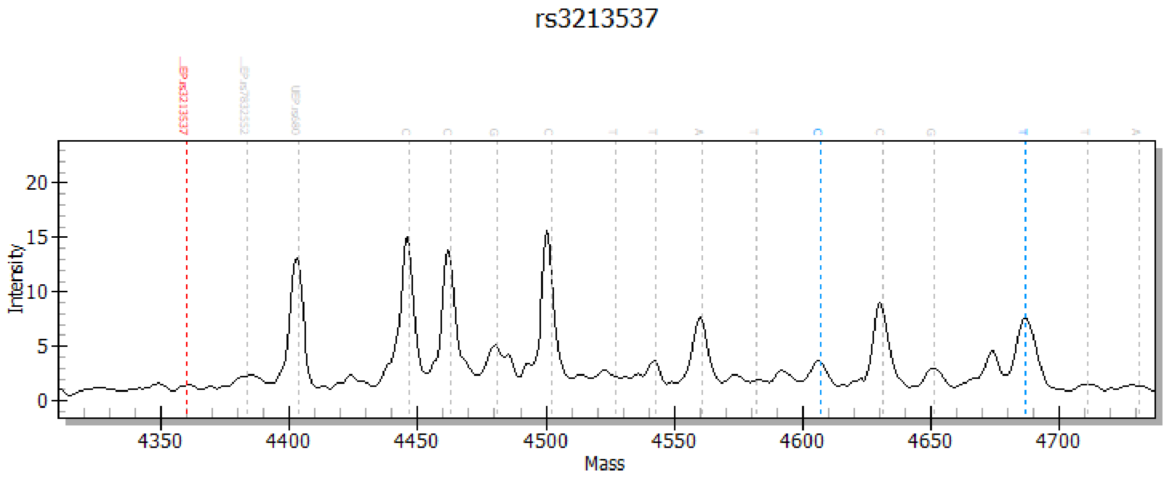

This chromatogram depicts the molecular mass SBE products associated with the detection of heterozigotes C/T alleles in rs3213537 SNP.

Chromatogram 41 (rs3758391 C allele)

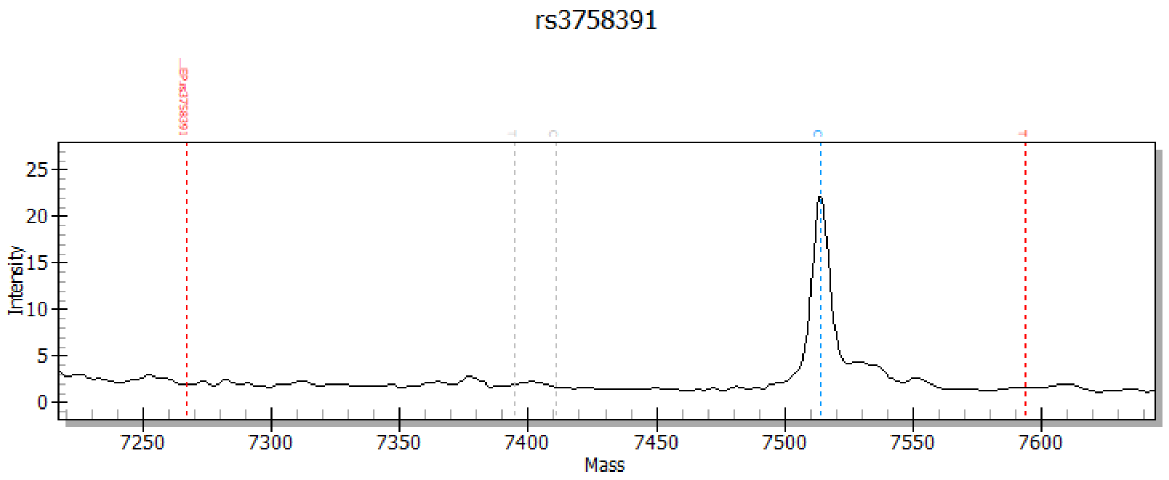

This chromatogram depicts the molecular mass SBE products associated with the detection of C alleles in rs3758391 SNP.

Chromatogram 42 (rs3758391 C/T allele)

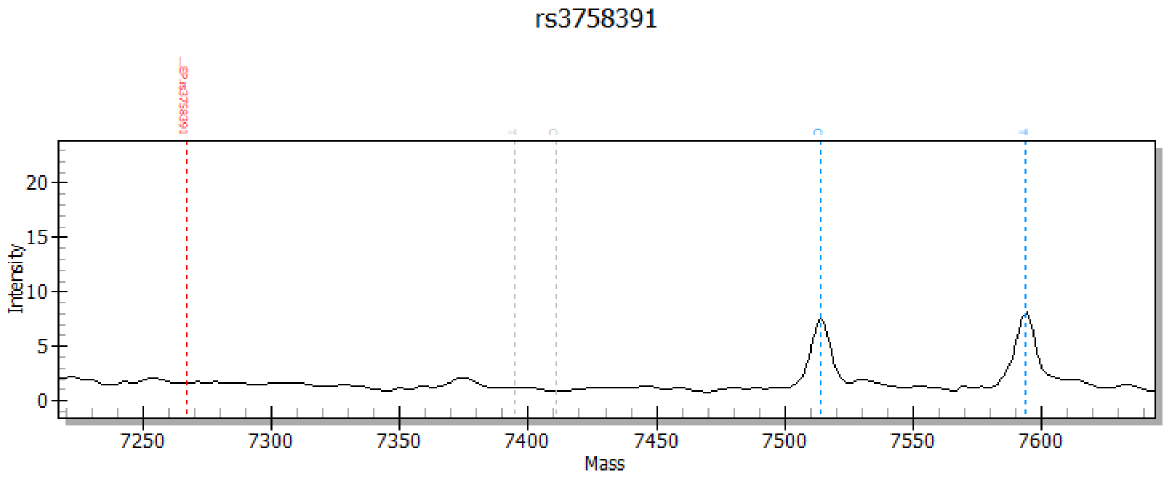

This chromatogram depicts the molecular mass SBE products associated with the detection of heterozygotes C/T alleles in rs3758391 SNP.

Chromatogram 43 (rs3758391 T allele)

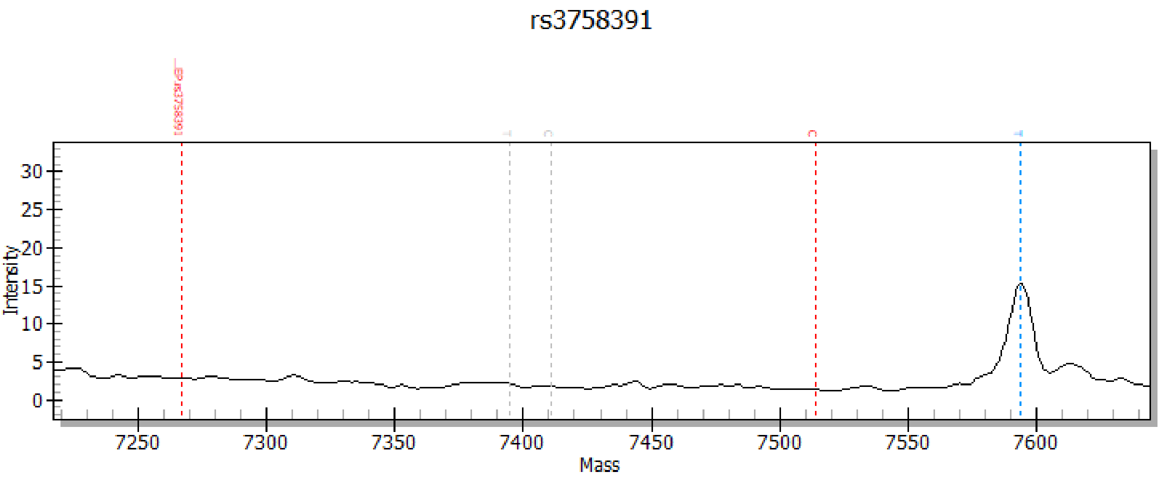

This chromatogram depicts the molecular mass SBE products associated with the detection of T allele in rs3758391 SNP.

Chromatogram 44 (rs4074992 C allele)

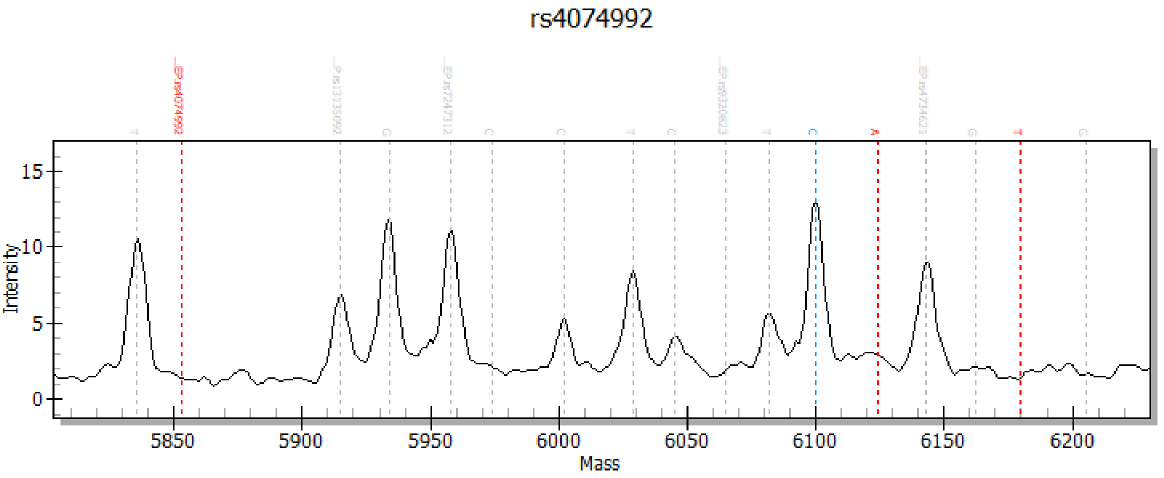

This chromatogram depicts the molecular mass SBE products associated with the detection of C alleles in rs4074992 SNP.

Chromatogram 45 (rs4074992 C/T alleles)

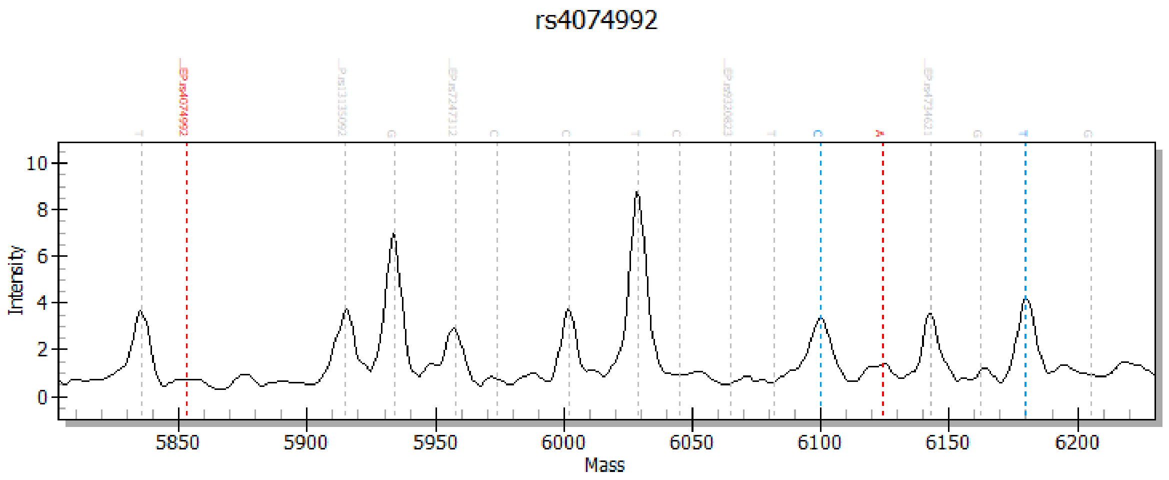

This chromatogram depicts the molecular mass SBE products associated with the detection of heterozygotes C /T alleles in rs4074992 SNP.

Chromatogram 46 (rs4074992 T allele)

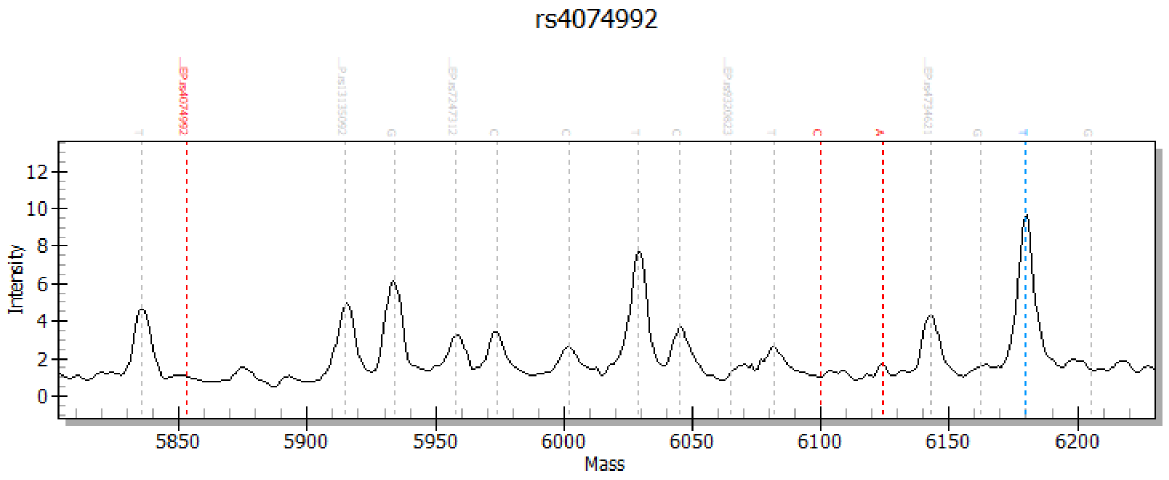

This chromatogram depicts the molecular mass SBE products associated with the detection of T allele in rs4074992 SNP.

Chromatogram 47 (rs41274853 G allele)

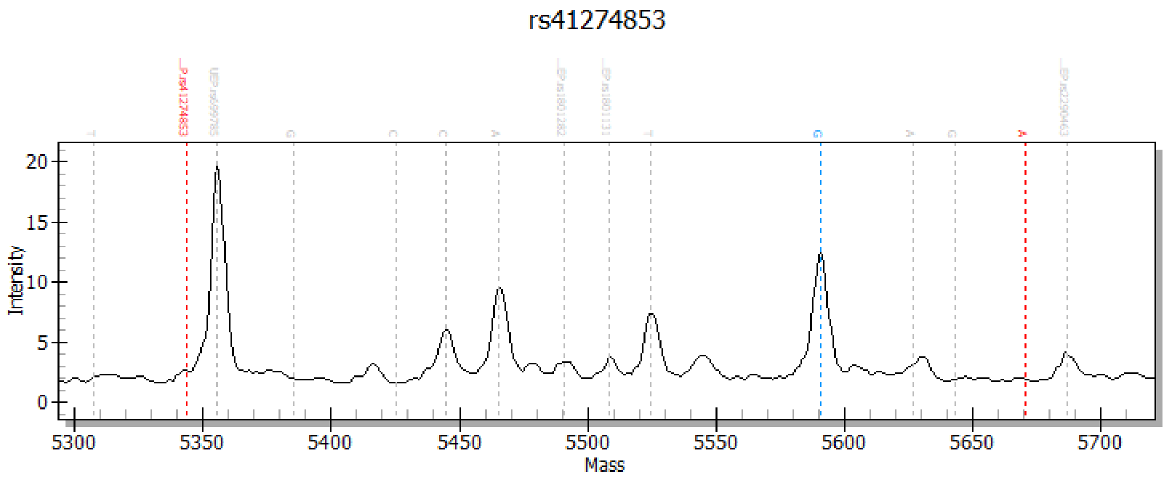

This chromatogram depicts the molecular mass SBE products associated with the detection of G allele in rs41274853 SNP.

Chromatogram 48 (rs41274853 G/A alleles)

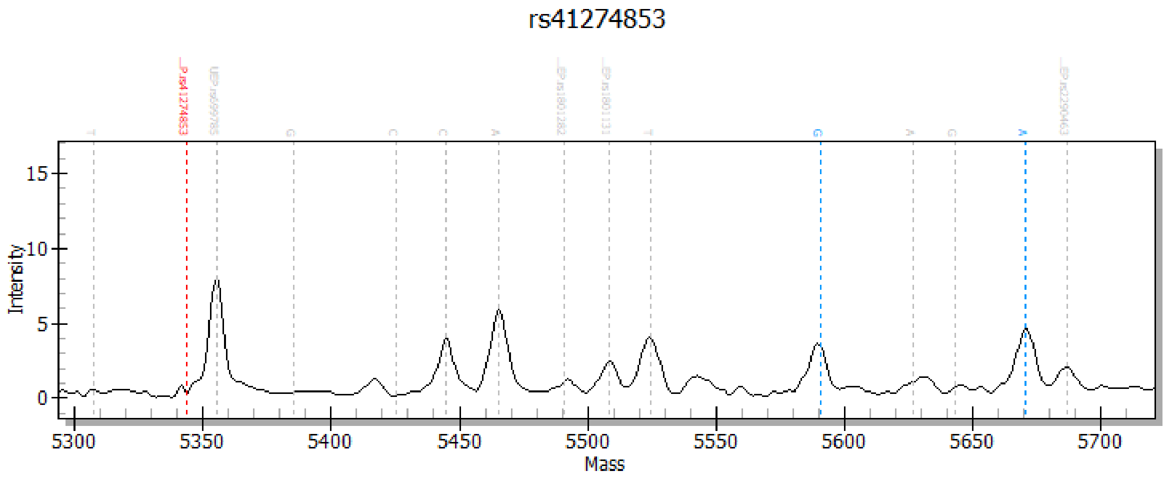

This chromatogram depicts the molecular mass SBE products associated with the detection of heterozygotes G/A alleles in rs41274853 SNP.

Chromatogram 49 (rs41274853 A allele)

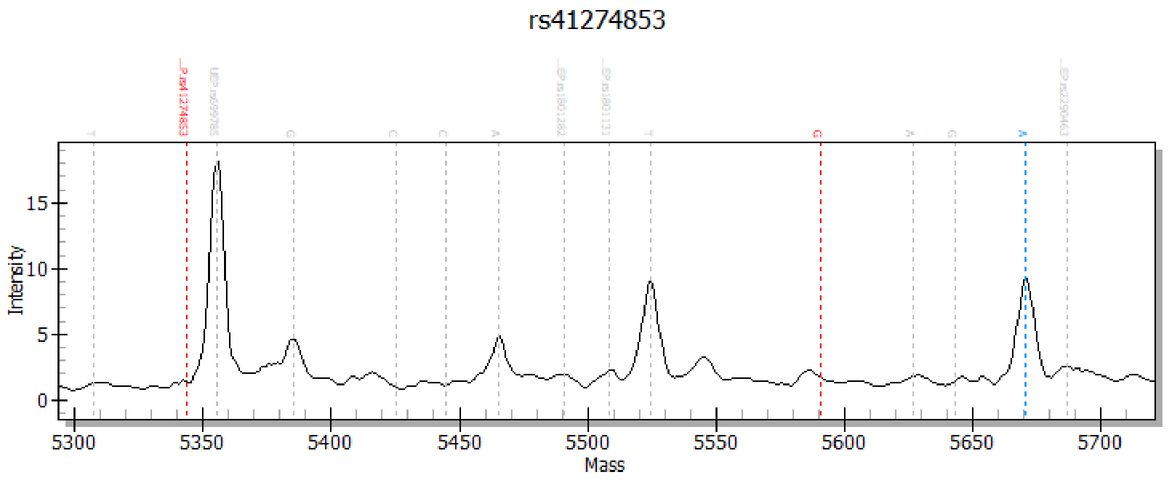

This chromatogram depicts the molecular mass SBE products associated with the detection of A allele in rs41274853 SNP.

Chromatogram 50 (rs4253778 G allele)

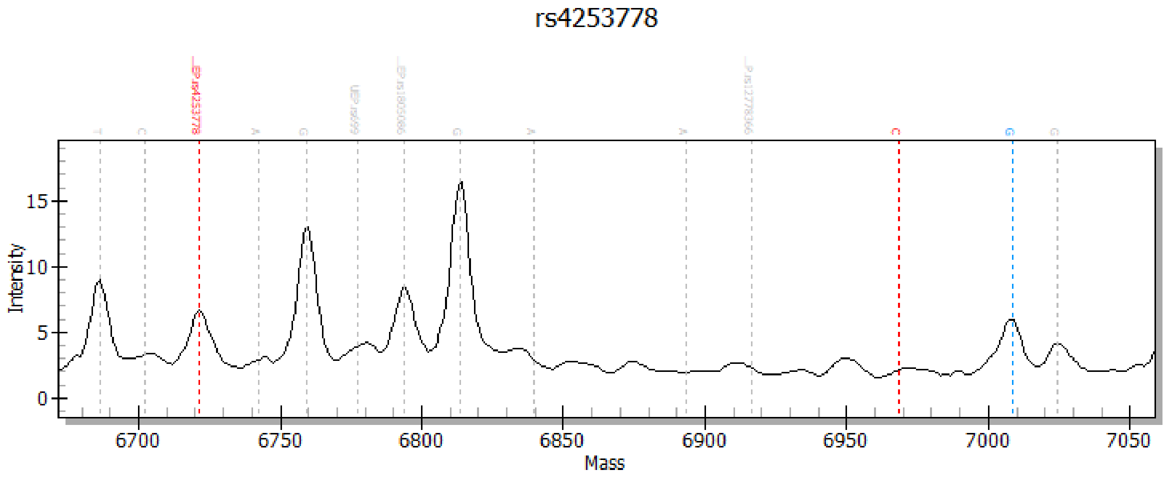

This chromatogram depicts the molecular mass SBE products associated with the detection of G allele in rs4253778 SNP.

Chromatogram 51 (rs4734621 C allele)

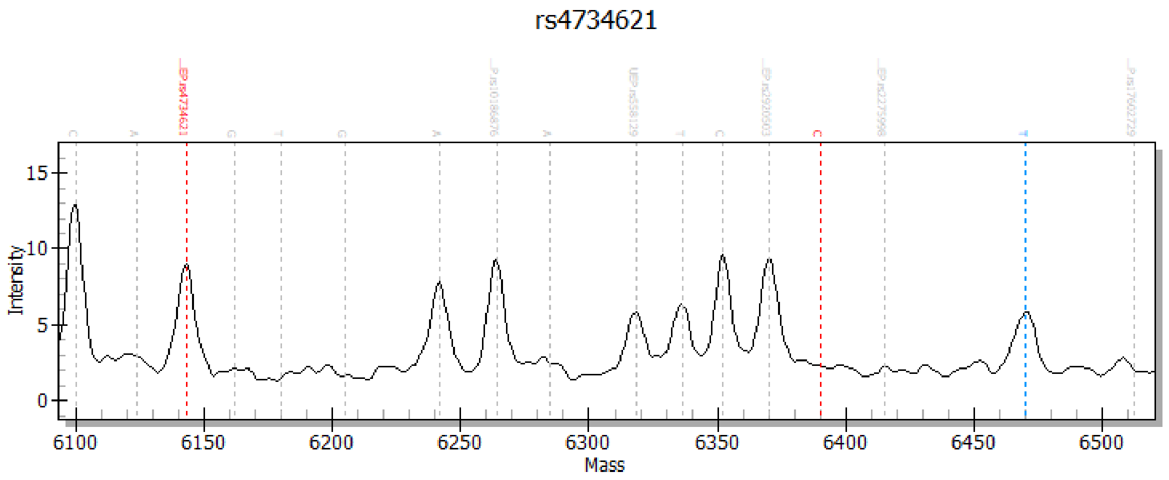

This chromatogram depicts the molecular mass SBE products associated with the detection of C allele in rs4734621 SNP.

Chromatogram 52 (rs4734621 C/T alleles)

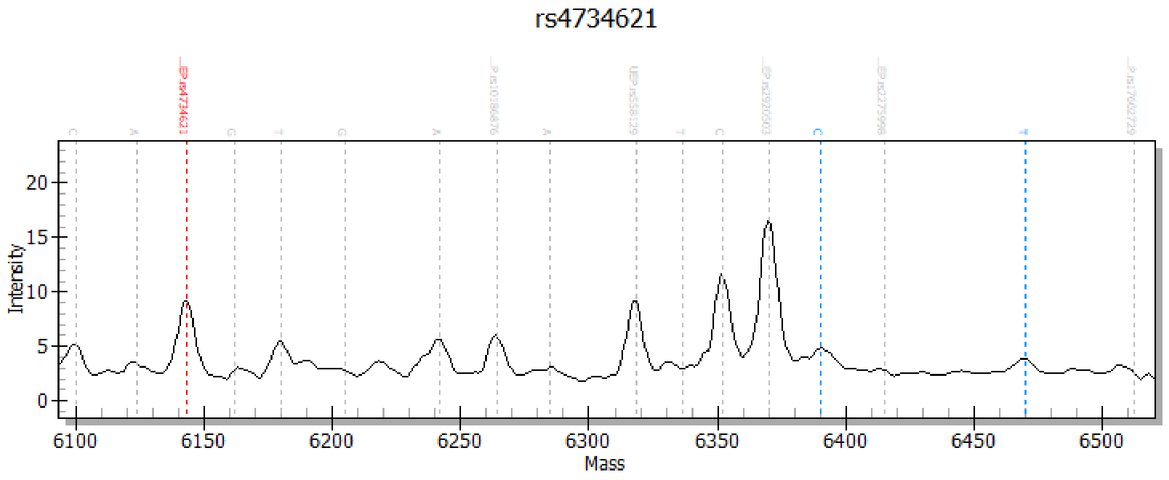

This chromatogram depicts the molecular mass SBE products associated with the detection of heterozygotes C/T alleles in rs4734621 SNP.

Chromatogram 53 (rs4734621 T allele)

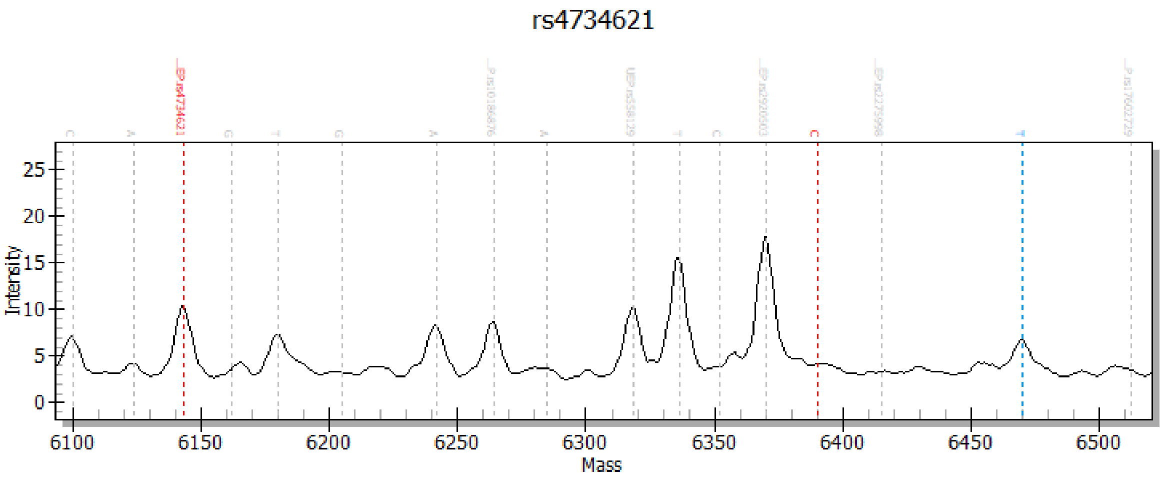

This chromatogram depicts the molecular mass SBE products associated with the detection of T allele in rs4734621 SNP.

Chromatogram 54 (rs55743914 C allele)

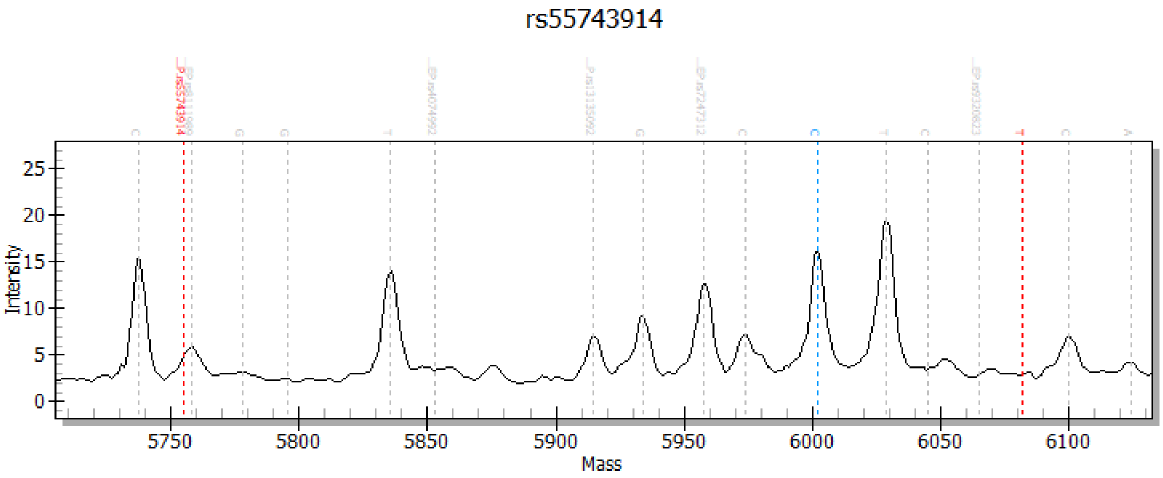

This chromatogram depicts the molecular mass SBE products associated with the detection of C allele in rs55743914 SNP.

Chromatogram 55 (rs55743914 C/T alleles)

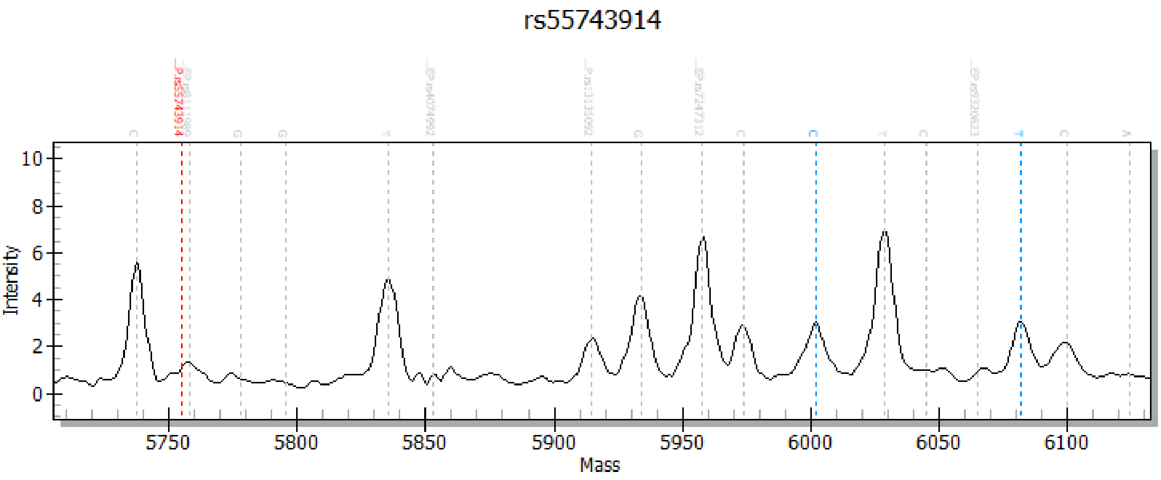

This chromatogram depicts the molecular mass SBE products associated with the detection of heterozygotes C/T alleles in rs55743914 SNP.

Chromatogram 56 (rs558129 A allele)

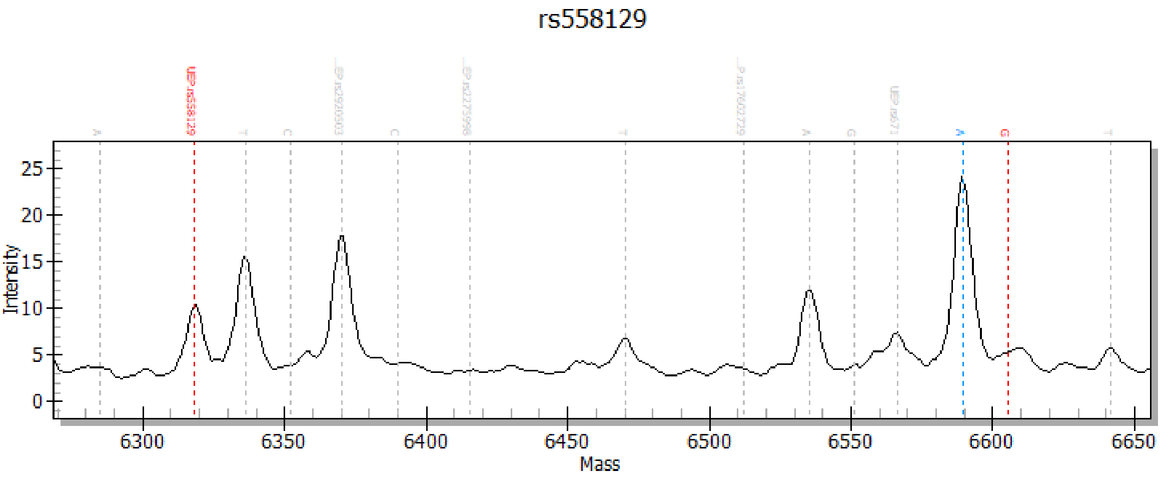

This chromatogram depicts the molecular mass SBE products associated with the detection of A allele in rs558129 SNP.

Chromatogram 57 (rs558129 A/G alleles)

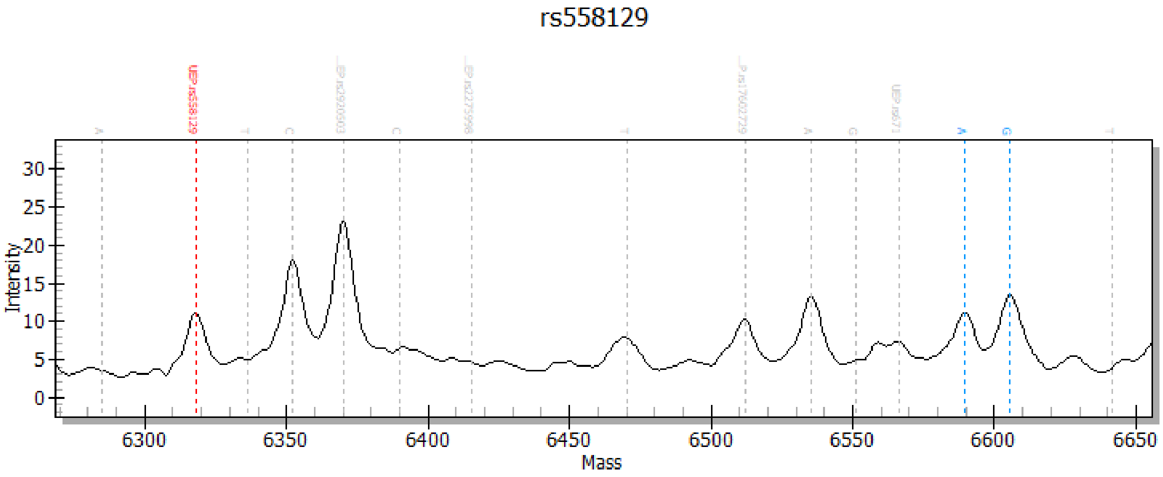

This chromatogram depicts the molecular mass SBE products associated with the detection of heterozygotes A/G alleles in rs558129 SNP.

Chromatogram 58 (rs558129 G allele)

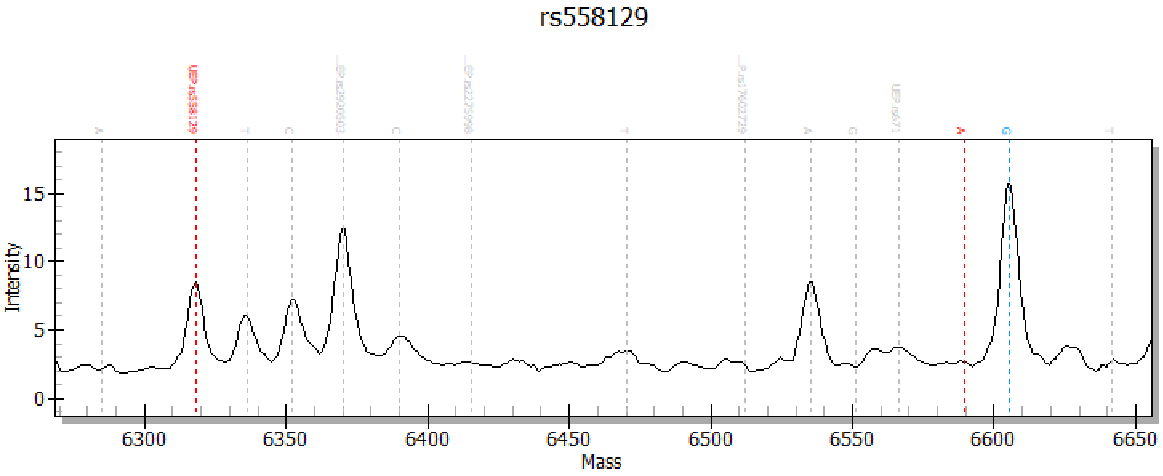

This chromatogram depicts the molecular mass SBE products associated with the detection of G allele in rs558129 SNP.

Chromatogram 59 (rs56068671 G allele)

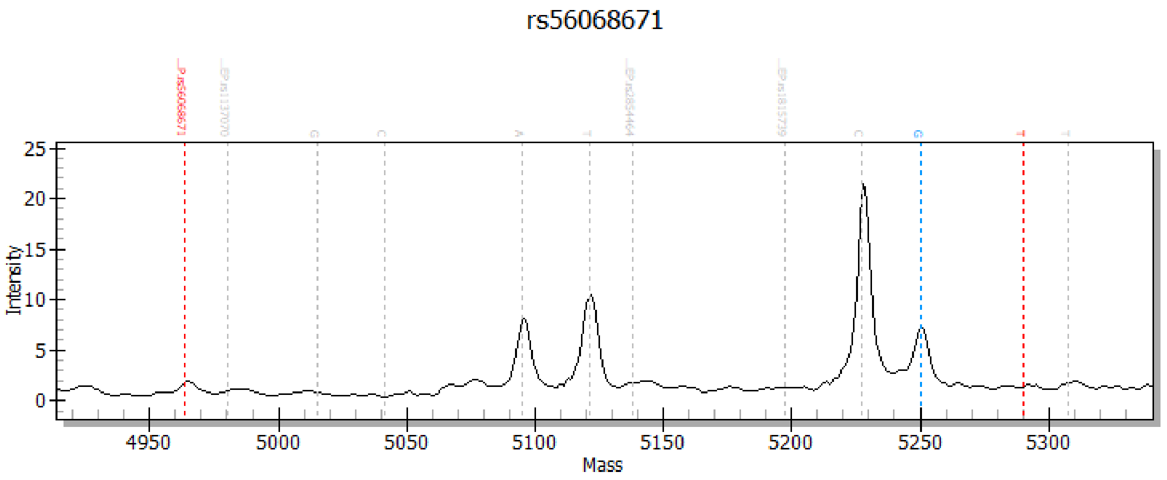

This chromatogram depicts the molecular mass SBE products associated with the detection of G allele in rs56068671 SNP.

Chromatogram 60 (rs671 G allele)

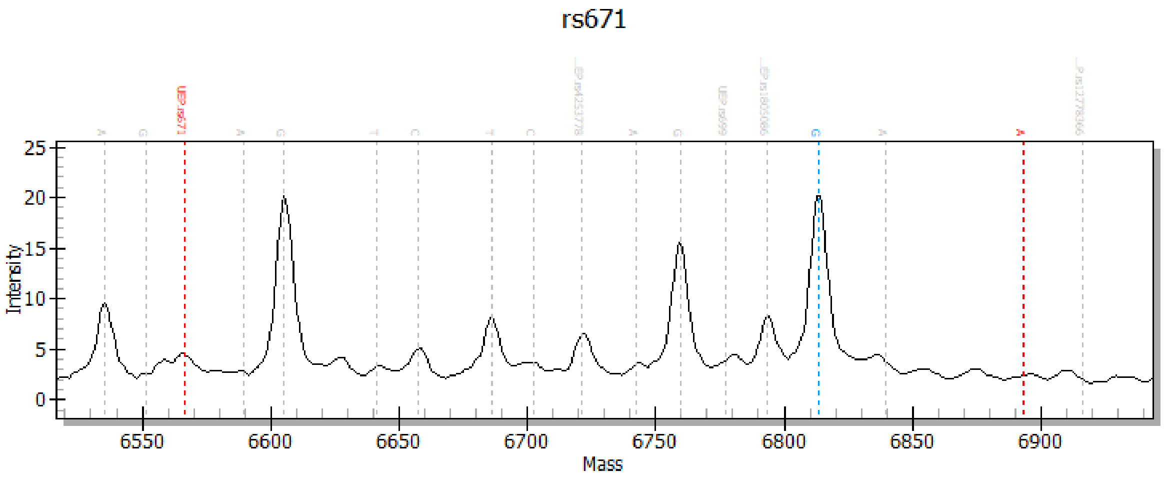

This chromatogram depicts the molecular mass SBE products associated with the detection of G allele in rs671 SNP.

Chromatogram 61 (rs671 G/A alleles)

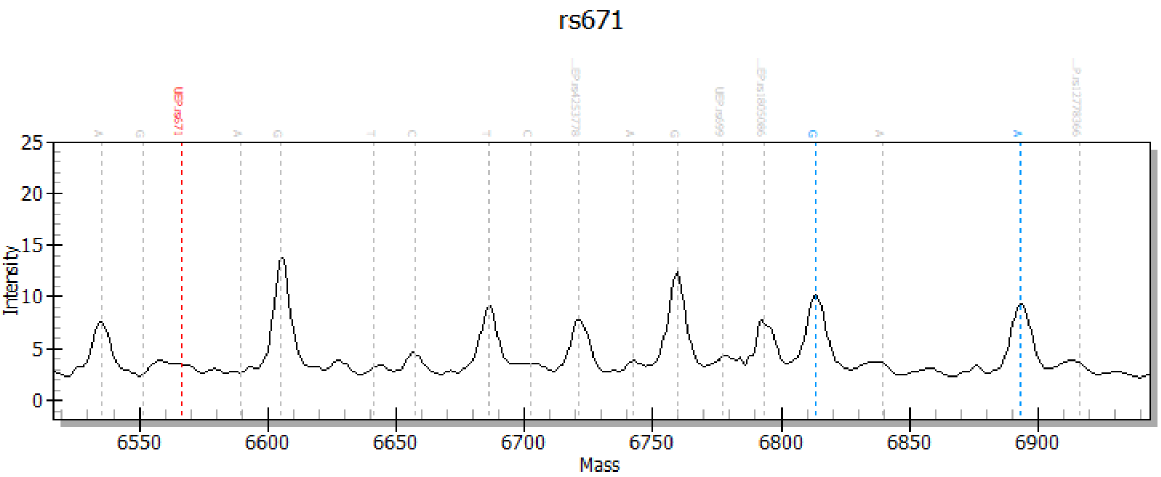

This chromatogram depicts the molecular mass SBE products associated with the detection of heterozygotes G/A alleles in rs671 SNP.

Chromatogram 62 (rs680 G allele)

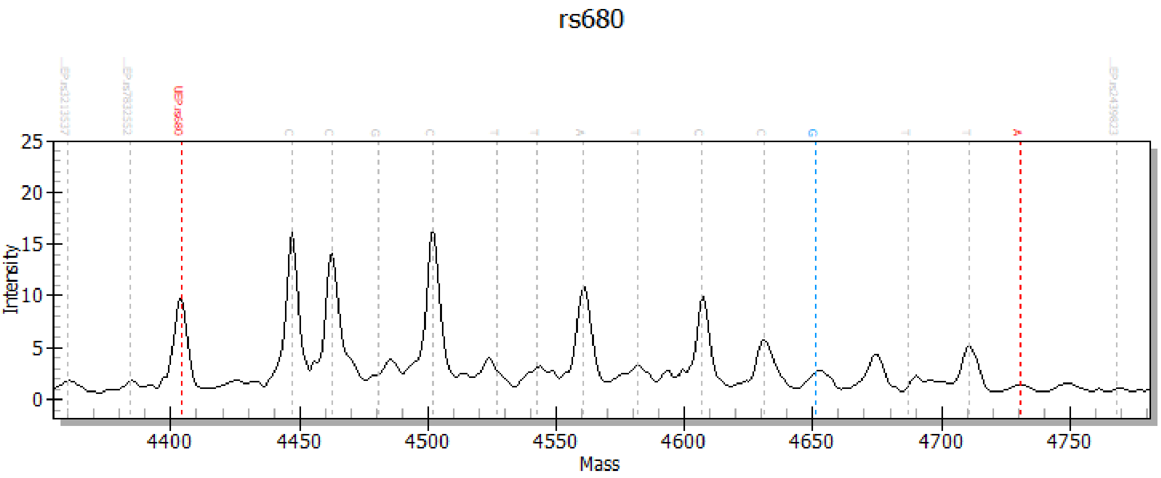

This chromatogram depicts the molecular mass SBE products associated with the detection of G allele in rs680 SNP.

Chromatogram 63 (rs680 G/A alleles)

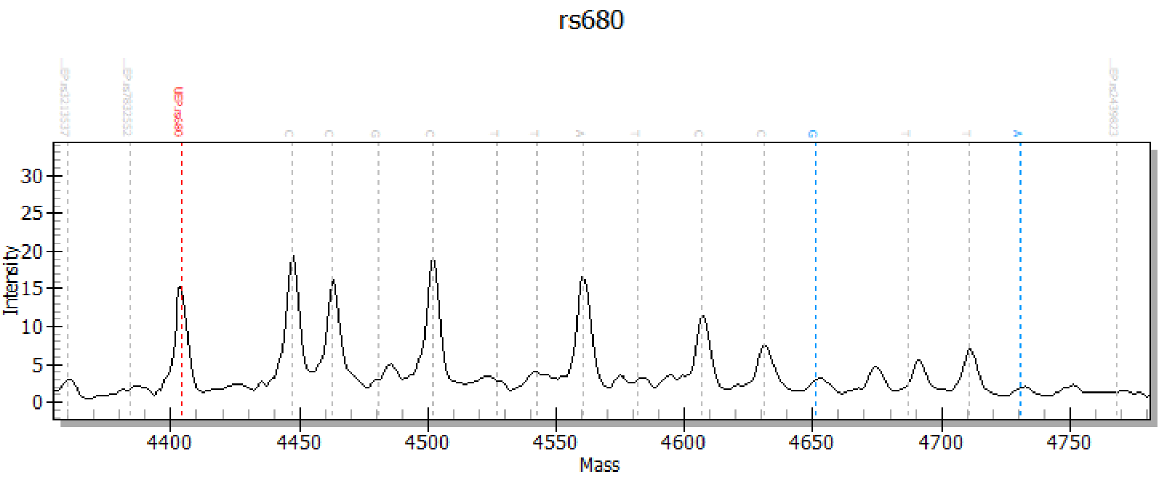

This chromatogram depicts the molecular mass SBE products associated with the detection of heterozygotes G/A alleles in rs680 SNP.

Chromatogram 64 (rs6905419 C allele)

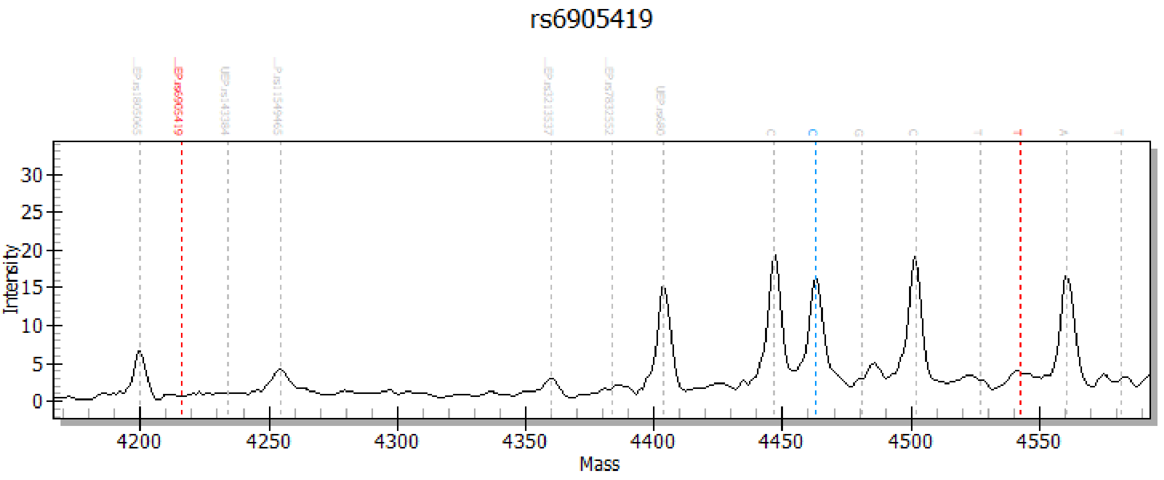

This chromatogram depicts the molecular mass SBE products associated with the detection of C allele in rs6905419 SNP.

Chromatogram 65 (rs699 G allele)

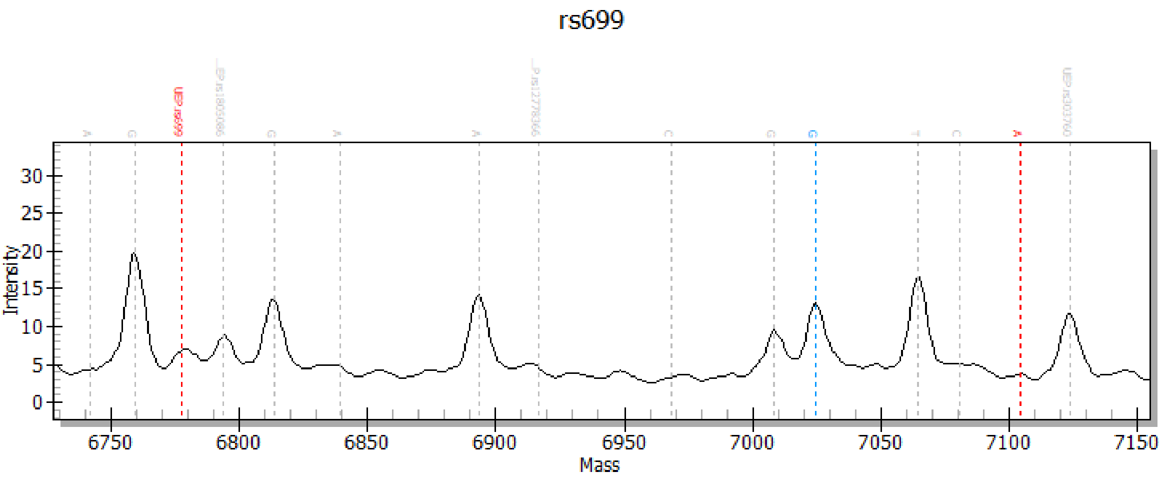

This chromatogram depicts the molecular mass SBE products associated with the detection of G allele in rs699 SNP.

Chromatogram 66 (rs699 G/A alleles)

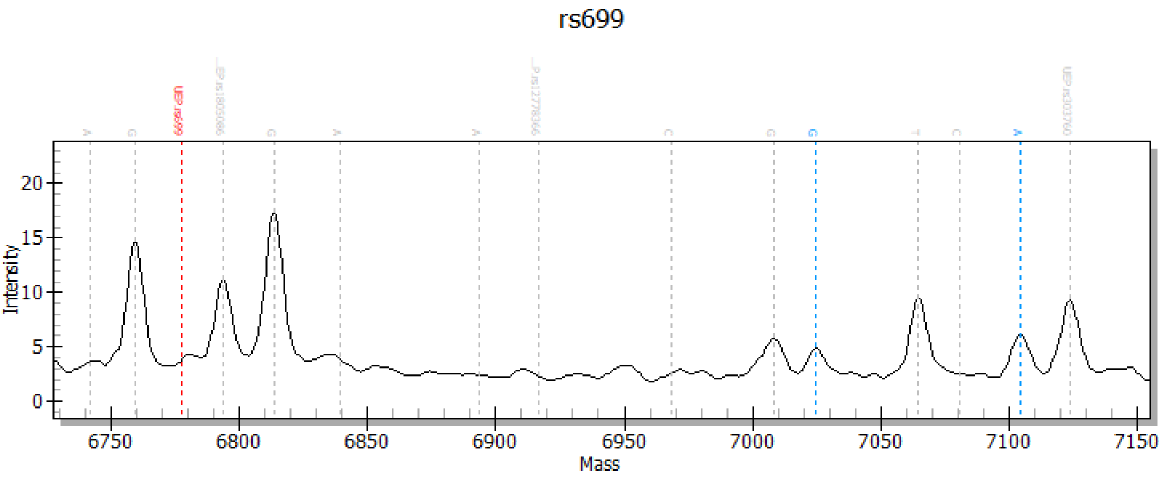

This chromatogram depicts the molecular mass SBE products associated with the detection of heterozygotes G/A alleles in rs699 SNP.

Chromatogram 67 (rs699 A allele)

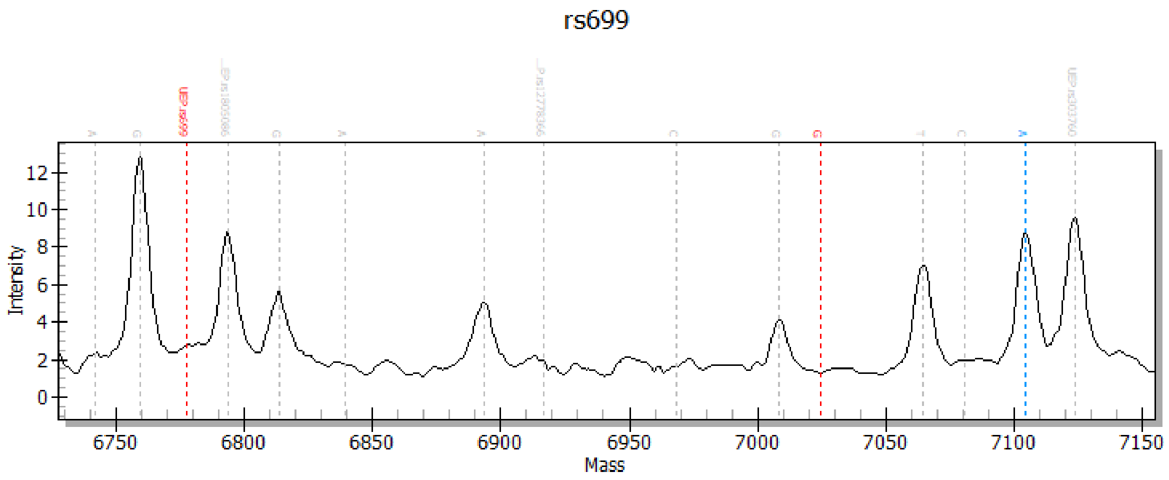

This chromatogram depicts the molecular mass SBE products associated with the detection of A allele in rs699 SNP.

Chromatogram 68 (rs699785 A allele)

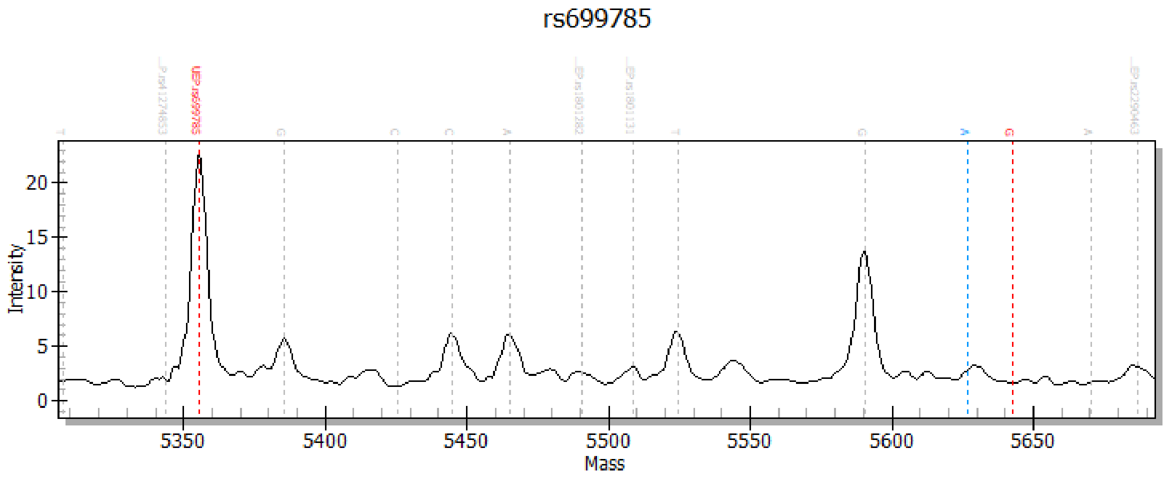

This chromatogram depicts the molecular mass SBE products associated with the detection of A allele in rs699785 SNP.

Chromatogram 69 (rs7247312 G allele)

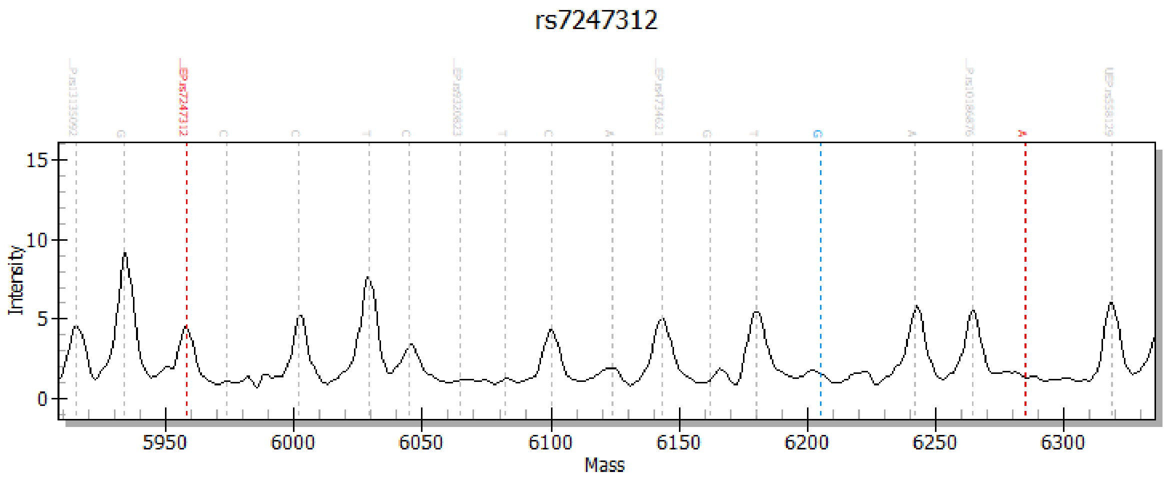

This chromatogram depicts the molecular mass SBE products associated with the detection of G allele in rs7247312 SNP.

Chromatogram 70 (rs7247312 G/A alleles)

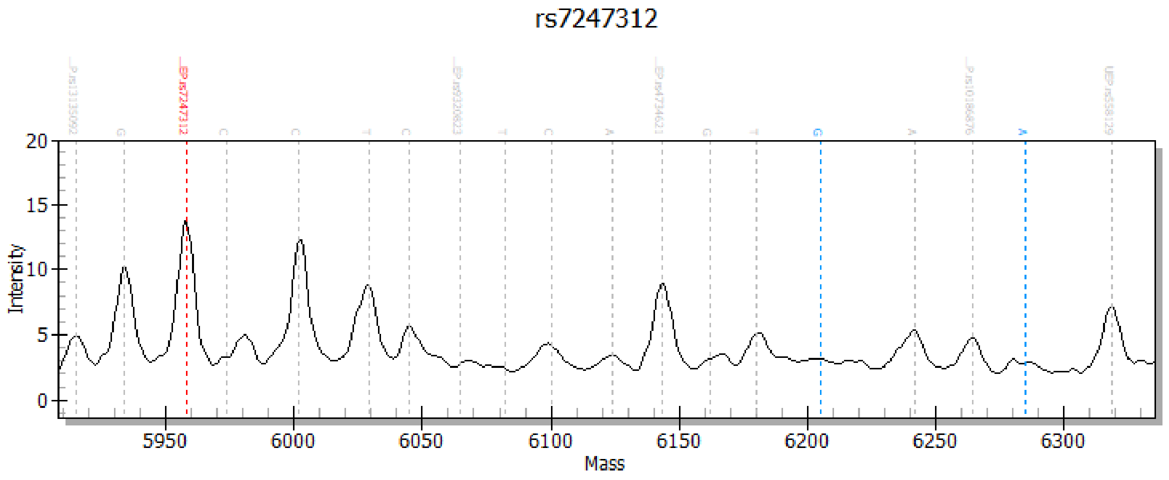

This chromatogram depicts the molecular mass SBE products associated with the detection of heterozygotes G/A alleles in rs7247312 SNP.

Chromatogram 71 (rs7247312 A allele)

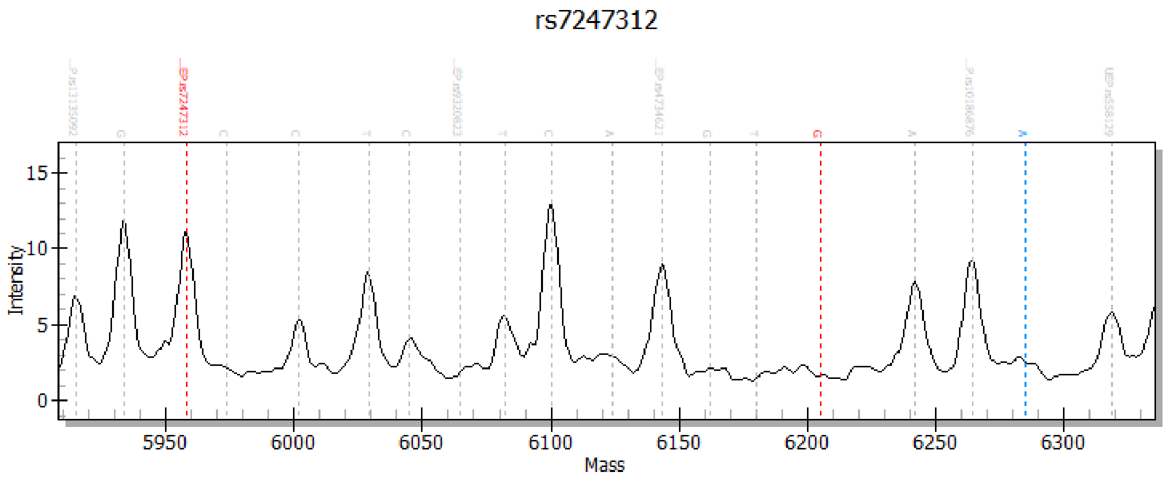

This chromatogram depicts the molecular mass SBE products associated with the detection of A allele in rs7247312 SNP.

Chromatogram 72 (rs7832552 C allele)

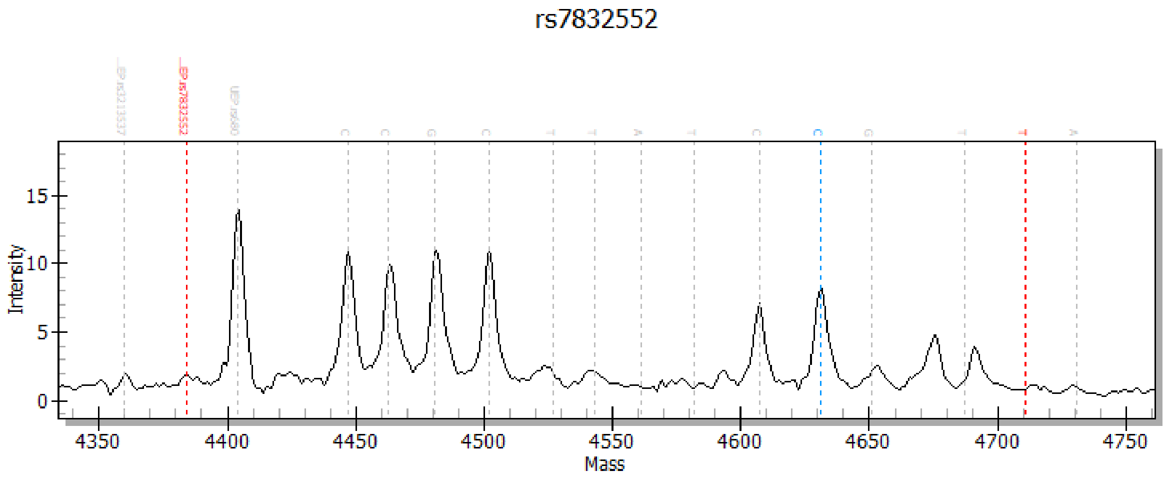

This chromatogram depicts the molecular mass SBE products associated with the detection of C allele in rs7832552 SNP.

Chromatogram 73 (rs7832552 C/T alleles)

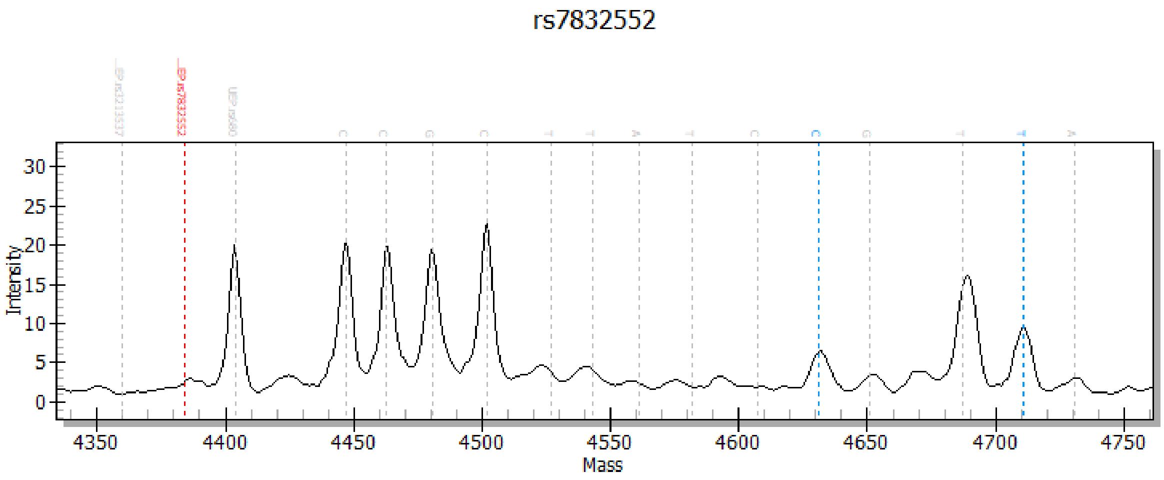

This chromatogram depicts the molecular mass SBE products associated with the detection of heterozygotes C/T alleles in rs7832552 SNP.

Chromatogram 74 (rs7832552 T allele)

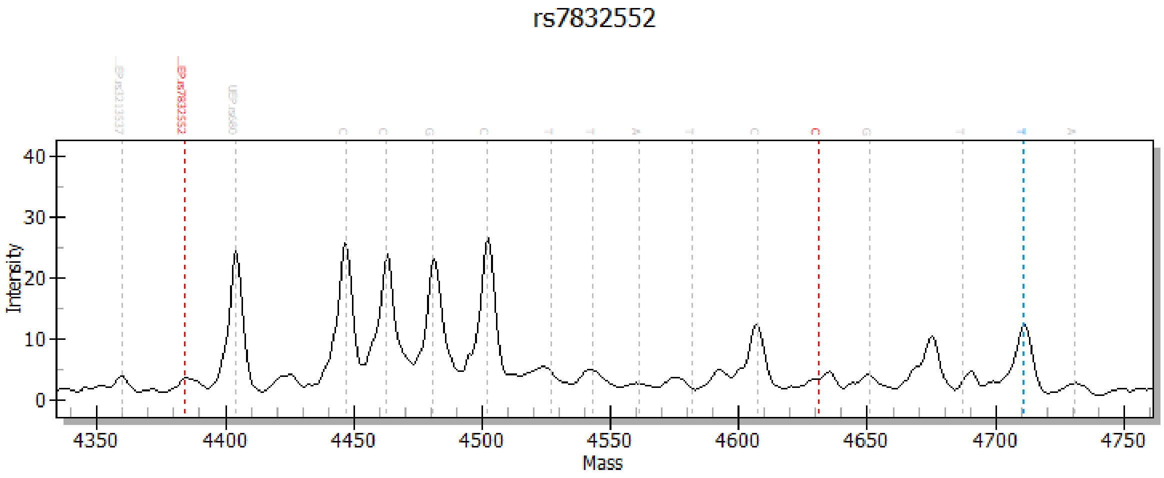

This chromatogram depicts the molecular mass SBE products associated with the detection of T allele in rs7832552 SNP.

Chromatogram 75 (rs8111989 T allele)

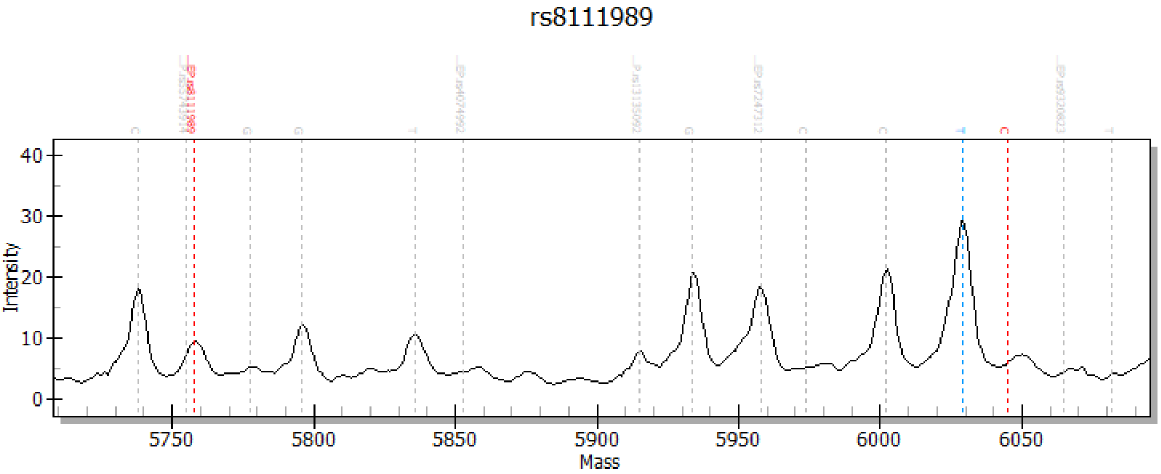

This chromatogram depicts the molecular mass SBE products associated with the detection of T allele in rs8111989 SNP.

Chromatogram 76 (rs8111989 T/C alleles)

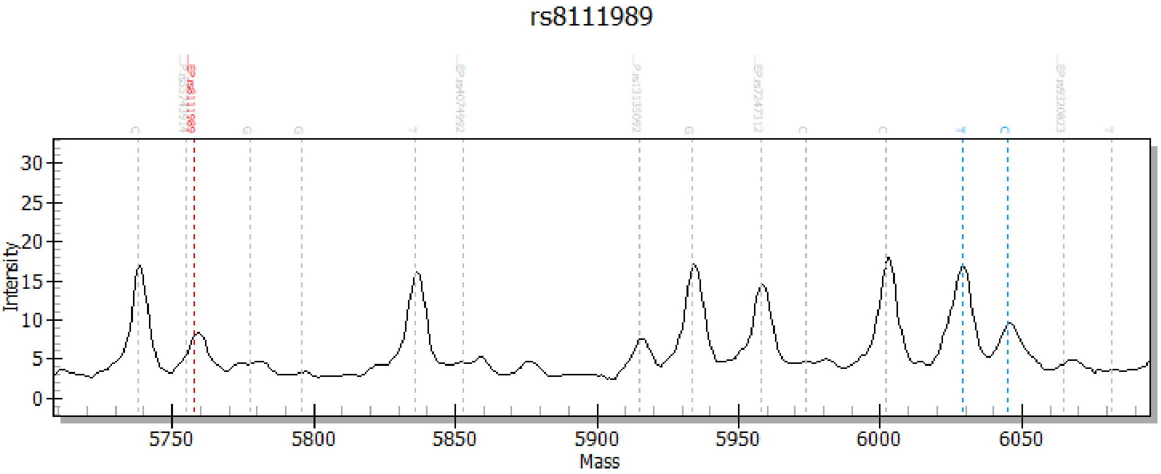

This chromatogram depicts the molecular mass SBE products associated with the detection of heterozygotes T/C alleles in rs8111989 SNP.

Chromatogram 77 (rs8111989 C allele)

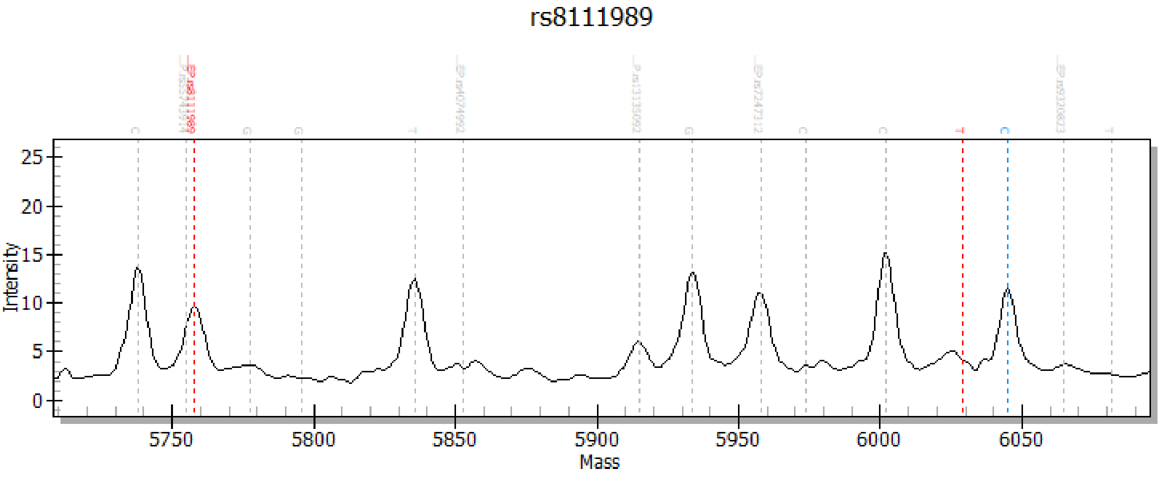

This chromatogram depicts the molecular mass SBE products associated with the detection of C allele in rs8111989 SNP.

Chromatogram 78 (rs9320823 C allele)

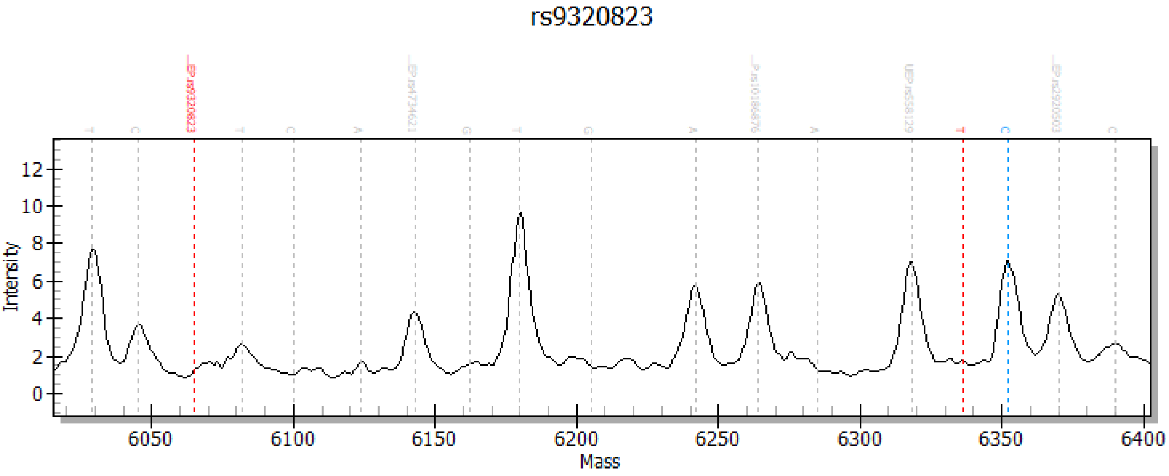

This chromatogram depicts the molecular mass SBE products associated with the detection of C allele in rs9320823 SNP.

Chromatogram 79 (rs9320823 T/C alleles)

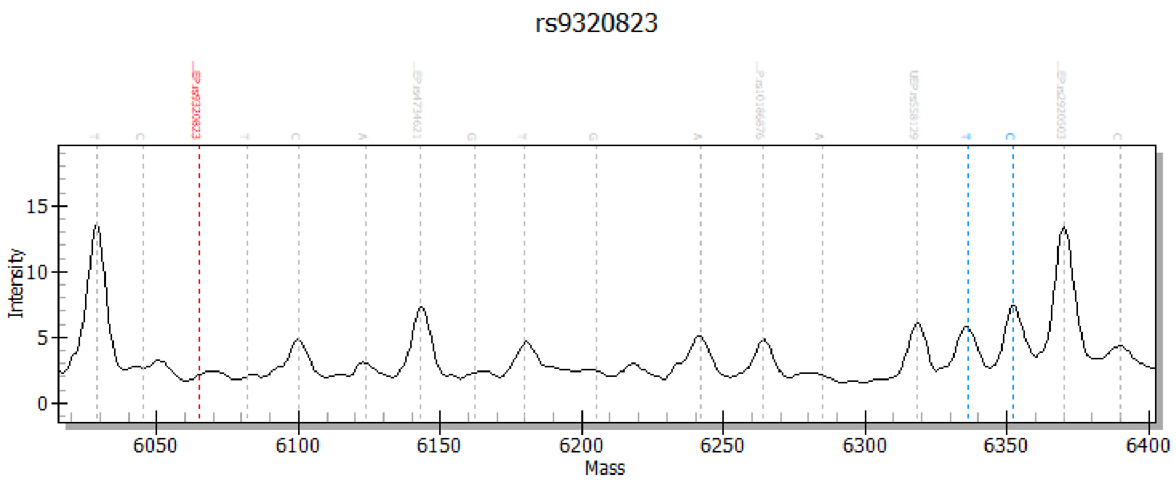

This chromatogram depicts the molecular mass SBE products associated with the detection of heterozygotes T/C alleles in rs9320823 SNP.

Chromatogram 80 (rs9320823 T allele)

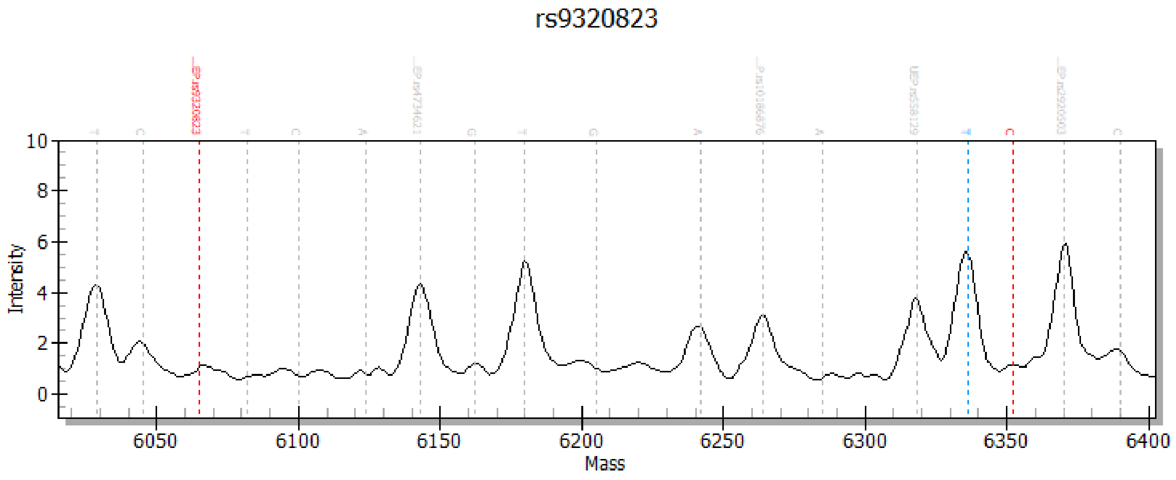

This chromatogram depicts the molecular mass SBE products associated with the detection of T allele in rs9320823 SNP.
